# Post‐mortem multiple sclerosis lesion pathology is influenced by single nucleotide polymorphisms

**DOI:** 10.1111/bpa.12760

**Published:** 2019-07-23

**Authors:** Nina L. Fransen, Jakob B. A. Crusius, Joost Smolders, Mark R. Mizee, Corbert G. van Eden, Sabina Luchetti, Ester B. M. Remmerswaal, Jörg Hamann, Matthew R. J. Mason, Inge Huitinga

**Affiliations:** ^1^ Department of Neuroimmunology The Netherlands Institute for Neuroscience Amsterdam The Netherlands; ^2^ Laboratory for Immunogenetics, Department of Medical Microbiology and Infection Control Amsterdam UMC, VU University Amsterdam The Netherlands; ^3^ MS Center CWZ, Department of Neurology Canisius Wilhelmina Hospital Nijmegen The Netherlands; ^4^ Department of Experimental Immunology, Amsterdam Infection & Immunity Institute Amsterdam UMC, University of Amsterdam Amsterdam The Netherlands; ^5^ Renal Transplant Unit, Department of Internal Medicine, Amsterdam Infection & Immunity Institute Amsterdam UMC, University of Amsterdam Amsterdam The Netherlands

**Keywords:** CLEC16A, FAS, multiple sclerosis, NCAN, neuropathology

## Abstract

Over the last few decades, several common single nucleotide polymorphisms (SNPs) have been identified that correlate with clinical outcome in multiple sclerosis (MS), but the pathogenic mechanisms underlying the clinical effects of these SNPs are unknown. This is in part because of the difficulty in the functional translation of genotype into disease‐relevant mechanisms. Building on our recent work showing the association of clinical disease course with post‐mortem MS lesion characteristics, we hypothesized that SNPs that correlate with clinical disease course would also correlate with specific MS lesion characteristics in autopsy tissue. To test this hypothesis, 179 MS brain donors from the Netherlands Brain Bank MS autopsy cohort were genotyped for 102 SNPs, selected based on their reported associations with clinical outcome or their associations with genes that show differential gene expression in MS lesions. Three SNPs linked to MS clinical severity showed a significant association between the genotype and either the proportion of active lesions (rs2234978/FAS and rs11957313/KCNIP1) or the proportion of mixed active/inactive lesions (rs8056098/CLEC16A). Three SNPs linked to MS pathology‐associated genes showed a significant association with either proportion of active lesions (rs3130253/MOG), incidence of cortical gray matter lesions (rs1064395/NCAN) or the proportion of remyelinated lesions (rs5742909/CTLA4). In addition, rs2234978/FAS T‐allele carriers showed increased FAS gene expression levels in perivascular T cells and perilesional oligodendrocytes, cell types that have been implicated in MS lesion formation. Thus, by combining pathological characterization of MS brain autopsy tissue with genetics, we now start to translate genotypes linked to clinical outcomes in MS into mechanisms involved in MS lesion pathogenesis.

## Introduction

Multiple sclerosis (MS) is a heterogeneous disease with large inter‐individual differences in disease course and response to immunomodulatory therapies 40, 41. The pathogenic mechanisms underlying these differences between MS patients remain largely unknown 23, 41. Over the past decades several common genetic variants have been associated with clinical outcome of MS in candidate gene and genome‐wide association studies (GWAS) 4, 6, 7, 58. On their own the identified common genetic variants show a minor effect on the clinical outcome and therefore they have no clinical predictive utility, but nevertheless they possess an important translational potential 3, 26, 27, 32. The genes and associated biological pathways implicated in clinical outcome by genetic association may represent targets for interventions that are likely to have greater effects than the naturally occurring variant 28, 32.

However, the genes and biological pathways associated with the identified variants remain largely unknown because of the inability to translate genotype into disease‐relevant mechanisms 3, 26, 28. Here, by quantitative pathological characterization and gene expression studies of autopsy tissue, we aim to translate genotypic information into pathogenic mechanisms.

Recently, we and others showed that MS lesion characteristics in autopsy tissue are associated with the clinical disease course of MS, in which the proportion of mixed active/inactive (chronic active) lesions was significantly associated with disease progression 23, 41. This chronic lesion activity is not identifiable with commonly used MRI techniques, although recent 7T MRI and PET‐MRI studies have shown the prognostic potential of these approaches for detection and quantification of these mixed active/inactive lesions in living MS patients 15, 42.

Here, we have genotyped 179 MS brain donors from the Netherlands Brain Bank autopsy cohort for 67 SNPs that were previously associated with clinical or MRI outcomes in GWAS studies 4, 6, 7, 58 and 35 SNPs in genes associated with MS pathology in previous studies, for example genes that were found upregulated in the area around these mixed active/inactive lesions in a microarray analysis 33. We identified six variants that showed a significant association with either the proportion of active, mixed active/inactive or remyelinated lesions or the incidence of cortical gray matter lesions in autopsy tissue, of which one, shows increased mRNA levels in brain autopsy tissue. With these findings we now begin to translate genotypic effects on clinical outcome in MS into pathological mechanisms. Improving our understanding of the pathological mechanisms that underlie the differences in clinical outcomes will help us to identify biomarkers to improve the prognosis and development of therapies in MS patients.

## Materials and Methods

### Donor and tissue characteristics

In total, 179 MS brain donors from the Netherlands Brain Bank (NBB) were included in this study. Informed consent was given by the donors for brain autopsy and for the use of material and clinical data for research purposes, in compliance with national ethical guidelines. The NBB autopsy procedures were approved by the Medical Ethics Committee of the VU University Medical Center, Amsterdam, the Netherlands. The donors came to autopsy between 1990 and 2015. The diagnosis MS was confirmed by a certified neuropathologist (Prof. J.M. Rozemuller or Prof. P. van der Valk, VU University Medical Center, Amsterdam, The Netherlands). Cases with confounding CNS pathology (bleeding, infarction and metastasis) were excluded (n = 6). Tissue was dissected from standard locations in the brainstem and spinal cord. Visible MS plaques were dissected during autopsy and since 2001 MS lesions were also dissected on post‐mortem MRI guidance from 1‐cm thick coronal brain slices. Donor and tissue characteristics are shown in Table 1 and described in Luchetti *et al* 2018 41.

**Table 1:** Donor and tissue characteristics of the post‐mortem tissue investigated in this study. SP indicates secondary progressive and PP primary progressive MS. MS disease course was not reported in 12 cases.

	N cases	Age (mean ± SD)	Disease duration (mean ± SD)	Tissue blocks examined per case (mean ± SD)	Total number of tissue blocks (n)	Total number of lesions (n)
Total	179	62.5 (13.7)	28.5 (13.3)	17.6 (10.8)	3148	8462
SP	99	60.4 (13.4)	29.8 (14.2)	17.8 (11.2)	1766	4955
PP	54	66.0 (13.2)	27.5 (11.9)	17.7 (10.1)	954	2632
Relapsing	14	61.8 (15.1)	24.2 (11.6)	19.1 (9.55)	268	414
Type not determined	12	65.8 (14.9)	24.5 (14.5)	13.3 (11.9)	160	461
Male	68	60.0 (13.5)	27.5 (12.9)	19.1 (10.5)	1297	3532
Female	111	64.1 (13.7)	29.1 (13.6)	16.7 (10.9)	1851	4930

### Pathological characterization of MS lesions

**Figure 1:**
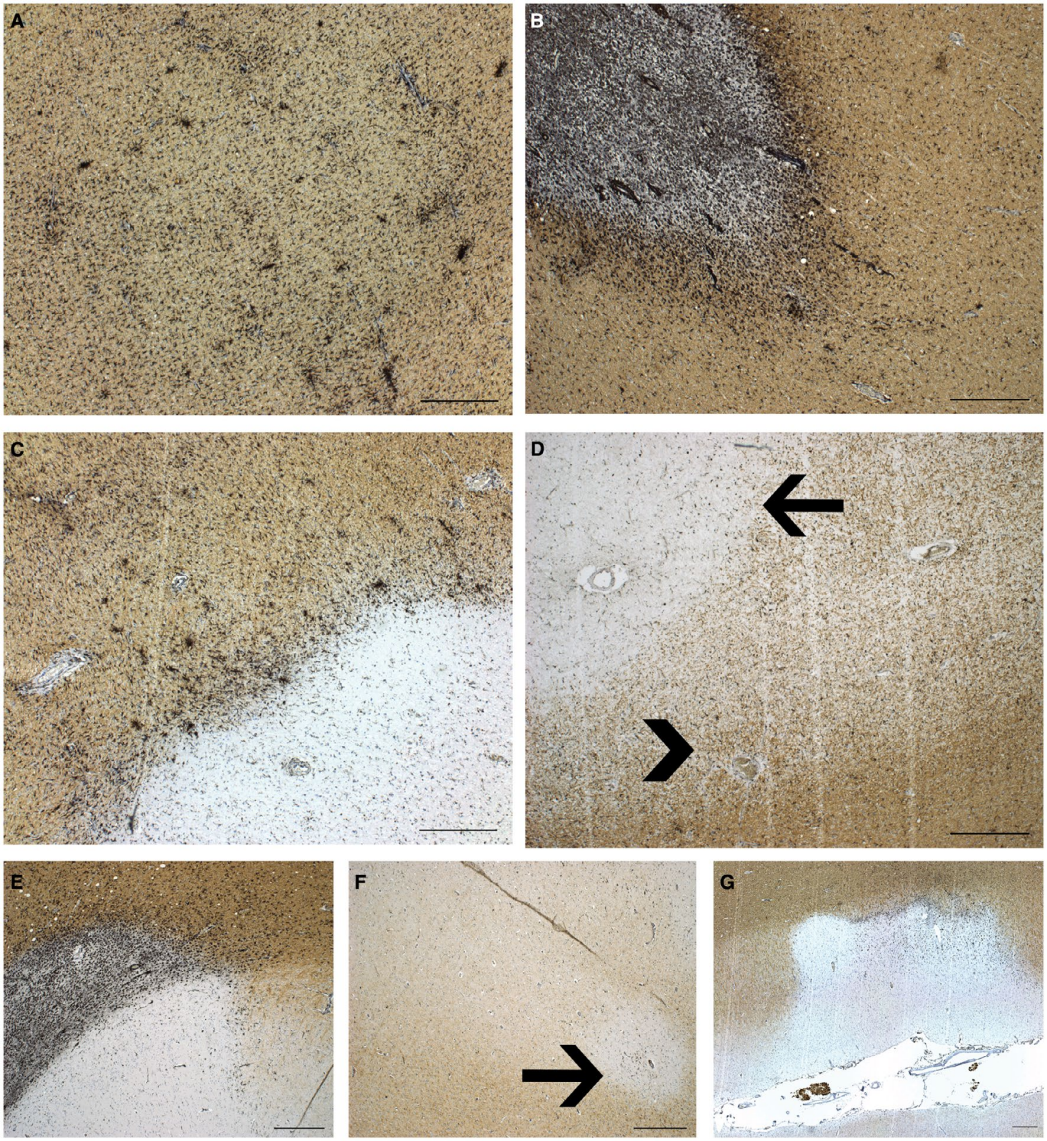
*Pathological characterization of MS lesions*. HLA‐PLP immunohistochemistry of MS autopsy tissue showing the MS lesion characteristics. **A.** Reactive site with increased density of HLA+ cells with microscopically intact myelin. **B.** Active lesion with partial demyelination and HLA+ cells throughout the lesion. **C.** Mixed active/inactive (chronic active) lesion with inactive demyelinated center and a rim of HLA+ cells. **D.** Inactive lesion (arrow), demyelinated with sparse HLA+ cells. Remyelinated lesion (arrow head), partial myelination with sparse HLA+ cells. **E.** Leukocortical lesion. **F.** Intracortical lesion. **G.** Subpial cortical lesion. Scale bar represents 500 um.

The visualization, definition, and calculation of proportion of lesion subtypes is described in Luchetti *et al* 2018 41. In short, double immunostaining was performed on sections from all formalin fixed and paraffin embedded tissues blocks that were dissected from a donor to visualize proteolipid protein (PLP) (MCA839G, AbD Serotec, Oxford, UK, with DAB) and human leukocyte antigen (HLA‐DR‐DQ, referred to as HLA) (M0775, CR3/43, DAKO, Denmark, with DAB‐nickel), as previously described 41. On average, 19.4 ± 12.4 (mean ± SD) tissue blocks were characterized per case. The different lesion characteristics are presented in Figure 1.

### SNP selection

SNPs were selected that were associated with either multiple sclerosis severity score (MSSS), expanded disability status scale (EDSS) or MRI outcomes in three GWAS 4, 6, 7 and one candidate gene SNP study 58. Strong linkage disequilibrium (LD) was defined as r^2^ > 0.8 and strong LD SNPs were identified for the selected SNPs using the SNPsnap database from Broad Institute 45. When two selected SNPs were in strong LD with each other, one of the two SNPs was selected for genotyping (the SNP with highest MAF was selected). SNPs that had a minor allele frequency (MAF) of <0.02 in the GO‐NL database were excluded. In total 69 SNPs were selected in this way. Second, genes were identified that were associated with the MS lesion characteristics in autopsy tissue in micro‐array analyses of laser dissected mixed active/inactive, inactive and remyelinated lesions and normal appearing white matter 33. Furthermore, genes that were previously associated with MS lesion characteristics or related to either microglia/macrophage, T and B cell response or neurodegenerative processes were identified. SNPs in these genes that were either functionally studied or associated with auto‐immune, neurological or psychiatric diseases were identified. In total, 36 SNPs were selected in this way. Table S1 shows the 105 SNPs selected for genotyping and their association with MS with literature references. None of the 105 selected SNPs were in strong LD with each other and MAF was >0.02 in the GO‐NL database.

### Genotyping

DNA was isolated from whole blood or frozen cerebellar tissue. DNA of nine MS cases was isolated from formalin‐fixed paraffin cerebellar tissue using DNeasy Blood & Tissue Kit Qiagen.

Genotyping was performed by LGC genomics (Teddington, UK) using PCR‐based KASP^TM^ genotyping assays. Fluorescence‐based competitive allele‐specific assays were used (www.lgcgroup.com/genomics). Fifty‐one assays were ready‐made by LGC and 54 assays were custom‐made based on 120 base pair sequences flanking the location of the SNP based on hg19 reference genome with annotation of all SNPs with a MAF of >0.1 to prevent primer design at these sites. All SNPs were in Hardy–Weinberg equilibrium (HWE) and MAF in the study population is comparable to the GO‐NL reference population. MAF and HWE are shown in Table S2.

### Linkage disequilibrium and GTEx single tissue eQTL analysis

SNPs in strong LD (*r* ^2^ > 0.8) with SNPs that showed a significant association with MS lesion characteristics were determined by searching the SNiPA database (http://snipa.helmholtz-muenchen.de/snipa3/index.php?task=proxy_search) using the hg19 reference genome. The settings were genome assembly “GRCh37,” variant set “1000 Genomes, Phase 3 v5,” population “European,” genome annotation “Ensembl 87.” The rs numbers of these strong LD SNPs are shown in Table S3. Using the GTEx portal (www.gtexportal.org) single tissue *cis* eQTLs were identified for all strong LD SNPs. Single tissue *cis* eQTLs in brain samples and EBV transformed cells (lymphocytes and fibroblasts) were considered relevant to MS pathogenesis 19, 29.

### Quantitative PCR

40 MS cases that had frozen standardly dissected temporal gyri available were used for qPCR analysis of gene expression. Donor characteristics are shown in Table S4. Frozen tissue from standardly dissected superior temporal gyri was sectioned. Sections of 20 µm were made and 10–50 mg of tissue was collected in the cryostat and stored in ice‐cold TRIsure. After addition of chloroform and centrifugation, the aqueous phase was removed and mixed with an equal volume of Isopropanol. Samples were then centrifuged and supernatant was removed from pellet and the pellet was washed twice with 75% ethanol. RNA samples were dissolved in 20 µL of water and RNA yield was determined using a NanoDrop ND‐1000 spectrophotometer (NanoDrop Technologies, Wilmington, DE, USA). Reverse transcription was performed in a reaction mixture of 10 µL containing 200 ng RNA and gDNAse Wipeout Buffer, incubated for 2 minutes at 42°C and a mixture of Quantiscript Reverse Transcriptase, Quantiscript Buffer and RT‐Primer Mix (Qiagen Benelux, Venlo, The Netherlands), incubated for 30 minutes at 42°C. RT transcriptase was inactivated by incubation for 3 minutes at 95°C. Primers were designed using the primer designer from Integrated DNA Technologies, Inc. NCBI blast was performed for suitable primer sequences using OligoAnalyzer 3.1 (Integrated DNA Technologies, Illinois, USA). Primer sequences and characteristics are shown in Table S5. Specificity was tested on cDNA derived from pooled RNA of both brain and spleen from MS and control donors. Dissociation curves were examined and the PCR product sizes were determined by 8% sodium dodecyl sulfate polyacrylamide gel electrophoresis. Quantitative PCR was performed with SYBR Green PCR Master Mix (Applied Biosystems, Foster City, CA, USA) with samples containing equal cDNA concentrations of 10 ng/µL resulting from 2 ng total RNA per reaction. Analysis was performed according to the manufacturer's protocol at the ABI Prism 7300 Sequence Detection System (Applied Biosystems, Foster City, CA, USA). Target genes were normalized to the geometric mean of glyceraldehyde 3‐phosphate dehydrogenase (GAPDH) and elongation factor 1 alpha (EEF1A1) mRNA expression. qPCR experiments are performed in duplicate.

### Characterization of tissue used for RNA isolation and relative expression analysis

Frozen sections (20 µm) from the standardly dissected superior temporal gyri used for qPCR analysis were fixed for 30 minutes in 4% paraformaldehyde. Sections were incubated with 3% H2O2 solution for 20 minutes to quench endogenous peroxidase activity. Then sections were incubated with 10% normal horse serum for 30 minutes to block non‐specific binding of secondary antibodies. HLA‐PLP double immunohistochemistry was performed and the tissue was characterized as described above and in more detail in Luchetti *et al* 2018 41.

### FAS expression in proteomics data from peripheral T cells

Peripheral lymphocyte mass spectrometry values for FAS in different subpopulations of peripheral T cells were extracted from www.immprot.org. Data are shown in Table S6 52.

### Immunohistochemistry and immunofluorescence

Immunohistochemistry and immunofluorescence were performed for FAS (mouse B‐10 Santa Cruz or HPA027444), CD4 (ab133616, Abcam) and NOGO‐A (11C7, gift from prof. M. Schwab, Zurich 9) on formaldehyde fixed and paraffin‐embedded sections (8 µm) containing mixed active/inactive and active MS lesions. Sections were deparaffinized with xylene and rehydrated. Antigen retrieval was performed using citrate buffer at pH 6 (microwave, 10 minutes at 700 W). Endogenous peroxidase activity and aspecific binding of secondary antibodies was blocked as described above. For fluorescent imaging, sections were incubated with primary antibodies overnight at 4°C. CD4, NOGO‐A and FAS rabbit antibody were visualized using directly labeled secondary antibodies with Alexa 488 fluorophore or Cy3 fluorophore. FAS antibody (B‐10) was visualized by incubation with an appropriate biotinylated secondary antibody followed by incubation with streptavidin‐labeled Cy3 antibody for 45 min. All sections were finally incubated with Hoechst for 10 min. Z stack images were taken using a Leica SP8 confocal microscope and Leica Application Suite X (2017) at 63× magnification. Negative controls with the omission of primary antibody were included.

### Cell isolation and flow cytometry

Peripheral blood mononuclear cells and subcortical white matter‐derived single cell fractions were isolated after rapid post‐mortem autopsies of NBB brain donors as described previously 56, 57. Donor and sample characteristics are listed in Table S7. Cells were stained with fixable viability dye eFluor 780 (Life Technologies) and the following antibodies: CD3 PE‐Cy5.5, clone SK7, (Invitrogen), CD20 APC, clone L27, CD25 FITC, clone 2A3, CD69 BV395, clone FN50, CD127 PE, clone HIL‐7R‐M21, (BD Biosciences), CD4 BV510, clone RPA‐T4, CD8a BV785, clone RPA‐T8, and FAS PE‐Cy7, clone DX2, (Biolegend), and analyzed on a Fortessa LSR^TM^ cell analyzer (BD Biosciences, San Jose, CA, USA). Data were analyzed with FlowJo software 10.5 (Tree Star, Ashland, OR, USA).

### Statistical analysis of SNP‐pathology relationships

The associations of genotype with MS lesion characteristics were analyzed as follows. For all tests the numbers in each group corresponding to each genotype are given in Table S8.

Lesion load was log‐transformed and analyzed with linear models. Presence of cortical gray matter lesion is a binomial yes/no measure and so was analyzed with binomial generalized linear models (GLM). Lesion classification was treated as a yes/no outcome for each lesion type (Luchetti *et al* 2018) and so yes/no counts for each patient were used in a quasibinomial GLM (quasibinomial models allow for additional variance because of between‐patient variation).

In all models, the pathological characteristic was the dependent variable and genotype or genotype and sex were independent categorical variables. Multiple testing correction was performed over all tests of genotype effect on MS lesion characteristics (a total of 612 tests) using the Benjamini‐Hochberg method. All analyses were carried out in R 49. For linear models, significance was calculated using F‐tests for linear models and likelihood ratio chi‐squared tests for GLMs using R package *car* 22. Graphs of pathological characteristics versus genotype show means and 95% confidence intervals calculated by the LMs and GLMs using R package *effects* 21, except for presence of cortical gray lesions where 95% confidence intervals were calculated using R package *binom* using the “bayes” method.

The correlation of log‐transformed relative gene‐expression levels with genotypes were analyzed using a linear model including the presence of an MS lesion and meninges in the tissue block from which RNA was isolated as factors in the model. From all genotypes with sufficiently large (n > 5) genotyping groups, relative gene‐expression levels of the genes they were previously associated to, were compared. rs11957313/C5orf58 was excluded from analysis as the groups were too small.

## Results

### SNP genotyping of the Netherlands Brain Bank MS cohort

About 179 multiple sclerosis autopsy cases were included in the analysis (Table 1). About 102 genotyping assays were successfully performed, giving results that were in Hardy–Weinberg equilibrium. Two pre‐designed assays failed on all samples (rs1557351, rs12202350 4) and these were excluded from analysis. rs1065761 showed no minor allele carriers and is, therefore, excluded from the analysis. Furthermore, the minor allele frequency (MAF) in the NBB cohort is comparable with the Dutch reference population (Table S2). MAF was above >0.02 for all SNPs.

### Six SNPs were associated with MS lesion characteristics

In order to link genotype to pathological parameters, we analyzed the correlation of allelic distributions for each SNP with the proportion of lesion subtypes that was scored for each donor. As described in Luchetti *et al* 2018, reactive, active, mixed active/inactive, inactive, remyelinated, and cortical demyelination were examined in all autopsy cases as shown in Figure 1. On average, 17.6 (S.D. 10.8) dissected tissue blocks were examined per case and in total 3148 tissue blocks containing 8462 lesions were examined 41. Total lesion load, presence of cortical gray matter lesions and the proportions of lesion subtypes, either active, mixed active/inactive (also known as chronic active), or remyelinated and the microglia/macrophage activity score (as calculated in Luchetti *et al* 2018) were tested for correlation with genotype for each SNP.

**Figure 2:**
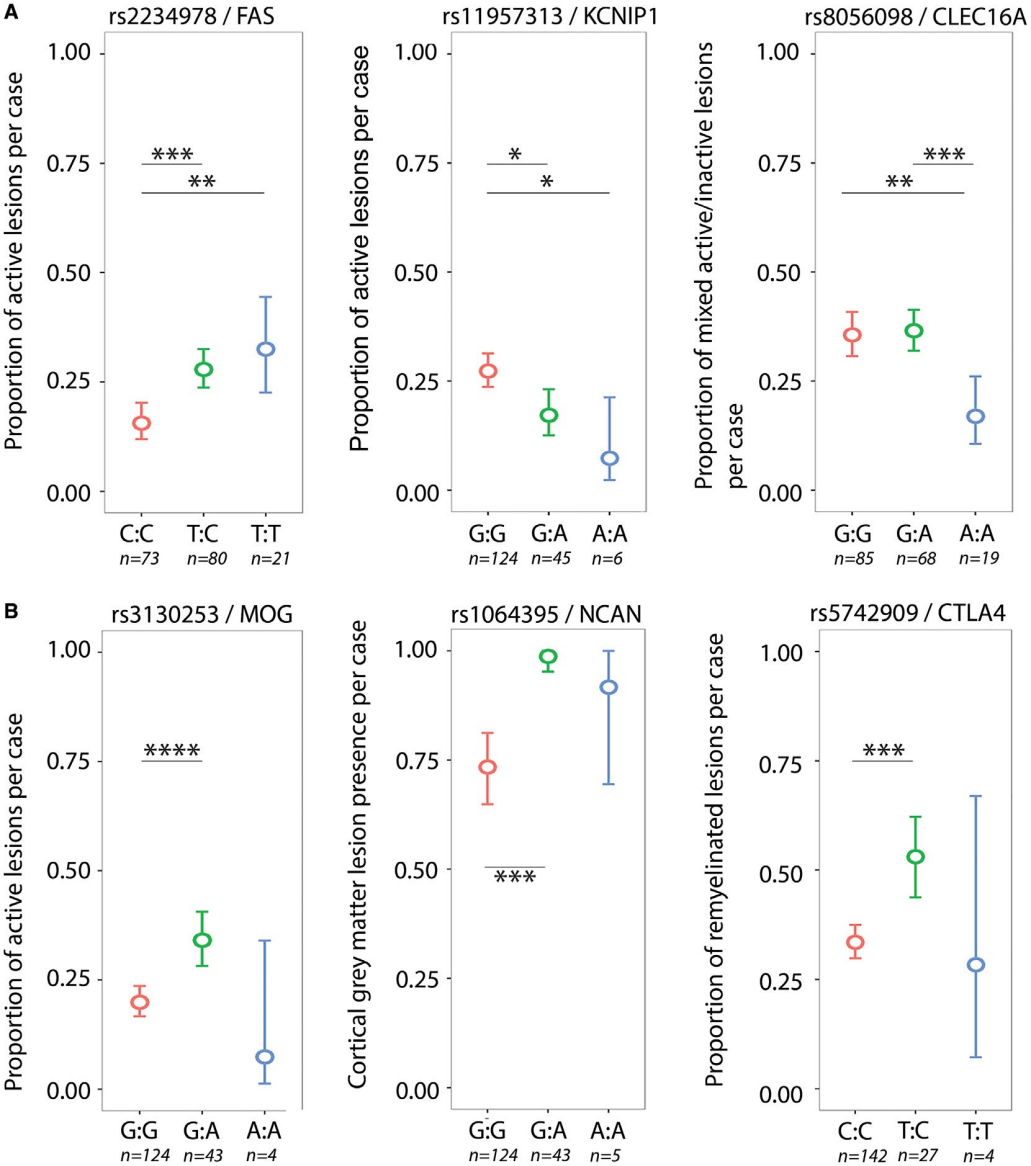
*SNPs significantly associated with MS lesion pathology*. **A.** SNPs previously associated with clinical or MRI measures for disease severity. rs2234978/FAS, heterozygotes (T:C) and minor allele homozygotes (T:T) showed a higher proportion of active lesions compared to major allele homozygotes (C:C). rs11957313/KCNIP1, heterozygotes (G:A) and minor allele homozygotes (A:A) showed a lower proportion of active lesions compared to major allele homozygotes (G:G). rs8056098, minor allele homozygotes (A:A) showed a lower proportion of mixed active/inactive lesions compared to heterozygotes (G:A) and major allele homozygotes (G:G). **B.** SNPs located in genes previously associated with MS pathological characteristics. rs3130253/MOG, heterozygotes (G:A) showed a higher proportion of active lesions compared to major allele homozygotes (G:G). rs1064395/NCAN, heterozygotes (G:A) showed a higher incidence of cortical gray matter lesions compared to major allele homozygotes (G:G). rs5742909/CTLA4, heterozygotes (T:C) showed a higher proportion of remyelinated lesions compared to major allele homozygotes (C:C). *P < 0.05, **P < 0.01, ***P < 0.001, ****P < 0.0001 (generalized linear models).

Three SNPs previously associated with the clinical outcomes in MS showed a significant association with post‐mortem MS lesion characteristics (Figure 2A). Rs2234978/FAS associated with the proportion of active lesions (false discovery rate (FDR) *P* = 0.02). Heterozygotes (T:C) and homozygotes (T:T) for this minor allele had a higher proportion of active lesions compared to major allele homozygotes (C:C). rs11957313/KCNIP1 associated with the proportion of active lesions (FDR *P* = 0.047). Heterozygotes (G:A) and homozygotes (A:A) for this minor allele had a lower proportion of active lesions compared to the major allele homozygotes (G:G). rs8056098/CLEC16A associated with the proportion of mixed active/inactive lesions (FDR *P* = 0.047). Homozygotes for the minor allele (A:A) had a lower proportion of mixed active/inactive lesions compared to homozygotes for the major allele (G:G) and compared to heterozygotes (G:A).

Three SNPs that were located in genes previously associated with MS pathological characteristics showed a significant relation with post‐mortem MS lesion characteristics (Figure 2B). rs1064395/NCAN associated with the incidence of cortical gray matter lesions (FDR *P* = 0.010). Heterozygotes (G:A) had a higher incidence of cortical lesions compared to major allele homozygotes (G:G). rs3130253/MOG associated with the proportion of active lesions (FDR *P* = 0.02). Heterozygotes (G:A) for the minor allele had a higher proportion of active lesions compared to major allele homozygotes (G:G). rs5742909/CTLA4 associated with the proportion of remyelinated lesions (FDR *P* = 0.047). Heterozygotes (C:T) had a higher proportion of remyelinated lesions compared to major allele homozygotes (T:T). Results for the six SNPs showing a significant association are shown in Tables 2 and S8.

**Table 2:** Six SNPs associated with MS lesion characteristics in autopsy tissue.

SNP	Chr:Location (hg19)	Gene	Position (UCSC, hg19)	*P*‐value (FDR)	*P*‐value (post‐hoc test unadjusted)	Pathological outcome	Correlation with MS severity	cis eQTLs GTEx (FDR <0.05)
rs2234978	10:90771829	FAS	exon 7	0.020	CT vs CC (*P* = 0.0003) TT vs CC (*P* = 0.004)	Minor T allele associated with increased proportion of active lesions	Odds ratio 1.83 (TT vs CC) predicting time to EDSS6 58	FAS and ACTA‐2
rs3130253	6:29634012	MOG	exon 3	0.020	AG vs GG (*P* = 0.0001)	Minor A allele associated with increased proportion of active lesions		HLA‐A
rs1064395	19:19361735	NCAN	exon 15	0.010	AG vs GG (*P* = 0.00002 )	Minor A allele associated with increased incidence of cortical gray matter lesions	NCAN significantly upregulated (FC 2.7) in the area around mixed active/inactive lesions compared to the area around inactive lesions 33	CILP2, HAPLN, ZNF101, LPAR2, TSSK6, TM6SF2
rs11957313	5:169950394	KCNIP1	intron 1	0.047	AG vs GG (*P* = 0.015) AA vs GG (*P* = 0.026)	Minor A allele associated with decrease in proportion of active lesions	Brain parenchymal volume (Log *P* > 5.0) 4	C5orf58
rs8056098	16:11138812	CLEC16A	intron 15	0.047	AA vs GG (*P* = 0.002) AA vs GA (*P* = 0.0009)	Homozygotes for minor A allele show a lower proportion of mixed active/inactive lesions	Odds ratio 0.65 (MSSS<2.5 vs >7) 6	NA
rs5742909	2:204732347	CTLA4	near '5	0.047	TC vs CC (*P* = 0.0002)	Minor T allele associated with increase in remyelinated lesions		NA

Of the SNPs with a significant effect on a pathological outcome, sex was not significantly associated with the exception of rs1064395/NCAN versus the incidence of cortical gray matter lesion presence. Their sex was a nominally significant covariate, before multiple testing correction (sex: *P* = 0.02; *χ* ^2^ = 5.4 on 1 d.f.; genotype: *P* = 1.5E‐5; χ^2^ = 22.1 on 2 d.f.). However, genotype was still equally significant in this model. The effect of genotype on cortical gray matter lesions is plotted separately for the sexes (Figure S1).

### Single tissue *cis* eQTLs for significant SNPs

In order to investigate the functional implication of pathology‐associated SNPs, we set out to investigate possible effects of the identified SNPs and SNPs with strong linkage disequilibrium (LD) on transcript abundance, demonstrated by expression quantitative trait loci (eQTL). All SNPs in strong LD (*r* ^2^ > 0.8) with the SNPs found to be significantly associated in the current study were identified and are listed in Table S3. Using the GTEx database 60 single tissue *cis* eQTLs were identified for these SNPs. Only significant eQTLs in brain and EBV‐transformed lymphocytes and fibroblasts were considered relevant to MS pathology 19, 29. rs2234978/FAS showed 216 eQTLs, rs3130253/MOG showed 489 eQTLs, rs1064395/NCAN showed 9088 eQTLs, rs11957313/KCNIP1 showed 1 eQTLs, rs8056098/CLEC16A showed 42 eQTLs, and rs5742909/CTLA4 showed no eQTLs. When considering only the protein coding genes in MS relevant tissues significant associations of genotype with increased or decreased RNA expression levels were found as follows. rs1064395/NCAN is associated with expression levels of six genes (CILP2, HAPLN4, ZNF101, LPAR2, TSSK6, TM6SF2), rs2234978/FAS is associated with two genes (FAS and ACTA2), rs3130253/MOG is associated with one gene (HLA‐A) and rs11957313/KCNIP1 is associated with one gene (C5orf58). rs5742909/CTLA4 and rs8056098/CLEC16A did not show significant eQTLs in the selected tissue types. The eQTL boxplots for the 10 protein coding genes associated with the significant SNPs and their SNPs in strong LD are displayed in Figure S2.

### rs2234978/FAS T allele carriers show an increased FAS RNA expression in brain tissues

**Figure 3:**
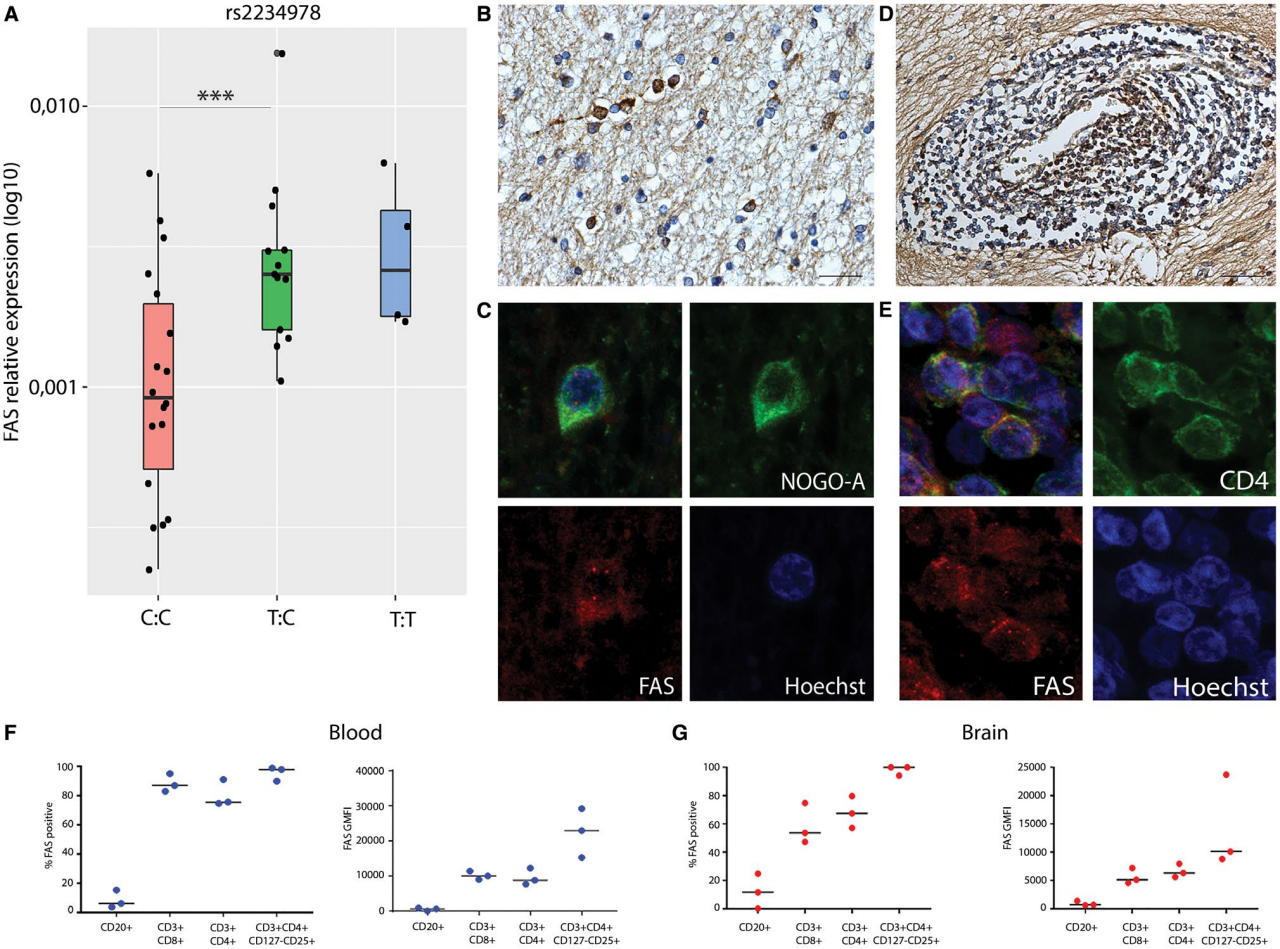
rs2234978/FAS and FAS expression in MS autopsy tissue. **A.** relative expression of FAS with qPCR, heterozygotes showed an increased relative expression of FAS compared to major allele homozygotes C:C n = 22 T:C n = 14 T:T n = 4 ***P < 0.001. **B.** Immunohistochemistry for FAS on active and mixed active/inactive MS lesions showed FAS expression by oligodendrocytes around a mixed active/inactive lesions. (40× magnification, scalebar represents 250 um) **C.** Colocalization of FAS with NOGO‐A, showing it is expressed by oligodendrocytes around MS lesions. (63× magnification) **D.** FAS expression by lymphocytes in the perivascular space. (20× magnification, scalebar represents 500 um) **E.** Colocalization of FAS with CD4, showing it is expressed by T cells in MS lesions. (63× magnification) **F.** FAS protein expression in lymphocytes derived from blood, showing the percentage of FAS positive cells and geometric mean fluorescence intensity (GMFI) for FAS on CD20, CD4, CD8 and CD127‐CD25+CD4+ T cells. FAS is expressed by CD4 and CD8 T cells and suggested to be increased in the CD127‐CD25+ T cell population that is enriched for T‐regulatory phenotype. **G.** FAS protein expression in lymphocytes derived from normal appearing white matter brain tissue, showing the percentage of FAS positive cells and GMFI for FAS on CD20, CD4, CD8 and CD127‐CD25+ CD4+ T cells. FAS is expressed by CD4 and CD8 T cells and suggested to be increased in the few T cells that are enriched for T regulatory phenotype.

To directly link the SNP genotypes to transcript abundance in MS brain tissue, we used qPCR to assess expression levels in standardly dissected superior temporal gyri from 40 MS cases. Gene expression levels were determined for the 10 significant single tissue eQTL genes. rs2234978/FAS showed a consistent effect on FAS gene expression, where heterozygotes for this variant (T:C) showed a higher FAS gene expression level compared to major allele homozygotes (C:C) (*P* = 0.0001) (Figure 3A). Characterization of the tissue blocks showed that all sections contained both white and cortical gray matter. In these 40 standardly dissected sections, 3 white matter lesions and 14 cortical gray matter lesions were present. In 22 cases, meninges were present in the section. Log‐transformed relative expression values were analyzed using a linear regression model including genotype, lesion presence and meninges presence. The presence of a lesion showed no significant effect on FAS gene expression, however, the presence of meninges in the tissue block was a significant factor in the linear model (*P* = 0.005). Therefore, we looked into the effect of the SNP in cases with and cases without meninges in the tissue block. In both groups there was significantly higher FAS expression in carriers of the minor T allele (Figure S3). For the other nine genes we did not find a significant association between genotype and transcript level (Figure S4).

To identify the major source of FAS in and around MS lesions, we performed immunohistochemistry for FAS on mixed active/inactive multiple sclerosis lesions. This showed that it is expressed by oligodendrocytes (Figure 3B) in the NAWM around a mixed active/inactive lesion, as shown by colocalization of FAS with NOGO‐A (Figure 3C). FAS expression is also apparent in lymphocytes in the perivascular space (Figure 3D) as shown by colocalization of FAS with CD4 (Figure 3E). As FAS expression was previously found to be highest in memory regulatory T cells (53, Table S6) we quantified FAS protein expression in freshly isolated lymphocyte populations from white matter brain samples and blood from three non‐MS brain donors using flow‐cytometry (Table S7, Figure S5). In lymphocytes derived from both blood (Figure 3F) and brain (Figure 3G), the percentages of FAS‐positive cells and mean fluorescence intensity of FAS show that it is expressed by both CD4 and CD8 T cells. The highest FAS expression was observed in the rare events of CD4 T cells that were CD127‐ CD25+, supporting high FAS expression in a relatively small population enriched for regulatory T cells.

## Discussion

Here, we translate genotypic information into pathological mechanisms, by analyzing the Netherlands Brain Bank MS autopsy cohort consisting of 179 pathologically characterized MS brain donors in relation to genotyping results for 67 SNPs previously related to the clinical disease course or MRI measures 4, 6, 7, 58 and 35 SNPs located in genes previously related to MS pathological characteristics. This analysis shows that six genetic variants show an effect on post‐mortem MS lesion characteristics. rs2234978/FAS, rs11957313/KCNIP1 and rs3130253/MOG genotypes affected the proportion of active lesions, while rs8056098/CLEC16A genotype had an effect on the proportion of mixed active/inactive lesions. rs1064395/NCAN genotype was associated with the incidence of cortical gray matter lesions and rs5742909/CTLA4 genotype was associated with the proportion of remyelinated lesions. Furthermore, SNP function assessment showed that rs2234978/FAS T allele carriers, which had a higher proportion of active lesions, also showed increased FAS gene expression levels in MS autopsy tissue.

rs2234978/FAS has previously been associated with clinical disease severity 58. We were able to show that the rs2234978 T allele is related to an increased FAS expression in MS brain tissue, which is in line with the single tissue eQTL in brain nucleus accumbens in the GTEx database 60. FAS expression in peri‐lesional oligodendrocytes and T cells, particularly regulatory T cells, suggests two possibilities for the involvement of FAS in MS pathology. The first possible mechanism is that increased FAS expression in oligodendrocytes makes them more likely to undergo apoptosis. FAS is expressed by oligodendrocytes in the area around mixed active/inactive lesions suggesting that carriers of the T allele have a higher FAS expression in oligodendrocytes. FAS functions as an apoptosis receptor for oligodendrocytes 2, 14, 65, therefore, higher FAS expression may make oligodendrocytes more likely to undergo apoptosis and thus more vulnerable to the MS disease process. FAS receptor can be activated by membrane‐bound FAS‐L which is expressed by T cells and in MS lesions by microglia, astrocytes and oligodendrocytes 13, 20, 63. In spinal cord injury, oligodendrocytes undergo FAS‐mediated apoptosis along degenerating axons 10. In mice with traumatic spinal cord injury, blocking of FAS‐mediated apoptosis resulted in reduced oligodendrocyte apoptosis, reduced microglial activation and reduced neuronal and axonal loss and improved functional outcome 1, 11, 18, 54, 67, 68. In the experimental autoimmune encephalomyelitis (EAE) mouse model, it is shown that mice lacking FAS expression on oligodendrocytes are partially protected from EAE with a decrease in demyelination and a mild decrease in infiltration of lymphocytes 34.

The second possible mechanism is that higher FAS expression in T‐cells leads to a more pro‐inflammatory T‐cell population. Immunohistochemistry shows FAS expression by lymphocytes in mixed active/inactive lesions. FAS is mainly expressed by CD4+ T cells and its expression is highest in Treg cells in both blood and brain, in line with previous findings 25, 53. It has been shown that Treg cells are present in MS lesions, with densities from 3 to >15/mm^2^, and are found more often in inactive compared to active MS lesions 24. Treg cells are more vulnerable to FAS mediated apoptosis and exhibit a higher rate of apoptosis compared to other T cell populations ex vivo 24, 25, 46. Interestingly, FAS is also highly expressed by Th17 cells 53, but in contrast to Tregs, activation of the FAS receptor promotes the stability of the Th17 phenotype and prevents their differentiation into Th1 cells 43. Therefore, an increased overall FAS expression in T cells could result in the inhibition of the Treg cells and a more pro‐inflammatory T cell response, leading in turn to a more severe MS disease course 8, 39. This suggests that inhibition of FAS‐mediated apoptosis within the central nervous system (CNS) is a potential target for protection of oligodendrocytes and inhibition of inflammatory disease activity in MS 48.

Rs11957313/KCNIP1 is associated with the proportion of active lesions. Previously, rs11957313 was associated with brain parenchymal volume on MRI, however, the effect on RNA and protein expression remains unknown 4. It is located in intron 1 of the KCNIP1 gene which encodes the potassium voltage gated channel interacting protein 1. The single tissue *cis* eQTL analysis shows an association with C5orf58 expression levels in cortical brain tissues. Unfortunately, as C5orf58 expression in CNS is very low and not detectable with qPCR, we were not able to validate this association.

rs8056098/CLEC16A minor allele was associated with a less severe disease course in the IMSGC GWAS 6, and in our analysis the minor allele is associated with a lower proportion of mixed active/inactive lesions. A higher proportion of mixed active/inactive (chronic active) lesions in autopsy tissue has been repeatedly associated with a more severe and progressive disease course 23, 41. Our findings are consistent with the reported GWAS association 7 and suggests a link between genotype and disease severity via the propensity to form mixed active/inactive lesions. Recently, the function of CLEC16A in the mouse CNS has been described, showing that deficiency of CLEC16A protein impairs autolysosome function and neuronal survival 51. How rs8056098 affects CLEC16A protein level or function in MS patients awaits further analysis. Currently, there are no data supporting a functional link between rs8056098 and CLEC16A expression levels or function.

This analysis shows that rs1064395/NCAN in exon 15 of the gene encoding Neurocan is associated with an increased incidence of cortical lesions in MS brain donors. Microarray analysis of mixed active/inactive and inactive MS lesions revealed NCAN to be specifically upregulated in the area around mixed active/inactive lesions compared to the area around inactive lesions, normal appearing white matter and white matter from healthy controls 33. rs1064395 has previously been found to be associated with susceptibility to bipolar disease and schizophrenia in GWAS studies 12, 44 and with cortical folding in schizophrenia patients 55. In healthy individuals, the minor allele is associated with poorer verbal memory performance 50. The SNP is located in the 3′ untranslated region of NCAN mRNA and so it is well placed to affect translation and localization of this transcript. How this SNP influences NCAN RNA and Neurocan protein expression or function and how it relates to neuronal functions remains to be experimentally determined. The Neurocan protein is involved in the regulation of peri‐neuronal nets around (parvalbumin) neurons 59 and it is shown that peri‐neuronal nets are decreased in MS cortical gray matter lesions compared to normal appearing gray matter without a reduction in the number of neurons 31. This suggests that Neurocan and the stability of peri‐neuronal nets could be associated with cortical demyelination in MS, and could possibly be influenced by the genotype at rs1064395.

Rs3130253/MOG minor allele is associated with an increased proportion of active lesions. rs3130253 is located in exon 3 of the MOG gene and causes a missense mutation resulting in an amino acid change. It has been shown that A allele carriers show relatively increased expression of MOG transcripts that contain exon 2 36. Exon 2 codes for the extra‐cellular, encephalitogenic IgV‐like domain of the MOG protein on oligodendrocytes 16. The immune system may ignore the isoforms that lack exon 2 during development, and thus truncated MOG proteins likely play a role in maintenance of central and peripheral tolerance and/or in inflammatory and demyelinating disease 16, 35, 36. The putative effect of the relative increased presence of MOG exon 2 on auto‐immunity is a promising area for future research.

Finally, we show that rs5742909/CTLA4 is associated with a lower proportion of remyelinated lesions. rs5742909 minor allele is expected to increase the promotor activity of CTLA4, however, it had no effect on CTLA4 transcript levels in brain tissue 61, 64. CTLA4 is a co‐stimulatory molecule that is expressed by activated T cells that may downregulate T cell proliferation and activation during T cell‐dependent immune responses. CTLA4 is abundantly expressed by T cells in human normal white matter 56. In a cuprizone mouse model for demyelination and remyelination, it was shown that the increased infiltration of CD4 T cells impaired spontaneous remyelination 5. These results, therefore, suggest that an increased inhibition of T cell activation relates to a higher proportion of remyelinated MS lesions. Furthermore, CTLA4 is suggested to be an important checkpoint in the development of active MS lesions, as administration of CTLA4 blocking antibodies for treatment of melanoma in an MS patient was linked to the development of MS clinical symptoms and an increase in gadolinium enhancement of MS lesions on MRI 30.

As far as we know this is the most comprehensive study 17, 47, 62, 66 analyzing genotypes in relation to MS pathological characteristics. Our results illustrate that extensive and quantitative pathological characterization of autopsy tissue in sufficient numbers of MS brain donors enables the translation of genotypic variation with known effects on clinical outcome into pathological mechanisms, for example lesion activity. It is unlikely that these SNPs influence the incidence of gadolinium‐enhancing lesions on MRI, as these are known to decline over time 37, 69, while actual lesion activity remains high even over long disease durations 23, 41, indicating a separate pathogenic mechanism for lesion activity in MS cases with long disease duration.

Analyzing the RNA and protein expression associated with the identified variants in different cell populations has great potential to lead to discoveries of targets for disease modifiers 3, 28. Modeling the SNPs in relevant human cells, for example, stem cell‐derived neurons or oligodendrocytes using gene editing tools like CRISPR‐CAS9 will be of interest to elucidate the functional effect of these SNPs. Furthermore, RNA sequencing of isolated nuclei from frozen autopsy tissue is a promising approach to study the transcriptome of human cell‐populations from the central nervous system of brain donors and its association with genotype 38. Improving our understanding of the molecular mechanisms that underlie differences in clinical course will help us to identify biomarkers to improve the prognosis and development of precise therapies in MS patients.

## Authors Contributions

*NLF, JBA, JS, MRM, CGvE, JH, MRJM, IH* designed the study *NLF, CGvE, SL, IH* performed histological characterization of MS lesions, *NLF* analysis of single tissue eQTLs in GTEx data, qPCR, immunohistochemistry and immunofluorescent experiments, drafting manuscript *JS, EBMR, JH, NLF*, performed FACS experiment *MRJM* performed statistical analysis of genotyping data *All authors* contributed to writing the manuscript.

## Competing interest

The authors declare that they have no competing interests.

### Data Availability Statement

The data that support the findings of this study are available from the corresponding author upon reasonable request. The data used for the analyses described in this manuscript were obtained from: GTEx Analysis Release V7 (dbGaP Accession phs000424.v7.p2) the GTEx Portal on 30‐5‐2018. The Genotype‐Tissue Expression (GTEx) Project was supported by the Common Fund of the Office of the Director of the National Institutes of Health, and by NCI, NHGRI, NHLBI, NIDA, NIMH, and NINDS.

## Supporting information


**Table S1.** SNP selection: list of all SNPs that were selected for genotyping (PDF).

**Table S2.** SNP alleles, genotype numbers, MAF and Hardy–Weinberg equilibrium: results for Hardy–Weinberg equilibrium analysis (PDF).

**Table S3.** SNPs in strong LD with pathology associated SNPs: List of all SNPs in strong LD (*r* > 0.8) with the SNPs that showed a significant association with MS lesion characteristics (PDF).

**Table S4.** Donor characteristics: 40 MS cases that were included in qPCR analysis (PDF).

**Table S5.** Primer sequences and characteristics used for qPCR analysis (PDF).

**Table S6.** FAS in peripheral lymphocyte populations (PDF).

**Table S7.** Donor and tissue characteristics: cases included in FACS analysis (PDF).

**Table S8.** Results for all statistical tests performed (PDF).

**Figure S1.** rs1064395 in males and females (PDF).

**Figure S2.** Single tissue eQTLs of significant SNPs and their SNPs in strong LD in brain tissues and EBV transformed cells (lymphocytes and fibroblasts) (PDF).

**Figure S3.** rs2234978 and relative gene expression for FAS in subgroups (PDF).

**Figure S4.** Relative gene expression levels of eQTL genes that were not significantly related to the MS pathology‐associated SNPs (PDF).

**Figure S5.** Flow‐cytometric analysis of lymphocytes derived from peripheral blood and brain (PDF).
