# Supplementary material for: Post‐mortem multiple sclerosis lesion pathology is influenced by single nucleotide polymorphisms
Source: Brain Pathol. 2019 Jul 23;30(1):106–19. doi: 10.1111/bpa.12760 (PMC6916567; doi:10.1111/bpa.12760)
Supplement: Supplementary file 1 — Table S1. SNP selection: list of all SNPs that were selected for genotyping (PDF). [file BPA-30-106-s001.docx]

| **SNP ID** | **Chr:Locatio n (HG19)** | **Gene**  **(nearest within 1 Mbp)** | **Position (UCSC)** | **minor allele** | **reference allele** | **MAF GO- NL** | **categorie** | **Correlation with MS severity** | **SNP function** | **Refs** |
| --- | --- | --- | --- | --- | --- | --- | --- | --- | --- | --- |
| rs17505688 | 1:1076990  41 | NTNG1 | Intron 2 | C | T | C: 0.073 | severity | Odds ratio 2.24 (MSSS<2.5  vs>7) | unknown | PMID:21654844 |
| rs3014866 | 1:1533290  71 | S100A9 | Near 5' | T | C | T: 0.478 | MS  pathology gene | increased in NAWM of  mild MS cases compared to severe (FC 2.6 p=4e-2) | associated with increased  expression of S100A9 in muscle (4 fold, p=0.03) | Melief et.al. under  review PMID: 26795201 |
| rs1061170 | 1:1966592  37 | CFH | Exon 9 | C | T | C: 0.358 | MS  pathology gene | serum factor H higher in progressive compared to relapsing patients (p<0.001) | unknown associated with age-  related macular degeneration (p<0.001), increased vitreous GM-CSF expression (2 fold, p<0.05), choroid CD68 immunoreactivity (p<0.05) | PMID: 20421219  PMID: 25814824 |
| rs1065761 | 1:2031860  93 | CHIT1 | Exon 12 | C | G | C: 0.126 | MS  pathology gene | CHIT1 increased in chronic active vs inactive rim (FC 10.2) | unknown; associated with  atopy (p=0.01, odds 1.32), higher blood eosinophils (P =  .001), total serum IgE (P = .007), and eosinophil cationic protein  (P = .02) levels | PMID: 29312322  PMID: 23706714 |
| rs2796267 | 1:2079249  06 | CD46 | Near 5' | A | G | G: 0.422 | MS  pathology gene | increased in NAWM of severe MS versus mild MS | associated with renal allograft rejection (OR=0.47, p=0.012) | PMID: 21177319 |
| rs2236851 | 1:2524355  4 | RUNX3 | Intron 3 | T | C | T: 0.175 | MS  pathology gene | Upregulated in  remyelinated lesions versus inactive lesion (Luchetti et.al. in prep) | unknown; associated with ulcerative colitis | PMID: 18668679  PMID: 20392673 |
| rs12127450 | 1:4906701  4 | AGBL4 | Intron 9 | C | T | C: 0.160 | severity | Odds ratio 0.53 (MSSS <2.5  vs >7) | unknown | PMID:21654844 |
| rs17541777 | 1:7137550  0 | PTGER3 | Intron 4 | C | T | C: 0.152 | severity | Odds ratio 0.64 (MSSS <5  vs >5) | unknown | PMID:21654844 |
| rs5673 | 1:7147784  1 | PTGER3 | Intron 2 | T | A | T: 0.080 | severity | Odds ratio 2.02 (MSSS <2.5  vs >7) | unknown | PMID:21654844 |
| rs423904 | 2:1138872  62 | IL1RN | Intron 7 | T | C | T: 0.269 | severity | 0.60 (CT/TT vs CC,  predicting MSSS<2.5) |  | PMID: 20378664 |
| rs3814022 | 2:1350479  19 | MGAT5 | Intron 2 | G | C | G: 0.229 | severity | MSSS (p=2.81e-6, CC more  severe then GG and CG) | unknown | PMID: 20117844 |
| rs4953911 | 2:1350687  94 | MGAT5 | Intron 2 | T | A | T: 0.256 | severity | MSSS (p=1.54e-7, TT more  severe then AA and AT) | unknown | PMID: 20117844 |
| rs13387792 | 2:1912219 | MYT1L | Intron 13 | A | G | A: 0.076 | severity | Odds ratio 0.45 (MSSS <2.5  vs>7) | unknown | PMID:21654844 |
| rs2037815 | 2:2021017  15 | CASP8 | Intron 2 | G | A | G: 0.461 | MS  pathology gene | GG genotype associated  with primary progressive MS (OR 1.6, p=0.017) | unknown | PMID: 20363033 |
| rs3116496 | 2:2045945  12 | CD28 | Intron 3 | C | T | C: 0.175 | MS  pathology gene |  | T allele associated with  increased sCD28 levels in RA patients (p=0.047) | PMID: 12864988  PMID: 21508506 |
| rs5742909 | 2:2047323  47 | CTLA4 | Near 5' | T | C | T: 0.076 | MS  pathology gene |  | T allele associated with  increased promotor activity of CTLA-4 (8.13 +/- 0.46 vs 6.87 +/-  0.49) | PMID: 12058260  PMID: 12864988 |
| rs231775 | 2:2047327  14 | CTLA4 | exon 1 | G | A | G: 0.391 | MS  pathology gene |  | G allele associated with less  CTLA-4 expression on stimulated T cells (p=0.019) | PMID: 11086105  PMID: 12864988 |
| rs1597944 | 2:2345040  98 | CHRND | non coding region | C | T | C: 0.442 | severity | T2 lesion load (Log p>5.0) | unknown | PMID: 19010793 |
| rs10178552 | 2:2597330  9 | ASXL2 | Intron 10 | T | C | T: 0.344 | severity | Odds ratio 0.58 (MSSS <2.5  vs >7) | unknown | PMID:21654844 |
| rs17398267 | 2:4893782  3 | LHCGR | Intron 7 | G | T | G: 0.271 | severity | Odds ratio 0.57 (MSSS <2.5  vs >7) | unknown | PMID:21654844 |
| rs13019537 | 2:4897657  7 | LHCGR | Intron 1 | G | C | G: 0.182 | severity | Odds ratio 0.61 (MSSS <5  vs >5) | unknown | PMID:21654844 |
| rs1869410 | 2:5257356 | SOX11 | non coding region | C | T | C: 0.267 | severity | Brain parenchymal volume (Log p>5.0) | unknown | PMID: 19010793 |
| rs13067869 | 3:1736656  77 | NLGN1 | Intron 4 | G | T | G: 0.085 | severity | Brain parenchymal volume  (Log p>5.0) | unknown | PMID: 19010793 |
| rs1799987 | 3:4641193  5 | CCR5 | Intron 1 | G | A | G: 0.481 | MS  pathology gene | G allele associated with  reduced T2 hypertinense (p=0.006) and T1  hypointense lesion volumes on MRI (p=0.036), and A allele associated with early age of onset (-  2.3 years) | A allele associated with higher reporter gene activity (p=0.05) | PMID: 17884183  PMID: 9742978 |
| rs333 | 3:4641494  7-  46414978 | CCR5 | Exon 3 | - | ACAGTCAGT ATCAATTCTG GAAGAATTT CCAG | NA | MS  pathology gene | Deletion was associated with reduced T2 lesion volume (p=0.014), lower black hole ratio on MRI (p=0.010), higher percentage of lesions with signs of remyelination (38% higher, p=0.001) | deletion results in truncated protein that is retained in the endoplasmatic reticulum instead of being expressed on the cell surface | PMID: 17884183  PMID: 9388191 |
| rs11719646 | 3:5646769  1 | ERC2 | Intron 2 | G | A | G: 0.375 | severity | Brain parenchymal volume  (Log p>5.0) | unknown | PMID: 19010793 |
| rs10516537 | 4:1076280  71 | DKK2 | non coding region | A | C | A: 0.151 | severity | MSSS (Log p>5.0) | unknown | PMID: 19010793 |
| rs2069762 | 4:1233779  80 | IL2 | Near 3' | C | A | C: 0.266 | severity | 0.39 (GT vs GG, predicting  MSSS <2.5) |  | PMID: 20378664 |

**Supplementary table 1** SNP selection

List of all SNPs that were selected for genotyping including a description of the correlation of the gene or SNP with multiple sclerosis with the literature reference. SNPs are ordered on chromosome location

| rs12644284 | 4:1541540  00 | TRIM2 | Intron 1 | G | A | G: 0.295 | severity | Odds ratio 0.69 (MSSS <5  vs >5) | unknown | PMID:21654844 |
| --- | --- | --- | --- | --- | --- | --- | --- | --- | --- | --- |
| rs8192678 | 4:2381566  2 | PPARGC1A | Exon 8 | T | C | T: 0.339 | severity | Odds ratio 1.72 (MSSS <2.5  vs >7) | unknown | PMID:21654844 |
| rs305124 | 4:3976958  6 | UBE2K | Intron 4 | G | A | G: 0.063 | severity | T2 lesion load (Log p>5.0) | unknown | PMID: 19010793 |
| rs2853744 | 4:8889624  8 | SPP1 | non coding region | T | G | T: 0.045 | severity | 1.78 (GT vs GG predicting time to EDSS6) |  | PMID: 20378664 |
| rs11750073 | 5:1154805  0 | CTNND2 | Intron 2 | T | C | T: 0.185 | severity | Odds ratio 1.99 (MSSS <2.5  vs >7) | unknown | PMID:21654844 |
| rs2569190 | 5:1400129  16 | CD14 | Exon 1 | A | G | A: 0.483 | MS  pathology gene |  | A allele associated with  increased sCD14 levels in serum (p<0.0001) | PMID: 29515128 |
| rs6198 | 5:1426576  21 | NR3C1 | Exon 9 | C | T | C: 0.206 | MS  pathology gene | haplotype combination  with rs10052957 and rs6189/rs6190 associated with more aggressive disease course (HR 2.3, p<0.001) |  | PMID: 19318444 |
| rs41423247 | 5:1427785  75 | NR3C1 | Intron 2 | C | G | C: 0.381 | MS  pathology gene | no association with severity and MRI in MS | G allele carriers associated with  hyersensitivity to glucocorticoids in dexamethason supression test (p=0.01) | PMID: 19318444  PMID: 14616881 |
| rs6190 | 5:1427803  37 | NR3C1 | Exon 2 | T | C | T: 0.022 | MS  pathology gene | haplotype combination  with rs10052957 and rs6189/rs6190 associated with more aggressive disease course (HR 2.3, p<0.001) | T allele carriers more resistant to the effects of glucocorticoids in dexamethason supression tests | PMID: 19318444  PMID: 12351458 |
| rs10052957 | 5:1427867  01 | NR3C1 | Intron 1 | A | G | A: 0.365 | MS  pathology gene | haplotype combination  with rs10052957 and rs6189/rs6190 associated with more aggressive disease course (HR 2.3, p<0.001) | G allele associated with a lower expression of GR1B mRNA (p=0.005) in middle frontal gryus samples | PMID: 19318444  PMID: 22427805 |
| rs3212227 | 5:1587429  50 | IL12B | Exon 8 | G | T | G: 0.164 | MS  pathology gene | G allele decreased susceptibility to MS (OR  0.20 GG vs A carriers) | G allele associated with reduced production of IL12B | PMID: 11506417  PMID: 11175794 |
| rs11957313 | 5:1699503  94 | KCNIP1 | Intron 1 | A | G | A: 0.156 | severity | Brain parenchymal volume  (Log p>5.0) | unknown | PMID: 19010793 |
| rs10078091 | 5:2549500  5 | CDH10 | Near 3' | A | G | A: 0.293 | severity | Brain parenchymal volume  (Log p>5.0) | unknown | PMID: 19010793 |
| rs9480865 | 6:1089165  73 | FOXO3 | Intron 2 | C | T | C: 0.165 | severity | Brain parenchymal volume  (Log p>5.0) | unknown | PMID: 19010793 |
| rs263153 | 6:1429493  09 | LOC153910 | Intron 1 | T | G | T: 0.083 | severity | T2 lesion load (Log p>5.0) | unknown | PMID: 19010793 |
| rs6941421 | 6:1508915  1 | JARID2 | Near 5' | C | T | C: 0.383 | severity | MSSS (Log p>5.0) | unknown | PMID: 19010793 |
| rs7744583 | 6:1571975  15 | ARID1B | Intron 3 | A | G | A: 0.372 | severity | Odds ratio 1.59 (MSSS <2.5  vs >7) | unknown | PMID:21654844 |
| rs12202350 | 6:1603790  96 | IGF2R | Near 5' | C | T | C: 0.093 | severity | T2 lesion load (Log p>5.0) | unknown | PMID: 19010793 |
| rs6917747 | 6:1604027  05 | IGF2R | Intron 1 | A | G | A: 0.127 | severity | T2 lesion load (Log p>5.0) | unknown | PMID: 19010793 |
| rs3130253 | 6:2963401  2 | MOG | Exon 3 | A | G | A: 0.083 | MS  pathology gene |  | A allele associated with 1.7 fold  overexpression of splice junction for exon 2 to 3 in MOG gene | PMID: 20800907 |
| rs72928038 | 6:9097676  8 | BACH2 | Intron 2 | A | G | A: 0.173 | MS  pathology gene |  | unknown; SNP associated with  Rheumatoid Arthritis susceptibility in meta-analysis (p = 1.2 x10(-8), OR 1.12) | PMID: 24022229 |
| rs6899560 | 6:9627553  8 | FUT9 | Near 5' | G | A | G: 0.047 | severity | T2 lesion load (Log p>5.0) | unknown | PMID: 19010793 |
| rs17157903 | 7:1036280  36 | RELN | Intron 1 | T | C | T: 0.136 | severity | Age of onset (Log p>5.0) | unknown | PMID: 19010793 |
| rs868824 | 7:1103919  21 | IMMP2L | Intron 5 | C | T | C: 0.455 | severity | Age of onset (Log p>5.0) | unknown | PMID: 19010793 |
| rs10243024 | 7:1163466  03 | MET | Intron 2 | A | G | A: 0.235 | severity | MSSS (Log p>5.0) | unknown | PMID: 19010793 |
| rs156429 | 7:2330602  0 | GPNMB | Intron 6 | C | T | C: 0.406 | MS  pathology gene | GPNMB is upregulated in  chronic active rim versus the inactive rim (FC 8.0) and in chronic active peri- lesional white matter versus control white matter (FC 7.1) | unknown; SNP associated with PD susceptibility in males (p=0.01, OR=0.67) | PMID: 29312322  PMID: 25528405 |
| rs12111597 | 7:3487000  1 | NPSR1 and NPSR1-AS1 | Intron 5  and Intron 1 | A | G | A: 0.238 | severity | Odds ratio 0.56 (MSSS <2.5  vs >7) | unknown | PMID:21654844 |
| rs11765693 | 7:7598537  3 | YWHAG | Intron 1 | G | A | G: 0.266 | severity | Odds ratio 1.37 (MSSS <5  vs >5) | unknown | PMID:21654844 |

| rs1761667 | 7:8024493  9 | CD36 | Intron 4 | G | A | G: 0.473 | MS  pathology gene |  | G allele associated with  increased serum Free Fatty Acids (p=0.02) and increased cardiovascular risk (relative risk 1.6, p=0.015), G allele associated with increased CD36 protein expression on monocytes (beta 0.68, p=3.5e- 04) | PMID: 15282206  PMID: 20935172 |
| --- | --- | --- | --- | --- | --- | --- | --- | --- | --- | --- |
| rs10505082 | 8:1067806  28 | ZFPM2 | Intron 5 | A | G | A: 0.163 | severity | Odds ratio 0.58 (MSSS<2.5  vs>7) | unknown | PMID:21654844 |
| rs6994992 | 8:3149558  1 | NRG1 | Near 5' | T | C | T: 0.419 | MS  pathology gene | T allele associated with SP (p=0.038, OR=2.6) and PP MS (p=0.0001, OR=0.04) in  Iranian population | unknown | PMID: 25802071 |
| rs2116078 | 8:7336398  9 | KCNB2 | Near 5' | T | G | T: 0.462 | severity | Age of onset (Log p>5.00) | unknown | PMID: 19010793 |
| rs4880213 | 9:1400310  01 | GRIN1 | Near 5' | T | C | T: 0.389 | MS  pathology gene |  | unknown: C allele associated  with reduced NMDAR-mediated cortical excitability (F=18.04, p<0.01) and disability progression (MSFC) was more frequent in CC vs CT/TT (35% vs 19.3%, p=0.001) | PMID: 23840674 |
| rs16925027 | 9:6978321 | KDM4C | Intron 8 | G | A | G: 0.219 | severity | MSSS (Log p>5.0) | unknown | PMID: 19010793 |
| rs2803418 | 9:7890927  4 | PCSK5 | Intron 25 | T | G | T: 0.245 | severity | Age of onset (Log p>5.0) | unknown | PMID: 19010793 |
| rs10977017 | 9:8380546 | PTPRD | Intron 37 | A | G | A: 0.172 | severity | Odds ratio 1.67 (MSSS <5  or >5) | unknown | PMID:21654844 |
| rs716595 | 10:112006  486 | MXI1 | Intron 3 | A | G | A: 0.078 | severity | Brain parenchymal volume  (Log p>5.0) | unknown | PMID: 19010793 |
| rs2399849 | 10:124998  60 | CAMK1D | Intron 1 | A | G | A: 0.176 | severity | Odds ratio 1.58 (MSSS <5  or >5) | unknown | PMID:21654844 |
| rs2766051 | 10:129121  892 | DOCK1 | Intron 29 | A | G | A: 0.131 | severity | Odds ratio 1.68 (MSSS <5  or >5) | unknown | PMID:21654844 |
| rs7914524 | 10:130104  867 | AK124226 | Exon 4 | T | C | T: 0.212 | severity | Age of onset (Log p>5.0) | unknown | PMID: 19010793 |
| rs1927457 | 10:300086  63 | SVIL | Intron 1 | C | T | C: 0.315 | severity | Brain parenchymal volume  (Log p>5.0) | unknown | PMID: 19010793 |
| rs4747075 | 10:724494  19 | ADAMTS14 | Intron 2 | A | G | A: 0.353 | severity | 0.59 (AA vs GG predicting  time to EDSS6) | unknown | PMID: 20378664 |
| rs1800682 | 10:907499  63 | FAS and ACTA 2 | Intron 2 and Intron 1 | G | A | G: 0.470 | MS  pathology gene | G allele decreased  susceptibility to MS (OR  0.65 G allele carriers vs non carriers) | unknown | PMID: 12098516 |
| rs2234978 | 10:907718  29 | FAS | Exon 7 | T | C | T: 0.331 | severity | 1.83 (TT vs CC predicting time to EDSS6) | SNP creates miR-651 functional  binding site in lung cancer cell line | PMID: 20378664  PMID: 23378343 |
| rs1386330 | 11:878194  27 | RAB38 | Near 3' | C | T | C: 0.132 | severity | Age of onset (Log p>5.0) | unknown | PMID: 19010793 |
| rs7134248 | 12:121897  052 | KDM2B | Intron 1 | T | C | T: 0.442 | severity | Odds ratio 0.73 (MSSS<5  or >5) | unknown | PMID:21654844 |
| rs261902 | 12:324767  27 | BICD1 | Intron 4 | A | G | A: 0.165 | severity | Brain parenchymal volume  (Log p>5.0) | unknown | PMID: 19010793 |
| rs2069727 | 12:685482  23 | IFNG | Near 3' | C | T | C: 0.497 | MS  pathology gene | C allele negatively  associated with MS susceptibility in men (p<0.001) | C allele carriers higher IFNG  expression compared to non carriers in peripheral blood mononuclear cells (p=0.04) | PMID:18332247 |
| rs9319189 | 13:866180  98 | SLITRK6 | Near 5' | A | G | A: 0.330 | severity | Brain parenchymal volume  (Log p>5.0) | unknown | PMID: 19010793 |
| rs79877597 | 14:238449  79 | IL25 | Exon 3 | A | C | A: 0.197 | MS  pathology gene | C allele more common in  severe Psoriasis patients (OR=2.42, p=0.010) in  Spanish cohort. | unknown | PMID: 26347322 |
| rs2039485 | 14:323532  50 | NUBPL | Near 5' | C | T | C: 0.247 | severity | T2 lesion load (Log p>5.0) | unknown | PMID: 19010793 |
| rs752092 | 15:101781  934 | CHSY1 | Intron 1 | A | G | G: 0.317 | severity | MSSS (Log p>5.0) | unknown | PMID: 19010793 |
| rs1448239 | 16:101874  35 | GRIN2A | Intron 2 | C | G | C: 0.164 | severity | Odds ratio 1.89 (MSSS<2.5  vs >7) | unknown | PMID:21654844 |
| rs8056098 | 16:111388  12 | CLEC16A | Intron 15 | A | G | A: 0.396 | severity | Odds ratio 0.65 (MSSS<2.5  vs >7) | unknown | PMID:21654844 |
| rs404694 | 16:795827  98 | MAF | Near 3' | C | A | C: 0.317 | severity | Age of onset (Log p>5.0) | unknown | PMID: 19010793 |
| rs7211577 | 17:141142  80 | COX10 | Near 5' | G | A | G: 0.485 | severity | MSSS (Log p>5.0) | unknown | PMID: 19010793 |
| rs1137933 | 17:261059  32 | NOS2 | Exon 10 | A | G | A: 0.236 | severity | 0.24 (AA vs GG predicting  MSSS <2.5) | unknown | PMID: 20378664 |
| rs9892479 | 17:314219  01 | ASIC2 | Intron 2 | T | G | T: 0.060 | severity | Odds ratio 2.95 (MSSS<2.5  vs >7) | unknown | PMID:21654844 |
| rs1133763 | 17:326478  31 | CCL8 | Exon 3 | C | A | C: 0.157 | MS  pathology gene |  | unknown | PMID: 19415413 |
| rs2107538 | 17:342077  80 | CCL5 | Near 5' | T | C | T: 0.179 | MS  pathology gene | T allele associated with  worse MSSS score (p=0.045) and reduced risk for severe axonal damage (OR 0.84) | T allele associated with increased transcription CCL5 (8 fold higher) | PMID: 10640782  PMID: 12204866  PMID: 17884183 |
| rs11652878 | 17:365498  0 | ITGAE | Exon 15 | G | A | G: 0.105 | MS  pathology gene |  | G allele associated with  decreased susceptibility of Graves Ophthalmopathy (p=0.006) | PMID: 20417566 |

| rs876493 | 17:378245  45 | PNMT | Intron 1 | G | A | G: 0.429 | severity | 0.52 (GG vs AA/AG  predicting MSSS <2.5) | unknown | PMID: 20378664 |
| --- | --- | --- | --- | --- | --- | --- | --- | --- | --- | --- |
| rs744166 | 17:405142  01 | STAT3 | Intron 1 | G | A | G: 0.434 | MS  pathology gene | Associated with MS (OR 0.87, p=2.75 x10(-10)) | unknown | PMID: 20159113 |
| rs1318 | 17:656913  82 | PITPNC1 | Exon 10 | G | A | G: 0.193 | severity | 0.59 (GG vs AA predicting  MSSS<2.5) |  | PMID: 20378664 |
| rs2028455 | 18:479660  05 | SKA1 | Near 3' | T | C | T: 0.316 | severity | MSSS (Log p>5.0) | unknown | PMID: 19010793 |
| rs1557351 | 18:547523  14 | WDR7 | Near 3' | C | T | C: 0.187 | severity | Age of onset (Log p>5.0) | unknown | PMID: 19010793 |
| rs337718 | 18:697742  78 | CBLN2 | Near 5' | T | C | T: 0.283 | severity | MSSS (Log p>5.0) | unknown | PMID: 19010793 |
| rs7253363 | 19:116824  95 | BC039523 | Intron 4 | T | G | T: 0.034 | severity | MSSS (Log p>5.0) | unknown | PMID: 19010793 |
| rs2074897 | 19:139123  5 | NDUFS7 | Intron 7 | A | G | A: 0.485 | severity | 1.44 (AA vs GG, predicting  time to EDSS6) |  | PMID: 20378664 |
| rs11666377 | 19:171184  33 | CPAMD8 | Intron 7 | T | C | T: 0.162 | severity | T2 lesion load (Log p>5.0) | unknown | PMID: 19010793 |
| rs1064395 | 19:193617  35 | NCAN | Exon 15 | A | G | A: 0.145 | MS  pathology gene | NCAN significantly upregulated (FC 2.7) in the area around chronic active lesions compared to the area around inactive lesions | unknown; SNP associated with susceptibility of bipolar disorder and schizophrenia, cortical folding in schizophrenia and cognitive functioning in healthy individuals | PMID: 29312322  PMID: 21353194  PMID: 22497794  PMID: 23795679  PMID: 25220293 |
| rs3865444 | 19:517279  62 | CD33 | Near 5' | A | C | A: 0.308 | MS  pathology gene |  | A allele protective factors for  AD onset p=0.003, A allele greater cell surface expression of CD33 in monocytes, deminished internalization of amyloid. | PMID: 26795201  PMID: 23708142 |
| rs299175 | 19:563135  28 | NLRP11 | Intron 1 | A | G | A: 0.464 | severity | MSSS (Log p>5.0) | unknown | PMID: 19010793 |
| rs1883832 | 20:447469  82 | CD40 | Exon 1 | T | C | T: 0.234 | MS  pathology gene | T allele associated with  increased susceptibility for Multiple Sclerosis (OR 1.175) | unknown | PMID: 29254239 |
| rs9808753 | 21:347873  12 | IFNGR2 | Exon 2 | G | A | G: 0.143 | MS  pathology gene | G allele associated with  progressive onset (p=0.028) and lower black hole ratio on MRI (p=0.016) | unknown | PMID: 15182327 |
| rs4819554 | 22:175650  35 | IL17RA | Near 5' | G | A | G: 0.182 | MS  pathology gene | G allele associated with Psoriasis in Spanish cohort (OR 1.33, p=0.017). A allele  associated with response to TNF alpha in Psoriasis  patients (p=0.03, OR 1.86) | unknown | PMID: 26347322  PMID: 27670766 |
| rs755622 | 22:242363  92 | MIF-AS1 | Exon 3 | C | G | C: 0.192 | MS  pathology gene | C allele associated with a  more severe disease course in males with MS (p  <0.01) | unknown; C allele associated with increased serum MIF level in children (p=0.04) | PMID: 29661540  PMID: 26541175 |
