# Supplementary material for: Post‐mortem multiple sclerosis lesion pathology is influenced by single nucleotide polymorphisms
Source: Brain Pathol. 2019 Jul 23;30(1):106–19. doi: 10.1111/bpa.12760 (PMC6916567; doi:10.1111/bpa.12760)
Supplement: Supplementary file 2 — Table S2. SNP alleles, genotype numbers, MAF and Hardy–Weinberg equilibrium: results for Hardy–Weinberg equilibrium analysis (PDF). [file BPA-30-106-s002.pdf]

Supplementary table 2 SNP alleles, genotype numbers, MAF and Hardy-Weinberg equilibrium: results for Hardy Weinberg equilibrium analysis
Results for Hardy Weinberg equilibrium analysis and reported MAF in study cohort compared to MAF in GO-NL database.

locus	major	minor	homo-zygote major	homo-zygote minor	hetro-zygote	MAF study population	MAF GO-NL	NCBI MAF EUR-population	HWE p	HWE p.adj
rs10052957	G	A	79	22	86	0,348	A: 0.365		0,848	0,947
rs10078091	G	A	95	14	76	0,281	A: 0.293		0,823	0,947
rs10178552	C	T	73	25	87	0,370	T: 0.344		0,909	0,947
rs10243024	G	A	119	9	54	0,198	A: 0.235		0,380	0,903
rs10505082	G	A	123	1	62	0,172	A: 0.163		0,020	0,426
rs10516537	C	A	132	7	45	0,160	A: 0.151		0,214	0,903
rs1061170	T	C	70	24	84	0,371	C: 0.358		0,879	0,947
rs1064395	G	A	132	5	47	0,155	A: 0.145		0,742	0,947
rs1065761	G	None	181	0	0	0,000	C: 0.126		NaN	NA
rs10977017	G	A	124	5	52	0,171	A: 0.172		0,871	0,947
rs1133763	A	C	124	3	58	0,173	C: 0.157		0,193	0,903
rs1137933	G	A	115	8	65	0,215	A: 0.236		0,754	0,947
rs11652878	A	G	160	1	25	0,073	G: 0.105		0,982	0,992
rs11666377	C	T	134	3	52	0,153	T: 0.162		0,417	0,903
rs11719646	A	G	61	23	99	0,396	G: 0.375		0,077	0,785
rs11750073	C	T	122	7	55	0,188	T: 0.185		0,797	0,947
rs11765693	A	G	81	13	87	0,312	G: 0.266		0,108	0,790
rs11957313	G	A	132	6	49	0,163	A: 0.156		0,583	0,947
rs12111597	G	A	99	10	73	0,255	A: 0.238		0,464	0,903
rs12127450	T	C	133	3	47	0,145	C: 0.160		0,617	0,947
rs12644284	A	G	103	13	69	0,257	G: 0.295		0,757	0,947
rs13019537	C	G	126	4	57	0,174	G: 0.182		0,401	0,903
rs13067869	T	G	147	2	30	0,095	G: 0.085		0,737	0,947
rs1318	A	G	117	5	60	0,192	G: 0.193		0,409	0,903
rs13387792	G	A	164	0	23	0,061	A: 0.076		0,370	0,903
rs1386330	T	C	142	1	39	0,113	C: 0.132		0,332	0,903
rs1448239	G	C	130	1	53	0,149	C: 0.164		0,071	0,785
rs156429	T	C	62	32	91	0,419	C: 0.406		0,888	0,947
rs1597944	T	C	56	43	87	0,465	C: 0.442		0,414	0,903
RS16925027	A	G	111	8	62	0,215	G: 0.219		0,859	0,947
rs17157903	C	T	150	3	34	0,107	T: 0.136		0,510	0,903
rs17398267	T	G	112	14	58	0,234	G: 0.271		0,104	0,790
rs17505688	T	C	154	2	26	0,082	C: 0.073		0,454	0,903
rs17541777	T	C	145	2	34	0,105	C: 0.152		0,997	0,997
rs1761667	A	G	58	39	85	0,448	G: 0.473		0,453	0,903
rs1799987	A	G	55	43	88	0,468	G: 0.481		0,497	0,903
rs1800682	T	C	43	41	100	0,495	G: 0.470	C:0.4314	0,237	0,903
rs1869410	T	C	88	19	71	0,306	C: 0.267		0,414	0,903
rs1883832	C	T	109	11	68	0,239	T: 0.234		0,927	0,947
rs1927457	T	C	79	14	84	0,316	C: 0.315		0,196	0,903
rs2028455	C	T	82	19	84	0,330	T: 0.316		0,711	0,947
rs2037815	A	G	46	42	99	0,489	G: 0.461		0,417	0,903
rs2039485	T	C	104	10	74	0,250	C: 0.247		0,496	0,903
rs2069727	A	G	45	43	92	0,494	C: 0.497	C:0.4632	0,764	0,947
rs2069762	T	G	110	11	65	0,234	C: 0.266	G:0,2922	0,735	0,947
rs2074897	G	A	54	33	94	0,442	A: 0.485		0,477	0,903
rs2107538	C	T	119	5	59	0,189	T: 0.179		0,467	0,903
rs2116078	G	T	49	38	93	0,469	T: 0.462		0,618	0,947
rs2234978	C	T	81	22	83	0,341	T: 0.331		0,917	0,947
rs2236851	C	T	126	7	53	0,180	T: 0.175		0,631	0,947
rs231775	A	G	73	21	95	0,362	G: 0.391		0,228	0,903
rs2399849	G	A	134	2	46	0,137	A: 0.176		0,370	0,903
rs2569190	G	A	43	39	96	0,489	A: 0.483		0,291	0,903
rs261902	C	T	114	8	57	0,204	A: 0.165	T:0,1700	0,797	0,947
rs263153	G	T	148	0	37	0,100	T: 0.083		0,131	0,833
rs2766051	G	A	145	4	34	0,115	A: 0.131		0,247	0,903
rs2796267	A	G	54	36	86	0,449	G: 0.422		0,869	0,947
rs2803418	G	T	91	10	80	0,276	T: 0.245		0,156	0,903
RS2853744	G	T	171	2	15	0,051	T: 0.045		0,021	0,426
rs299175	C	T	53	39	87	0,461	A: 0.464	T: 0.4433	0,769	0,947

rs3014866	T	C	54	39	95	0,460	T: 0.478		0,814	0,947
rs305124	A	G	151	1	33	0,095	G: 0.063		0,574	0,947
rs3116496	T	C	134	4	45	0,145	C: 0.175		0,923	0,947
rs3130253	G	A	134	4	45	0,145	A: 0.083		0,923	0,947
rs3212227	A	C	125	7	44	0,165	G: 0.164	C:0.2227	0,224	0,903
rs333	GTCAGTATCAATTCTGGAAGAATTTCCAGACA	-	154	5	26	0,097	NA	-:0,106	0,007	0,222
rs337718	C	T	91	15	78	0,293	T: 0.283		0,763	0,947
rs3814022	C	G	114	6	64	0,207	G: 0.229		0,406	0,903
rs3865444	G	T	86	28	75	0,347	A: 0.308	T: 0.3101	0,089	0,790
rs404694	A	C	92	14	79	0,289	C: 0.317		0,599	0,947
rs41423247	G	C	88	31	65	0,345	C: 0.381		0,003	0,155
rs423904	C	T	91	8	80	0,268	T: 0.269		0,064	0,785
rs4747075	G	A	86	30	58	0,339	A: 0.353		0,001	0,074
rs4819554	A	G	145	4	38	0,123	G: 0.182		0,427	0,903
rs4880213	C	T	63	24	99	0,395	T: 0.389		0,122	0,828
rs4953911	A	T	103	8	74	0,243	T: 0.256		0,239	0,903
rs5673	A	T	169	0	17	0,046	T: 0.080		0,514	0,903
rs5742909	C	T	154	4	27	0,095	T: 0.076		0,044	0,643
rs6190	G	A	166	1	20	0,059	T: 0.022	A:0.02980	0,641	0,947
rs6198	T	C	118	9	54	0,199	C: 0.206		0,391	0,903
rs6899560	A	G	170	0	18	0,048	G: 0.047		0,491	0,903
rs6917747	G	A	137	6	44	0,150	A: 0.127		0,299	0,903
rs6941421	T	C	62	28	95	0,408	C: 0.383		0,392	0,903
rs6994992	C	T	70	28	90	0,388	T: 0.419		0,915	0,947
rs7134248	C	T	54	39	93	0,460	T: 0.442		0,929	0,947
rs716595f	G	A	144	1	41	0,116	A: 0.078		0,287	0,903
rs7211577	G	A	57	34	96	0,439	G: 0.485		0,561	0,947
rs7253363	G	T	180	0	7	0,019	T: 0.034		0,794	0,947
rs72928038	G	A	119	2	67	0,189	A: 0.173		0,025	0,427
rs744166	T	C	63	30	90	0,410	G: 0.434		0,822	0,947
rs752092	T	C	85	24	81	0,339	G: 0.317	C:0.3449	0,496	0,903
rs755622	C	G	119	3	63	0,186	C: 0.192	G:0.1859	0,096	0,790
rs7744583	G	A	78	20	85	0,342	A: 0.372		0,658	0,947
rs7914524	C	T	110	10	62	0,225	T: 0.212		0,746	0,947
rs79877597	C	A	127	6	53	0,175	A: 0.197		0,870	0,947
rs8056098	G	A	75	20	89	0,351	A: 0.396		0,398	0,903
rs8192678	G	A	90	17	77	0,302	T: 0.339	A: 0.3608	0,928	0,947
rs868824	T	C	62	40	81	0,440	C: 0.455		0,169	0,903
rs876493	A	G	71	34	81	0,401	G: 0.429		0,204	0,903
rs9319189	G	A	92	13	76	0,282	A: 0.330		0,615	0,947
rs9480865	T	C	142	5	39	0,132	C: 0.165		0,256	0,903
rs9808753	A	G	134	6	48	0,160	G: 0.143		0,510	0,903
rs9892479	G	T	165	1	22	0,064	T: 0.060		0,775	0,947
