# Supplementary material for: Post‐mortem multiple sclerosis lesion pathology is influenced by single nucleotide polymorphisms
Source: Brain Pathol. 2019 Jul 23;30(1):106–19. doi: 10.1111/bpa.12760 (PMC6916567; doi:10.1111/bpa.12760)
Supplement: Supplementary file 3 — Table S3. SNPs in strong LD with pathology associated SNPs: List of all SNPs in strong LD (r > 0.8) with the SNPs that showed a significant association with MS lesion characteristics (PDF). [file BPA-30-106-s003.docx]

| Query SNP | RSID | RSALIAS | CHR | POS1 | POS2 | DIST | r2 | MAJOR | MINOR | MAF |
| --- | --- | --- | --- | --- | --- | --- | --- | --- | --- | --- |
| rs3130253 | rs3130253 | rs57482224,rs52819569,r  s116988123,rs115538733, rs111678575 | 6 | 29634012 | 29634012 | 0 | 1 | G | A | 0.0884692 |
|  | rs3132731 | rs9258280,rs59927670,rs  117396254,rs115419066,r s111911408 | 6 | 29634012 | 29733374 | 99362 | 0.963141 | C | T | 0.0854871 |
|  | rs3115630 | rs60900792,rs17745719,r  s117957769,rs114871936, rs112391467 | 6 | 29634012 | 29794501 | 160489 | 0.950957 | C | T | 0.0864811 |
|  | rs3132129 | rs59963128,rs117473922,  rs114579793 | 6 | 29634012 | 30027860 | 393848 | 0.855259 | G | A | 0.084493 |
|  | rs9261387 | rs57210406,rs17454240,r  s17187812,rs117683490,r s114678021 | 6 | 29634012 | 30061361 | 427349 | 0.902241 | C | T | 0.0805169 |
| rs2234978 | rs2147419 | rs57517680,rs386558720 | 10 | 90771829 | 90759916 | -11913 | 0.911553 | T | G | 0.282306 |
|  | rs2031613 | rs58544582,rs386553541 | 10 | 90771829 | 90766924 | -4905 | 0.981113 | T | C | 0.299205 |
|  | rs2031611 | rs59358909 | 10 | 90771829 | 90767395 | -4434 | 0.985793 | C | G | 0.298211 |
|  | rs1571020 | rs56797918 | 10 | 90771829 | 90770852 | -977 | 1 | T | C | 0.297217 |
|  | rs2234978 | rs60634379,rs3740283,rs  3218616,rs17850927,rs17  376935 | 10 | 90771829 | 90771829 | 0 | 1 | C | T | 0.297217 |
|  | rs1800623 | rs56832082,rs4097324,rs  1983889 | 10 | 90771829 | 90776083 | 4254 | 1 | G | A | 0.297217 |
|  | rs4934435 | rs58147217,rs57470060,r  s17449490 | 10 | 90771829 | 90776396 | 4567 | 1 | G | A | 0.297217 |
|  | rs1926188 | rs58782347 | 10 | 90771829 | 90777111 | 5282 | 0.995248 | C | T | 0.296223 |
|  | rs7907684 | rs58570558 | 10 | 90771829 | 90777376 | 5547 | 1 | T | C | 0.297217 |
|  | rs11202925 | rs60518911 | 10 | 90771829 | 90777641 | 5812 | 0.990509 | G | A | 0.295229 |
|  | rs11202927 | NA | 10 | 90771829 | 90777850 | 6021 | 0.876236 | T | G | 0.270378 |
|  | rs2011806 | rs57539377 | 10 | 90771829 | 90778635 | 6806 | 1 | C | T | 0.297217 |
|  | rs1810764 | rs58014202 | 10 | 90771829 | 90778656 | 6827 | 0.995248 | C | T | 0.296223 |
|  | rs7092857 | rs58532963,rs56420376,r  s17449588 | 10 | 90771829 | 90779318 | 7489 | 1 | T | C | 0.297217 |
|  | rs10887879 | NA | 10 | 90771829 | 90781976 | 10147 | 0.866076 | G | A | 0.280318 |
|  | rs12257092 | rs59385687 | 10 | 90771829 | 90782827 | 10998 | 0.866076 | A | T | 0.280318 |
| rs1064395 | rs111492783 | NA | 19 | 19361735 | 19357612 | -4123 | 0.943103 | A | G | 0.158052 |
|  | rs112219496 | NA | 19 | 19361735 | 19358086 | -3649 | 0.957177 | G | A | 0.162028 |
|  | rs111444407 | NA | 19 | 19361735 | 19358207 | -3528 | 0.964313 | C | T | 0.161034 |
|  | rs4808932 | NA | 19 | 19361735 | 19358819 | -2916 | 1 | C | T | 0.166004 |
|  | rs8108777 | NA | 19 | 19361735 | 19359058 | -2677 | 1 | C | A | 0.166004 |
|  | rs10423874 | NA | 19 | 19361735 | 19359500 | -2235 | 1 | C | G | 0.166004 |
|  | rs1064395 | rs57422559 | 19 | 19361735 | 19361735 | 0 | 1 | G | A | 0.166004 |
|  | rs10401714 | NA | 19 | 19361735 | 19363630 | 1895 | 1 | G | A | 0.166004 |
|  | rs4808933 | NA | 19 | 19361735 | 19363812 | 2077 | 1 | T | A | 0.166004 |
|  | rs72999030 | rs74254019 | 19 | 19361735 | 19364240 | 2505 | 1 | A | T | 0.166004 |
|  | rs10407933 | NA | 19 | 19361735 | 19364319 | 2584 | 1 | A | G | 0.166004 |
|  | rs17216041 | rs60305265 | 19 | 19361735 | 19366643 | 4908 | 0.950267 | G | A | 0.16501 |
|  | rs56044734 | NA | 19 | 19361735 | 19366927 | 5192 | 0.957384 | G | A | 0.166004 |
|  | rs56278919 | NA | 19 | 19361735 | 19367190 | 5455 | 0.950267 | A | G | 0.16501 |
|  | rs55762233 | NA | 19 | 19361735 | 19367319 | 5584 | 0.957384 | C | G | 0.166004 |
|  | rs55765017 | NA | 19 | 19361735 | 19368264 | 6529 | 0.950267 | G | A | 0.16501 |
|  | rs56144632 | NA | 19 | 19361735 | 19368310 | 6575 | 0.950267 | G | A | 0.16501 |
|  | rs4808934 | NA | 19 | 19361735 | 19370917 | 9182 | 0.97884 | C | T | 0.168986 |
|  | rs56135441 | NA | 19 | 19361735 | 19371370 | 9635 | 0.957384 | A | T | 0.166004 |
|  | rs7257875 | NA | 19 | 19361735 | 19372150 | 10415 | 0.97884 | C | T | 0.168986 |
|  | rs10422893 | NA | 19 | 19361735 | 19372185 | 10450 | 0.97884 | G | C | 0.168986 |
|  | rs10403273 | rs111064614,rs11105443  9 | 19 | 19361735 | 19372968 | 11233 | 0.965144 | C | G | 0.170974 |
|  | rs72999053 | NA | 19 | 19361735 | 19375372 | 13637 | 0.936586 | G | A | 0.166998 |
|  | rs564726571 | NA | 19 | 19361735 | 19376215 | 14480 | 0.81155 | C | T | 0.151093 |
|  | rs200962751 | NA | 19 | 19361735 | 19376220 | 14485 | 0.81155 | C | T | 0.151093 |
|  | rs72999059 | NA | 19 | 19361735 | 19378245 | 16510 | 0.936586 | A | C | 0.166998 |
|  | rs998732 | NA | 19 | 19361735 | 19378671 | 16936 | 0.936586 | A | G | 0.166998 |
|  | rs72999063 | NA | 19 | 19361735 | 19380596 | 18861 | 0.9437 | G | T | 0.167992 |
|  | rs72999068 | NA | 19 | 19361735 | 19380685 | 18950 | 0.936586 | A | G | 0.166998 |
|  | rs2074302 | NA | 19 | 19361735 | 19381728 | 19993 | 0.9437 | G | C | 0.167992 |
|  | rs55822665 | NA | 19 | 19361735 | 19383298 | 21563 | 0.936586 | C | T | 0.166998 |
|  | rs2010506 | rs386552453 | 19 | 19361735 | 19387356 | 25621 | 0.936586 | C | T | 0.166998 |
|  | rs11555053 | rs16994681 | 19 | 19361735 | 19390185 | 28450 | 0.936586 | G | A | 0.166998 |

**Supplementary table 3.** SNPs in strong LD with pathology associated SNPs

List of all SNPs that are in strong LD (r> 0.8) with the SNPs that showed a significant association with MS lesion characteristics. RSID indicates the dbSNP rs-identifier for variants in strong LD. RSALIAS are previous dbSNP rs-identifiers for the strong LD variant. CHR is the chromosome number. POS1 is the position of the query SNP. POS2 is the position of the SNP in strong LD. Distance indicates the distance between query SNP and LD SNP.

|  | rs6511027 | NA | 19 | 19361735 | 19391851 | 30116 | 0.9437 | C | T | 0.167992 |
| --- | --- | --- | --- | --- | --- | --- | --- | --- | --- | --- |
|  | rs8113794 | NA | 19 | 19361735 | 19393653 | 31918 | 0.929868 | A | G | 0.167992 |
|  | rs8100713 | NA | 19 | 19361735 | 19393657 | 31922 | 0.929868 | T | C | 0.167992 |
|  | rs8100097 | NA | 19 | 19361735 | 19393683 | 31948 | 0.915697 | G | A | 0.166004 |
|  | rs56212061 | NA | 19 | 19361735 | 19394640 | 32905 | 0.887264 | C | T | 0.16004 |
|  | rs17751061 | rs57889381 | 19 | 19361735 | 19413092 | 51357 | 0.915347 | C | T | 0.164016 |
|  | rs2315023 | rs57659202 | 19 | 19361735 | 19413393 | 51658 | 0.92949 | C | T | 0.166004 |
|  | rs76944848 | NA | 19 | 19361735 | 19417512 | 55777 | 0.915347 | C | T | 0.164016 |
|  | rs113145184 | NA | 19 | 19361735 | 19419984 | 58249 | 0.92949 | T | C | 0.166004 |
|  | rs112253053 | NA | 19 | 19361735 | 19425145 | 63410 | 0.92949 | T | A | 0.166004 |
|  | rs80146298 | NA | 19 | 19361735 | 19426317 | 64582 | 0.915347 | C | T | 0.164016 |
|  | rs4808944 | NA | 19 | 19361735 | 19426613 | 64878 | 0.92949 | A | C | 0.166004 |
|  | rs57436187 | NA | 19 | 19361735 | 19429915 | 68180 | 0.92949 | C | T | 0.166004 |
|  | rs4239639 | rs57730301 | 19 | 19361735 | 19435876 | 74141 | 0.884099 | A | G | 0.172962 |
|  | rs12972691 | NA | 19 | 19361735 | 19437500 | 75765 | 0.884099 | T | A | 0.172962 |
|  | rs1557723 | rs58740104 | 19 | 19361735 | 19443033 | 81298 | 0.863838 | G | A | 0.171968 |
|  | rs2011503 | rs57843682,rs57575636,r  s56546520,rs17683908 | 19 | 19361735 | 19443986 | 82251 | 0.870866 | C | T | 0.172962 |
|  | rs1989867 | rs59376502 | 19 | 19361735 | 19447470 | 85735 | 0.870866 | G | A | 0.172962 |
|  | rs756999 | rs59350647 | 19 | 19361735 | 19448200 | 86465 | 0.870866 | G | A | 0.172962 |
|  | rs2965190 | rs61268932 | 19 | 19361735 | 19451591 | 89856 | 0.870866 | G | A | 0.172962 |
|  | rs2965194 | rs56836648 | 19 | 19361735 | 19460702 | 98967 | 0.870866 | T | C | 0.172962 |
|  | rs2905422 | rs60575225 | 19 | 19361735 | 19460703 | 98968 | 0.849834 | G | A | 0.16998 |
|  | rs11085260 | rs60405730 | 19 | 19361735 | 19461492 | 99757 | 0.870866 | G | A | 0.172962 |
|  | rs2965195 | rs386577924 | 19 | 19361735 | 19463032 | 101297 | 0.870866 | G | A | 0.172962 |
|  | rs756997 | rs58784349 | 19 | 19361735 | 19467085 | 105350 | 0.870866 | C | G | 0.172962 |
|  | rs2860494 | rs58361885 | 19 | 19361735 | 19470426 | 108691 | 0.870866 | C | T | 0.172962 |
|  | rs6511029 | rs59529158 | 19 | 19361735 | 19471419 | 109684 | 0.871794 | T | G | 0.17495 |
|  | rs6511031 | rs59775331 | 19 | 19361735 | 19471506 | 109771 | 0.870866 | C | T | 0.172962 |
|  | rs57260911 | NA | 19 | 19361735 | 19477211 | 115476 | 0.833996 | T | A | 0.176938 |
|  | rs10423147 | NA | 19 | 19361735 | 19477220 | 115485 | 0.833996 | T | A | 0.176938 |
|  | rs2965201 | rs60112285 | 19 | 19361735 | 19478051 | 116316 | 0.870014 | T | C | 0.170974 |
|  | rs2965202 | rs57061055 | 19 | 19361735 | 19478454 | 116719 | 0.876263 | G | C | 0.16998 |
|  | rs2315282 | rs58898695,rs4808947 | 19 | 19361735 | 19480538 | 118803 | 0.876263 | G | A | 0.16998 |
|  | rs2905429 | rs59709127 | 19 | 19361735 | 19482558 | 120823 | 0.876263 | G | C | 0.16998 |
|  | rs2965178 | NA | 19 | 19361735 | 19482911 | 121176 | 0.876263 | G | T | 0.16998 |
|  | rs2905433 | rs11085262 | 19 | 19361735 | 19490203 | 128468 | 0.876263 | C | T | 0.16998 |
|  | rs2905434 | rs58655806 | 19 | 19361735 | 19491053 | 129318 | 0.876263 | G | T | 0.16998 |
|  | rs10406278 | NA | 19 | 19361735 | 19494725 | 132990 | 0.876263 | C | A | 0.16998 |
|  | rs2965186 | NA | 19 | 19361735 | 19497195 | 135460 | 0.876263 | G | T | 0.16998 |
|  | rs8111093 | NA | 19 | 19361735 | 19505548 | 143813 | 0.876263 | C | T | 0.16998 |
|  | rs56291054 | NA | 19 | 19361735 | 19509104 | 147369 | 0.876263 | C | G | 0.16998 |
|  | rs34487417 | NA | 19 | 19361735 | 19509701 | 147966 | 0.870014 | C | T | 0.170974 |
|  | rs2965181 | NA | 19 | 19361735 | 19512317 | 150582 | 0.876263 | C | T | 0.16998 |
|  | rs2965179 | NA | 19 | 19361735 | 19512755 | 151020 | 0.876263 | G | A | 0.16998 |
|  | rs1529745 | NA | 19 | 19361735 | 19517325 | 155590 | 0.816882 | C | G | 0.17992 |
|  | rs2060276 | NA | 19 | 19361735 | 19522491 | 160756 | 0.85525 | T | G | 0.166998 |
|  | rs2916070 | rs61009608,rs60778472,r  s17461795 | 19 | 19361735 | 19524105 | 162370 | 0.876263 | G | A | 0.16998 |
|  | rs2916072 | rs60866699 | 19 | 19361735 | 19525712 | 163977 | 0.869242 | G | A | 0.168986 |
|  | rs2916075 | rs58564638 | 19 | 19361735 | 19530590 | 168855 | 0.876263 | G | C | 0.16998 |
|  | rs11668203 | NA | 19 | 19361735 | 19531370 | 169635 | 0.876263 | C | T | 0.16998 |
|  | rs2060275 | NA | 19 | 19361735 | 19536208 | 174473 | 0.870014 | G | C | 0.170974 |
|  | rs2060274 | NA | 19 | 19361735 | 19543271 | 181536 | 0.876263 | C | T | 0.16998 |
|  | rs2965184 | rs58614130,rs17216470,r  s117763425 | 19 | 19361735 | 19545428 | 183693 | 0.870014 | A | G | 0.170974 |
|  | rs4808951 | rs17751227 | 19 | 19361735 | 19550306 | 188571 | 0.876263 | C | G | 0.16998 |
|  | rs80200208 | NA | 19 | 19361735 | 19554725 | 192990 | 0.876263 | C | T | 0.16998 |
|  | rs8105642 | NA | 19 | 19361735 | 19558468 | 196733 | 0.876263 | G | A | 0.16998 |
|  | rs8110250 | rs17684021 | 19 | 19361735 | 19559989 | 198254 | 0.876263 | G | A | 0.16998 |
|  | rs8110171 | rs17751250 | 19 | 19361735 | 19560063 | 198328 | 0.876263 | C | T | 0.16998 |
|  | rs1560687 | rs59555821,rs386537976 | 19 | 19361735 | 19562902 | 201167 | 0.876263 | G | A | 0.16998 |
|  | rs147292290 | NA | 19 | 19361735 | 19564673 | 202938 | 0.876263 | A | G | 0.16998 |
|  | rs113980591 | NA | 19 | 19361735 | 19565357 | 203622 | 0.862237 | G | A | 0.167992 |
|  | rs113841472 | NA | 19 | 19361735 | 19565482 | 203747 | 0.876263 | T | G | 0.16998 |
|  | rs75582668 | NA | 19 | 19361735 | 19569023 | 207288 | 0.876263 | C | T | 0.16998 |
|  | rs58074958 | NA | 19 | 19361735 | 19570555 | 208820 | 0.876263 | G | C | 0.16998 |
|  | rs4808958 | rs59404036 | 19 | 19361735 | 19571752 | 210017 | 0.83439 | G | A | 0.164016 |
|  | rs754256 | rs58244516,rs17751309 | 19 | 19361735 | 19578591 | 216856 | 0.876263 | G | A | 0.16998 |

|  | rs4808962 | NA | 19 | 19361735 | 19579557 | 217822 | 0.876263 | A | G | 0.16998 |
| --- | --- | --- | --- | --- | --- | --- | --- | --- | --- | --- |
|  | rs56280531 | rs57115968 | 19 | 19361735 | 19585788 | 224053 | 0.876263 | A | C | 0.16998 |
|  | rs4808204 | NA | 19 | 19361735 | 19590721 | 228986 | 0.876263 | A | G | 0.16998 |
|  | rs75667002 | NA | 19 | 19361735 | 19592232 | 230497 | 0.876263 | T | A | 0.16998 |
|  | rs11671253 | NA | 19 | 19361735 | 19604206 | 242471 | 0.876263 | C | T | 0.16998 |
|  | rs79370636 | NA | 19 | 19361735 | 19614289 | 252554 | 0.876263 | G | A | 0.16998 |
|  | rs78010159 | NA | 19 | 19361735 | 19615905 | 254170 | 0.876263 | C | T | 0.16998 |
|  | rs11670687 | NA | 19 | 19361735 | 19621752 | 260017 | 0.876263 | A | G | 0.16998 |
|  | rs11085264 | NA | 19 | 19361735 | 19621780 | 260045 | 0.876263 | A | G | 0.16998 |
|  | rs80007081 | NA | 19 | 19361735 | 19626734 | 264999 | 0.876263 | A | G | 0.16998 |
|  | rs17684164 | NA | 19 | 19361735 | 19626769 | 265034 | 0.876263 | C | T | 0.16998 |
|  | rs77254326 | NA | 19 | 19361735 | 19626781 | 265046 | 0.876263 | T | G | 0.16998 |
|  | rs16996127 | rs58339093 | 19 | 19361735 | 19627310 | 265575 | 0.876263 | T | G | 0.16998 |
|  | rs56330647 | NA | 19 | 19361735 | 19627653 | 265918 | 0.876263 | A | G | 0.16998 |
|  | rs7256028 | NA | 19 | 19361735 | 19628240 | 266505 | 0.876263 | C | T | 0.16998 |
|  | rs7245689 | NA | 19 | 19361735 | 19629530 | 267795 | 0.876263 | G | A | 0.16998 |
|  | rs78883953 | NA | 19 | 19361735 | 19630002 | 268267 | 0.870014 | G | A | 0.170974 |
|  | rs7250233 | NA | 19 | 19361735 | 19630948 | 269213 | 0.876263 | C | T | 0.16998 |
|  | rs148001625 | NA | 19 | 19361735 | 19632970 | 271235 | 0.876263 | T | C | 0.16998 |
|  | rs76095338 | NA | 19 | 19361735 | 19633908 | 272173 | 0.876263 | G | A | 0.16998 |
|  | rs78176666 | NA | 19 | 19361735 | 19636702 | 274967 | 0.876263 | T | C | 0.16998 |
|  | rs111406321 | NA | 19 | 19361735 | 19637437 | 275702 | 0.876263 | G | A | 0.16998 |
|  | rs75453386 | NA | 19 | 19361735 | 19637781 | 276046 | 0.870014 | C | T | 0.170974 |
|  | rs75525243 | NA | 19 | 19361735 | 19638218 | 276483 | 0.870014 | G | A | 0.170974 |
|  | rs45631651 | NA | 19 | 19361735 | 19638743 | 277008 | 0.870014 | A | G | 0.170974 |
|  | rs74950305 | NA | 19 | 19361735 | 19639448 | 277713 | 0.876263 | C | T | 0.16998 |
|  | rs55927782 | NA | 19 | 19361735 | 19642032 | 280297 | 0.876263 | T | C | 0.16998 |
|  | rs113527843 | NA | 19 | 19361735 | 19643343 | 281608 | 0.869242 | A | T | 0.168986 |
|  | rs8100927 | NA | 19 | 19361735 | 19643636 | 281901 | 0.869242 | C | G | 0.168986 |
|  | rs8101219 | rs12974298 | 19 | 19361735 | 19643715 | 281980 | 0.869242 | G | A | 0.168986 |
|  | rs4808968 | rs111200156 | 19 | 19361735 | 19644402 | 282667 | 0.869242 | C | T | 0.168986 |
|  | rs4808205 | NA | 19 | 19361735 | 19645493 | 283758 | 0.848279 | T | G | 0.166004 |
|  | rs4808206 | rs59829658 | 19 | 19361735 | 19645645 | 283910 | 0.85525 | C | A | 0.166998 |
|  | rs4808969 | rs6511039 | 19 | 19361735 | 19647460 | 285725 | 0.85525 | C | T | 0.166998 |
|  | rs11669730 | rs59773881 | 19 | 19361735 | 19648713 | 286978 | 0.85525 | G | A | 0.166998 |
|  | rs11670761 | NA | 19 | 19361735 | 19649327 | 287592 | 0.85525 | G | C | 0.166998 |
|  | rs11670775 | NA | 19 | 19361735 | 19649636 | 287901 | 0.855999 | A | G | 0.168986 |
|  | rs11670882 | rs57721894 | 19 | 19361735 | 19649748 | 288013 | 0.844699 | G | C | 0.172962 |
|  | rs4808970 | NA | 19 | 19361735 | 19651140 | 289405 | 0.849017 | A | G | 0.167992 |
|  | rs76790018 | NA | 19 | 19361735 | 19652157 | 290422 | 0.849017 | G | A | 0.167992 |
|  | rs8102502 | NA | 19 | 19361735 | 19652746 | 291011 | 0.827019 | T | C | 0.175944 |
|  | rs77616520 | NA | 19 | 19361735 | 19652776 | 291041 | 0.849017 | G | A | 0.167992 |
|  | rs77427798 | NA | 19 | 19361735 | 19652982 | 291247 | 0.842858 | T | C | 0.168986 |
|  | rs34067609 | NA | 19 | 19361735 | 19653524 | 291789 | 0.842858 | G | A | 0.168986 |
|  | rs7252453 | rs61625112 | 19 | 19361735 | 19654117 | 292382 | 0.831765 | G | C | 0.172962 |
|  | rs45556231 | NA | 19 | 19361735 | 19654189 | 292454 | 0.835105 | G | A | 0.166004 |
|  | rs11669558 | NA | 19 | 19361735 | 19655670 | 293935 | 0.835105 | C | T | 0.166004 |
|  | rs61744761 | NA | 19 | 19361735 | 19656615 | 294880 | 0.835105 | C | T | 0.166004 |
|  | rs1036215 | rs386512438 | 19 | 19361735 | 19657198 | 295463 | 0.821262 | C | T | 0.164016 |
| rs8056098 | rs6498143 | NA | 16 | 11138812 | 11095036 | -43776 | 0.931418 | T | G | 0.411531 |
|  | rs7184093 | NA | 16 | 11138812 | 11097849 | -40963 | 0.825188 | G | A | 0.379722 |
|  | rs7194305 | rs17804442 | 16 | 11138812 | 11099707 | -39105 | 0.935327 | A | G | 0.408549 |
|  | rs17804470 | rs61621340 | 16 | 11138812 | 11099968 | -38844 | 0.927387 | G | C | 0.406561 |
|  | rs9926615 | rs56907752 | 16 | 11138812 | 11103160 | -35652 | 0.93135 | G | A | 0.407555 |
|  | rs9935174 | rs61634253 | 16 | 11138812 | 11111347 | -27465 | 0.96339 | C | T | 0.411531 |
|  | rs3862471 | rs61473974 | 16 | 11138812 | 11113463 | -25349 | 0.975505 | G | T | 0.408549 |
|  | rs2286974 | rs17804543 | 16 | 11138812 | 11114512 | -24300 | 0.955347 | A | G | 0.411531 |
|  | rs1985372 | rs386551474 | 16 | 11138812 | 11114839 | -23973 | 0.959391 | C | T | 0.412525 |
|  | rs1985869 | NA | 16 | 11138812 | 11116271 | -22541 | 0.979579 | C | G | 0.411531 |
|  | rs1861548 | rs61423303,rs4277363 | 16 | 11138812 | 11118500 | -20312 | 0.967449 | G | C | 0.412525 |
|  | rs8049882 | NA | 16 | 11138812 | 11119486 | -19326 | 0.97554 | T | G | 0.412525 |
|  | rs8045749 | rs17804734 | 16 | 11138812 | 11119653 | -19159 | 0.979579 | A | G | 0.411531 |
|  | rs7197422 | rs60152149 | 16 | 11138812 | 11121071 | -17741 | 0.963449 | C | G | 0.413519 |
|  | rs2867168 | rs58704383,rs4427825,rs  12926317,rs10299232 | 16 | 11138812 | 11123789 | -15023 | 0.923546 | G | T | 0.40159 |
|  | rs11859698 | rs12920896 | 16 | 11138812 | 11126735 | -12077 | 0.923546 | C | T | 0.40159 |
|  | rs7197754 | rs74245516,rs57061364,r  s17230608 | 16 | 11138812 | 11130379 | -8433 | 0.884724 | G | T | 0.415507 |
|  | rs9923455 | rs60399841 | 16 | 11138812 | 11131864 | -6948 | 0.919635 | G | C | 0.400596 |

|  | rs720130 | rs74245517,rs55986336 | 16 | 11138812 | 11132633 | -6179 | 0.923546 | G | T | 0.40159 |
| --- | --- | --- | --- | --- | --- | --- | --- | --- | --- | --- |
|  | rs8056098 | rs60708787 | 16 | 11138812 | 11138812 | 0 | 1 | G | A | 0.410537 |
|  | rs3901386 | rs57952921,rs386588841,  rs17805130 | 16 | 11138812 | 11142720 | 3908 | 0.991824 | T | C | 0.412525 |
|  | rs1985872 | rs57034121 | 16 | 11138812 | 11143129 | 4317 | 0.975607 | G | C | 0.414513 |
|  | rs12924259 | rs58856490 | 16 | 11138812 | 11150098 | 11286 | 0.895011 | G | C | 0.4334 |
|  | rs2286973 | rs17805406 | 16 | 11138812 | 11154770 | 15958 | 0.979627 | G | A | 0.413519 |
|  | rs741175 | rs58962297 | 16 | 11138812 | 11159685 | 20873 | 0.892621 | T | C | 0.417495 |
|  | rs741174 | rs57059934,rs386609924 | 16 | 11138812 | 11159838 | 21026 | 0.892621 | C | T | 0.417495 |
|  | rs741173 | rs56783727 | 16 | 11138812 | 11159919 | 21107 | 0.892621 | C | A | 0.417495 |
|  | rs9888868 | NA | 16 | 11138812 | 11183563 | 44751 | 0.866308 | T | C | 0.422465 |
|  | rs11861236 | rs57500192 | 16 | 11138812 | 11183626 | 44814 | 0.866308 | T | C | 0.422465 |
|  | rs17805769 | NA | 16 | 11138812 | 11185873 | 47061 | 0.866308 | A | G | 0.422465 |
|  | rs12917656 | NA | 16 | 11138812 | 11187862 | 49050 | 0.855365 | T | C | 0.425447 |
|  | rs12917716 | NA | 16 | 11138812 | 11189148 | 50336 | 0.851434 | G | C | 0.424453 |
|  | rs12599402 | rs58745894 | 16 | 11138812 | 11189888 | 51076 | 0.851434 | T | C | 0.424453 |
|  | rs12928726 | rs17806032 | 16 | 11138812 | 11191572 | 52760 | 0.851434 | G | A | 0.424453 |
|  | rs12708717 | NA | 16 | 11138812 | 11192379 | 53567 | 0.85871 | G | C | 0.422465 |
|  | rs8061826 | NA | 16 | 11138812 | 11192787 | 53975 | 0.85871 | A | G | 0.422465 |
|  | rs12922303 | rs58954754 | 16 | 11138812 | 11193465 | 54653 | 0.85871 | A | T | 0.422465 |
|  | rs3893661 | rs60788550 | 16 | 11138812 | 11193880 | 55068 | 0.85871 | C | G | 0.422465 |
|  | rs3893660 | rs61617205,rs59772450,r  s17806098 | 16 | 11138812 | 11193930 | 55118 | 0.85871 | A | G | 0.422465 |
|  | rs3862468 | rs61606693,rs17806133 | 16 | 11138812 | 11194018 | 55206 | 0.847822 | G | C | 0.425447 |
|  | rs9941107 | rs59648104,rs58579915 | 16 | 11138812 | 11196041 | 57229 | 0.85479 | G | A | 0.421471 |
|  | rs12924667 | rs76896814 | 16 | 11138812 | 11197390 | 58578 | 0.85479 | A | T | 0.421471 |
|  | rs6498160 | rs17232123 | 16 | 11138812 | 11199447 | 60635 | 0.840004 | T | C | 0.423459 |
|  | rs998592 | rs59786189,rs17806275 | 16 | 11138812 | 11199678 | 60866 | 0.839246 | C | T | 0.417495 |
|  | rs9933507 | rs61613111 | 16 | 11138812 | 11201428 | 62616 | 0.85479 | T | C | 0.421471 |
|  | rs7193670 | NA | 16 | 11138812 | 11202705 | 63893 | 0.846991 | T | C | 0.419483 |
|  | rs9926078 | rs58851813 | 16 | 11138812 | 11203565 | 64753 | 0.850884 | G | C | 0.420477 |
|  | rs12103174 | rs57766525 | 16 | 11138812 | 11203730 | 64918 | 0.843906 | A | G | 0.424453 |
|  | rs767448 | NA | 16 | 11138812 | 11204221 | 65409 | 0.843906 | A | G | 0.424453 |
|  | rs7198004 | rs61264433,rs56875003 | 16 | 11138812 | 11207617 | 68805 | 0.850884 | A | G | 0.420477 |
|  | rs7203150 | rs60417663,rs57852080 | 16 | 11138812 | 11207722 | 68910 | 0.840004 | T | C | 0.423459 |
|  | rs12924985 | NA | 16 | 11138812 | 11208322 | 69510 | 0.843349 | G | C | 0.420477 |
|  | rs9921287 | NA | 16 | 11138812 | 11209739 | 70927 | 0.850884 | T | C | 0.420477 |
|  | rs9939397 | NA | 16 | 11138812 | 11209764 | 70952 | 0.84724 | A | G | 0.421471 |
|  | rs7186145 | rs60798354 | 16 | 11138812 | 11210878 | 72066 | 0.817665 | G | A | 0.423459 |
| rs5742909 | NO strong LD |  |  |  |  |  |  |  |  |  |
| rs11957313 | NO strong LD |  |  |  |  |  |  |  |  |  |
