# Supplementary material for: Post‐mortem multiple sclerosis lesion pathology is influenced by single nucleotide polymorphisms
Source: Brain Pathol. 2019 Jul 23;30(1):106–19. doi: 10.1111/bpa.12760 (PMC6916567; doi:10.1111/bpa.12760)
Supplement: Supplementary file 4 — Table S4. Donor characteristics: 40 MS cases that were included in qPCR analysis (PDF). [file BPA-30-106-s004.docx]

|  | number | age (years) | disease duration  (years) | post mortem pH  CSF |
| --- | --- | --- | --- | --- |
| total | 40 | 64.5 (13.15) | 30.3 (13.6) | 6.43 (0.25) |
| SP | 20 | 65.8 (14.0) | 34,5 (16,6) | 6.4 (0.21) |
| PP | 12 | 62.0 (14.45) | 26,2 (7,8) | 6.4 (0.28) |
| Relapsing | 6 | 66.3 (10.3) | 24,7 (9,1) | 6.6 (0.34) |
| male | 20 | 62.0 (12.0) | 31,3 (14,6) | 6.43 (0.27) |
| female | 20 | 67.0 (14.1) | 29,4 (12,9) | 6.43 (0.24) |

**Supplementary table 4.** Donor characteristics for 40 MS cases that were included in qPCR analysis.

Mean +/- SD is provided for age, disease duration and post-mortem pH of CSF.
