# Supplementary material for: Post‐mortem multiple sclerosis lesion pathology is influenced by single nucleotide polymorphisms
Source: Brain Pathol. 2019 Jul 23;30(1):106–19. doi: 10.1111/bpa.12760 (PMC6916567; doi:10.1111/bpa.12760)
Supplement: Supplementary file 5 — Table S5. Primer sequences and characteristics used for qPCR analysis (PDF). [file BPA-30-106-s005.docx]

| **NAME** | **SEQUENCE (5' > 3')** | **Tm ©** | **GC (%)** | **transcriptcode** | **length amplicon** | **length**  **immature amplicon** | **exon FW** | **exonRV** |
| --- | --- | --- | --- | --- | --- | --- | --- | --- |
| FAS_huFW | ATTCTGCCATAAGCCCTGTC | 62.1 | 50 | NM_000043 | 113 | 4618 | exon2 | exon3 |
| FAS_huRV | CTTTGTCTGTGTACTCCTTCCC | 62.1 | 50 | NM_000043 | 113 | 4618 | exon2 | exon3 |
| NCAN_huFW | CAACCTACCCTATGTCTGCAAG | 62.3 | 50 | NM_004386 | 146 | 3388 |  |  |
| NCAN_huRV | GCTGGGCAAATCCTTCATTG | 61.9 | 50 | NM_004386 | 146 | 3388 |  |  |
| KCNIP1_huFW | AACTCGCACTCAAGTCTTCG | 62,2 | 50 | NM_014592, NM_001034837 | 115 | 214239 | exon1 | exon2 |
| KCNIP1_huRV | GGTCATCTCCAGCTCATCTTC | 61,8 | 52,4 | NM_014592, NM_001034837 | 115 | 214239 | exon1 | exon2 |
| TM6SF2_huFW | CACGGAGTCTTCATCTGCTAC | 61.9 | 52.4 | NM_001001524 | 113 | 516 | exon4 | exon5 |
| TM6SF2_huRV | AGCCAGTAGAGTCCAAAATTCC | 62.2 | 45.5 | NM_001001524 | 113 | 516 | exon4 | exon5 |
| CILP2_huFW | CCCATGCCCAAGAAATACTCC | 62.7 | 52.4 | NM_153221 | 120 | 395 | exon6 | exon7 |
| CILP2_huRV | GTGGTAGATGCCAGCCTG | 61.8 | 61.1 | NM_153221 | 120 | 395 | exon6 | exon7 |
| HLA-A_huFW | TCCCTCACAGCTTGTAAAGTG | 62.1 | 47.6 | [NM_002116](http://www.ncbi.nlm.nih.gov/nuccore/NM_002116) | 109 | 278 | exon 7-exon 8 | exon 8 |
| HLA-A_huRV | GCAGAAACAAAGTCAGGGTTC | 61.6 | 47.6 | [NM_002116](http://www.ncbi.nlm.nih.gov/nuccore/NM_002116) | 109 | 278 | exon 7-exon8 | exon 8 |
| ACTA2_huFW | AGAGTTACGAGTTGCCTGATG | 61.8 | 47.6 | NM_001141945 | 119 | 1383 | exon 7 | exon 8 |
| ACTA2_huRV | GGTTTCATGGATGCCAGCA | 62.9 | 52.6 | NM_001141945 | 119 | 1383 | exon 7 | exon 8 |
| HAPLN4_huFW | AACTACGGGTATCGCCATAAC | 61.7 | 47.6 | NM_023002 | 114 | 428 | exon 4 | exon 5 |
| HAPLN4_huRV | TCCGGAGAAGGGTACAGG | 61.9 | 61.1 | NM_023002 | 114 | 428 | exon 4 | exon 5 |
| ZNF101_huFW | GGCTGATTTTGTCGTGTGG | 61.3 | 52.6 | [NM_033204](http://www.ncbi.nlm.nih.gov/nuccore/NM_033204) | 109 | 9006 | exon 1 | exon 2 |
| ZNF101_huRV | TCACAGCCACATCCTCAAAG | 62.1 | 50.0 | [NM_033204](http://www.ncbi.nlm.nih.gov/nuccore/NM_033204) | 109 | 9006 | exon 1 | exon 2 |
| LPAR2_huFW | AGCCTGGTCAAGACTGTTG | 61.8 | 52.6 | NM_004720 | 116 | 2080 | exon2 | exon 3 |
| LPAR2_huRV | ACATTGCAGGACTCACAGC | 62.4 | 52.6 | NM_004720 | 116 | 2080 | exon2 | exon3 |
| TSSK6_huFW | CTTAGACCCGAGATTGGAGTC | 61.2 | 52.4 | NM_032037 | 120 | 120 | exon 1 | exon 1 |
| TSSK6_huRV | CCCAGCTTATAACCGAGTTCG | 62.2 | 52.4 | NM_032037 | 120 | 120 | exon 1 | exon 1 |
| YJEFN3_huFW | CATCTTCTACCCCACACGC | 62.0 | 57.9 | NM_198537 | 119 | 197 | exon5 | exon5-exon6 |
| YJEFN3_huRV | CTTCGTTAATGAGCTGCACC | 61.1 | 50.0 | NM_198537 | 119 | 197 | exon5 | exon5-exon6 |
| C5orf58_huFW | CCAGTTTTTTTACAGTGGCTCTG | 62.2 | 43.5 | NM_001102609 | 119 | 2004 | exon1-exon2 | exon3 |
| C5orf58_huRV | GCTTATGATCAGTAACACGCTTC | 61.5 | 43.5 | NM_001102609 | 119 | 2004 | exon1-exon2 | exon3 |
| CLEC16A_huFW | GAAGGCTAAAGGTACAGAGGG | 61.5 | 52.4 | NM_015226 | 90 | 16977 | exon11 | exon12 |
| CLEC16A_huRV | TTGCTACGCTCCATGATCAC | 62.2 | 50.0 | NM_015226 | 90 | 16977 | exon11 | exon12 |
| CTLA4_huFW | GGACTCTACATCTGCAAGGTG | 62.0 | 52.4 | NM_005214 | 106 | 550 | exon2 | exon3 |
| CTLA4_huRV | AATCTGGGCACGGTTCTG | 61.9 | 55.6 | NM_005214 | 106 | 550 | exon2 | exon3 |
| HLA-A_huFW | TCCCTCACAGCTTGTAAAGTG | 62.1 | 47.6 | [NM_002116](http://www.ncbi.nlm.nih.gov/nuccore/NM_002116) | 109 | 278 | exon 7-exon 8 | exon 8 |
| HLA-A_huRV | GCAGAAACAAAGTCAGGGTTC | 61.6 | 47.6 | [NM_002116](http://www.ncbi.nlm.nih.gov/nuccore/NM_002116) | 109 | 278 | exon 7-exon8 | exon 8 |

**Supplementary table 5.** Primer sequences and characteristics used for qPCR analysis.
