# Supplementary material for: Post‐mortem multiple sclerosis lesion pathology is influenced by single nucleotide polymorphisms
Source: Brain Pathol. 2019 Jul 23;30(1):106–19. doi: 10.1111/bpa.12760 (PMC6916567; doi:10.1111/bpa.12760)
Supplement: Supplementary file 6 — Table S6. FAS in peripheral lymphocyte populations (PDF). [file BPA-30-106-s006.docx]

|  | Fas (protein copy numbers) |
| --- | --- |
| Intensity_B.naive_steady-state | 0 |
| Intensity_B.memory_steady-state | 0,5 |
| Intensity_B.plasma_steady-state | 0,2 |
| Intensity_T4.naive_steady-state | 0,2 |
| Intensity_T4.CM_steady-state | 4,2 |
| Intensity_T4.EM_steady-state | 6,6 |
| Intensity_T4.EMRA_steady-state | 0,8 |
| Intensity_nTregs_steady-state (CD4+, CD25 low, CD45 RA low, IL7R low) | 0,7 |
| Intensity_mTregs_steady-state (CD4+, CD25 high, CD45RA low, IL7R low) | 14,9 |
| Intensity_Th1_steady-state | 2,1 |
| Intensity_Th2_steady-state | 3,9 |
| Intensity_Th17_steady-state | 7 |
| Intensity_T8.naive_steady-state | 0,1 |
| Intensity_T8.CM_steady-state | 4,3 |
| Intensity_T8.EM_steady-state | 4 |
| Intensity_T8.EMRA_steady-state | 1,7 |
| Intensity_mDC_steady-state | 0,6 |
| Intensity_pDC_steady-state | 0,3 |
| Intensity_Eosinophil_steady-state | 0,2 |
| Intensity_Neutrophil_steady-state | 1 |
| Intensity_Basophil_steady-state | 0,5 |
| Intensity_MO.classical_steady-state | 0,4 |
| Intensity_MO.intermediate_steady-state | 1 |
| Intensity_MO.nonclassical_steady-state | 0,8 |
| Intensity_NK.bright_steady-state | 0,1 |
| Intensity_NK.dim_steady-state | 0,6 |
| Intensity_Thrombocyte_steady-state | 0 |
| Intensity_Erythrocyte_steady-state | 0 |

**Supplementary table 6.** FAS in peripheral lymphocyte populations Mass spectrometry data extracted from www.immprot.org
