# Supplementary material for: Post‐mortem multiple sclerosis lesion pathology is influenced by single nucleotide polymorphisms
Source: Brain Pathol. 2019 Jul 23;30(1):106–19. doi: 10.1111/bpa.12760 (PMC6916567; doi:10.1111/bpa.12760)
Supplement: Supplementary file 7 — Table S7. Donor and tissue characteristics: cases included in FACS analysis (PDF). [file BPA-30-106-s007.docx]

| nhb | tissue type | PA diagnosis | Cause of death | age (years) | gender |
| --- | --- | --- | --- | --- | --- |
| 16-034 | brain WM | MSA | Euthanasia | 69 | F |
| 15-050 | brain WM | Parkinson and Alzheimer | Cachexia and delirium | 84 | F |
| 17-113 | brain WM | Alzheimer | Euthanasia | 63 | M |
| 14-034 | pbmc | Lewybody dementia | Atrial fibrillation, Asystole | 72 | M |
| 14-048 | pbmc | Alzheimer | Old age | 111 | F |
| 14-043 | pbmc | Breast cancer | Metastasized mammacarcinoma | 60 | F |

**Supplementary table 7.** Donor and tissue characteristics included in the FACS analysis
