# Supplementary material for: Post‐mortem multiple sclerosis lesion pathology is influenced by single nucleotide polymorphisms
Source: Brain Pathol. 2019 Jul 23;30(1):106–19. doi: 10.1111/bpa.12760 (PMC6916567; doi:10.1111/bpa.12760)
Supplement: Supplementary file 8 — Table S8. Results for all statistical tests performed (PDF). [file BPA-30-106-s008.pdf]

Supplementary table 8. Results for all statistical tests performed.
p value is showing unadjusted p value of binomial (Grey_matter_lesions) or quasibinomial (active, remyel, mAIL) generalized linear models and linear models (Log_Lesion_load_BRS). Adjusted p value shows p value after multiple testing correction using Benjamini-Hochberg method.
active = proportion of active lesions per case . mAIL = proportion of mixed active/inactive lesions per case, remyel = proportion of remyelinated lesions per case, Log_Lesion_load_BRS= log transformed number of lesions in standardly dissected brain stem tissue blocks, Grey_matter_lesions = yes/no presence of cortical grey matter lesions, MMAS= microglia/macrophage activity score.

					POST HOC TESTS					n				
locus	gene	outcome measure	p value	adjusted p-value	Heteroz vs Homoz. major	Homoz. minor vs Homoz.major	Homoz. minor vs Heteroz.	test statistic	degrees of freedom	Homoz. major	Heteroz.	Homoz. minor	Major_allele	Minor_allele
rs1064395	NCAN	Grey_matter_lesions	2,E-05	0,010	2,E-05	0,126	1,000	χ2 = 22.1	2	132	47	5	G	A
rs2234978	FAS	active	9,E-05	0,018	3,E-04	0,004	0,711	χ2 = 18.7	2	81	83	22	C	T
rs3130253	MOG	active	9,E-05	0,018	1,E-04	0,450	0,120	χ2 = 18.7	2	134	45	4	G	A
rs11957313	KCNIP1	active	3,E-04	0,047	0,015	0,026	0,228	χ2 = 16.0	2	132	49	6	G	A
rs8056098	CLEC16A	mAIL	4,E-04	0,047	0,965	0,002	9,E-04	χ2 = 15.4	2	75	89	20	G	A
rs5742909	CTLA4	remyel	5,E-04	0,047	2,E-04	0,941	0,384	χ2 = 15.4	2	154	27	4	C	T
rs2399849	CAMK1D	remyel	6,E-04	0,054				χ2 = 14.8	2	134	46	2	G	A
rs2116078	KCNB2	active	7,E-04	0,054				χ2 = 14.5	2	49	93	38	G	T
rs6994992	NRG1	active	9,E-04	0,061				χ2 = 14.0	2	70	90	28	C	T
rs2074897	NDUFS7	active	0,002	0,099				χ2 = 12.8	2	54	94	33	G	A
rs9892479	ASIC2	remyel	0,002	0,099				χ2 = 12.5	2	165	22	1	G	T
rs2766051	DOCK1	mAIL	0,002	0,099				χ2 = 12.5	2	145	34	4	G	A
rs72928038	BACH2	remyel	0,002	0,099				χ2 = 12.3	2	119	67	2	G	A
rs6994992	NRG1	mAIL	0,003	0,120				χ2 = 11.7	2	70	90	28	C	T
rs17398267	LHCGR	active	0,003	0,120				χ2 = 11.7	2	112	58	14	T	G
rs10052957	NR3C1	mAIL	0,004	0,143				χ2 = 11.2	2	79	86	22	G	A
rs2039485	NUBPL	active	0,004	0,153				χ2 = 10.9	2	104	74	10	T	C
rs3116496	CD28	remyel	0,004	0,153				χ2 = 10.8	2	134	45	4	T	C
rs868824	IMMP2L	active	0,005	0,153				χ2 = 10.7	2	62	81	40	T	C
rs2796267	CD46	mAIL	0,005	0,154				χ2 = 10.5	2	54	86	36	A	G
rs1133763	CCL8	Grey_matter_lesions	0,005	0,154				χ2 = 10.5	2	124	58	3	A	C
rs755622	MIF-AS1	active	0,006	0,164				χ2 = 10.3	2	119	63	3	C	G
rs2569190	CD14	remyel	0,006	0,164				χ2 = 10.2	2	43	96	39	G	A
rs263153	LOC153910	remyel	0,007	0,165				χ2 = 7.3	1	148	37	0	G	T
rs4819554	IL17RA	active	0,007	0,165				χ2 = 9.9	2	145	38	4	A	G
rs10078091	CDH10	mAIL	0,007	0,165				χ2 = 9.9	2	95	76	14	G	A
rs17505688	NTNG1	remyel	0,007	0,170				χ2 = 9.8	2	154	26	2	T	C
rs423904	IL1RN	active	0,008	0,172				χ2 = 9.7	2	91	80	8	C	T
rs11666377	CPAMD8	mAIL	0,009	0,174				χ2 = 9.5	2	134	52	3	C	T
rs1883832	CD40	active	0,009	0,174				χ2 = 9.4	2	109	68	11	C	T
rs2069727	IFNG	Log_Lesion_load_BRS	0,009	0,174				F = 4.8	2/148	45	92	43	A	G
rs1597944	CHRND	remyel	0,010	0,174				χ2 = 9.3	2	56	87	43	T	C
rs11652878	ITGAE	mAIL	0,010	0,174				χ2 = 9.3	2	160	25	1	A	G
rs10078091	CDH10	active	0,010	0,174				χ2 = 9.3	2	95	76	14	G	A
rs7211577	COX10	mAIL	0,011	0,190				χ2 = 9.0	2	57	96	34	G	A
rs7914524	AK124226	MMAS	0,013	0,217				χ2 = 8.7	2	110	62	10	C	T
rs5742909	CTLA4	Grey_matter_lesions	0,014	0,219				χ2 = 8.6	2	154	27	4	C	T
rs13067869	NLGN1	active	0,014	0,219				χ2 = 8.5	2	147	30	2	T	G
rs305124	UBE2K	active	0,014	0,219				χ2 = 8.5	2	151	33	1	A	G
rs13387792	MYT1L	Log_Lesion_load_BRS	0,014	0,219				F = 6.1	1/153	164	23	0	G	A
rs10243024	MET	active	0,015	0,219				χ2 = 8.4	2	119	54	9	G	A
rs3116496	CD28	Log_Lesion_load_BRS	0,015	0,219				F = 4.3	2/150	134	45	4	T	C
rs2796267	CD46	active	0,016	0,233				χ2 = 8.2	2	54	86	36	A	G
rs2399849	CAMK1D	MMAS	0,017	0,233				χ2 = 8.2	2	134	46	2	G	A
rs1761667	CD36	active	0,018	0,240				χ2 = 8.1	2	58	85	39	A	G
rs2236851	RUNX3	active	0,018	0,241				χ2 = 8.0	2	126	53	7	C	T
rs5742909	CTLA4	mAIL	0,019	0,241				χ2 = 8.0	2	154	27	4	C	T
rs12644284	TRIM2	active	0,019	0,241				χ2 = 7.9	2	103	69	13	A	G
rs8056098	CLEC16A	remyel	0,019	0,241				χ2 = 7.9	2	75	89	20	G	A
rs2234978	FAS	MMAS	0,020	0,241				χ2 = 7.9	2	81	83	22	C	T
rs2399849	CAMK1D	mAIL	0,021	0,250				χ2 = 7.7	2	134	46	2	G	A
rs17157903	RELN	remyel	0,022	0,250				χ2 = 7.7	2	150	34	3	C	T
rs4880213	GRIN1	remyel	0,022	0,250				χ2 = 7.7	2	63	99	24	C	T
rs231775	CTLA4	remyel	0,022	0,252				χ2 = 7.6	2	73	95	21	A	G
rs1386330	RAB38	active	0,024	0,260				χ2 = 7.5	2	142	39	1	T	C
rs716595	MXI1	remyel	0,024	0,260				χ2 = 5.1	1	144	41	1	G	A
rs3130253	MOG	remyel	0,026	0,276				χ2 = 7.3	2	134	45	4	G	A
rs11719646	ERC2	mAIL	0,026	0,276				χ2 = 7.3	2	61	99	23	A	G
rs2853744	SPP1	active	0,027	0,284				χ2 = 7.2	2	171	15	2	G	T
rs11765693	YWHAG	mAIL	0,030	0,306				χ2 = 7.0	2	81	87	13	A	G
rs9480865	FOXO3	Grey_matter_lesions	0,030	0,306				χ2 = 7.0	2	142	39	5	T	C
rs2803418	PCSK5	active	0,032	0,319				χ2 = 6.9	2	91	80	10	G	T
rs1448239	GRIN2A	remyel	0,035	0,333				χ2 = 6.7	2	130	53	1	G	C
rs3116496	CD28	Grey_matter_lesions	0,035	0,333				χ2 = 6.7	2	134	45	4	T	C
rs6917747	IGF2R	active	0,035	0,333				χ2 = 6.7	2	137	44	6	G	A
rs7211577	COX10	Grey_matter_lesions	0,037	0,335				χ2 = 6.6	2	57	96	34	G	A
rs1386330	RAB38	Log_Lesion_load_BRS	0,037	0,335				F = 3.4	2/150	142	39	1	T	C
rs876493	PNMT	mAIL	0,037	0,335				χ2 = 6.6	2	71	81	34	A	G
rs12644284	TRIM2	remyel	0,040	0,351				χ2 = 6.5	2	103	69	13	A	G
rs261902	BICD1	Grey_matter_lesions	0,043	0,372				χ2 = 6.3	2	114	57	8	C	T
rs8192678	PPARGC1A	mAIL	0,044	0,376				χ2 = 6.2	2	90	77	17	G	A
rs6994992	NRG1	MMAS	0,045	0,376				χ2 = 6.2	2	70	90	28	C	T
rs1133763	CCL8	remyel	0,045	0,376				χ2 = 6.2	2	124	58	3	A	C
rs13019537	LHCGR	active	0,047	0,389				χ2 = 6.1	2	126	57	4	C	G
rs1869410	SOX11	active	0,050	0,408				χ2 = 6.0	2	88	71	19	T	C
rs10977017	PTPRD	MMAS	0,051	0,408				χ2 = 5.9	2	124	52	5	G	A
rs79877597	IL17A	MMAS	0,051	0,408				χ2 = 5.9	2	127	53	6	C	A
rs6917747	IGF2R	Log_Lesion_load_BRS	0,053	0,411				F = 3.0	2/152	137	44	6	G	A
rs2853744	SPP1	remyel	0,053	0,411				χ2 = 5.9	2	171	15	2	G	T
rs299175	NLRP11	active	0,055	0,415				χ2 = 5.8	2	53	87	39	C	T
rs4819554	IL17RA	Grey_matter_lesions	0,055	0,415				χ2 = 5.8	2	145	38	4	A	G
rs2116078	KCNB2	remyel	0,056	0,415				χ2 = 5.8	2	49	93	38	G	T
rs12111597	NPSR1,NPSR1-AS1	mAIL	0,057	0,415				χ2 = 5.7	2	99	73	10	G	A
rs9892479	ASIC2	mAIL	0,057	0,415				χ2 = 5.7	2	165	22	1	G	T
rs1064395	NCAN	remyel	0,059	0,415				χ2 = 5.7	2	132	47	5	G	A
rs2107538	CCL5	remyel	0,059	0,415				χ2 = 5.7	2	119	59	5	C	T
rs11719646	ERC2	remyel	0,059	0,415				χ2 = 5.7	2	61	99	23	A	G
rs7914524	AK124226	active	0,061	0,422				χ2 = 5.6	2	110	62	10	C	T
rs7211577	COX10	MMAS	0,061	0,422				χ2 = 5.6	2	57	96	34	G	A

rs4880213	GRIN1	MMAS	0,062	0,422				χ2 = 5.6	2	63	99	24	C	T
rs10505082	ZFPM2	mAIL	0,063	0,422				χ2 = 5.5	2	123	62	1	G	A
rs7744583	ARID1B	remyel	0,064	0,422				χ2 = 5.5	2	78	85	20	G	A
rs2399849	CAMK1D	active	0,065	0,422				χ2 = 5.5	2	134	46	2	G	A
rs1386330	RAB38	MMAS	0,066	0,422				χ2 = 5.4	2	142	39	1	T	C
rs6917747	IGF2R	MMAS	0,066	0,422				χ2 = 5.4	2	137	44	6	G	A
rs10243024	MET	Grey_matter_lesions	0,066	0,422				χ2 = 5.4	2	119	54	9	G	A
rs744166	STAT3	active	0,067	0,423				χ2 = 5.4	2	63	90	30	T	C
rs404694	MAF	active	0,068	0,423				χ2 = 5.4	2	92	79	14	A	C
rs1133763	CCL8	MMAS	0,070	0,424				χ2 = 5.3	2	124	58	3	A	C
rs10977017	PTPRD	remyel	0,070	0,424				χ2 = 5.3	2	124	52	5	G	A
rs4880213	GRIN1	Grey_matter_lesions	0,070	0,424				χ2 = 5.3	2	63	99	24	C	T
rs16925027	KDM4C	remyel	0,071	0,425				χ2 = 5.3	2	111	62	8	A	G
rs1869410	SOX11	MMAS	0,071	0,425				χ2 = 5.3	2	88	71	19	T	C
rs4953911	MGAT5	Log_Lesion_load_BRS	0,074	0,433				F = 2.6	2/151	103	74	8	A	T
rs868824	IMMP2L	Log_Lesion_load_BRS	0,075	0,433				F = 2.6	2/150	62	81	40	T	C
rs11765693	YWHAG	Log_Lesion_load_BRS	0,075	0,433				F = 2.6	2/149	81	87	13	A	G
rs11719646	ERC2	active	0,078	0,443				χ2 = 5.1	2	61	99	23	A	G
rs10243024	MET	remyel	0,080	0,454				χ2 = 5.0	2	119	54	9	G	A
rs2074897	NDUFS7	Grey_matter_lesions	0,082	0,461				χ2 = 5.0	2	54	94	33	G	A
rs7253363	BC039523	Grey_matter_lesions	0,083	0,461				χ2 = 3.0	1	180	7	0	G	T
rs3865444	CD33	Log_Lesion_load_BRS	0,084	0,461				F = 2.5	2/154	86	75	28	G	T
rs1927457	SVIL	MMAS	0,087	0,472				χ2 = 4.9	2	79	84	14	T	C
rs2116078	KCNB2	MMAS	0,088	0,472				χ2 = 4.9	2	49	93	38	G	T
rs2803418	PCSK5	MMAS	0,088	0,472				χ2 = 4.9	2	91	80	10	G	T
rs72928038	BACH2	active	0,089	0,472				χ2 = 4.8	2	119	67	2	G	A
rs1597944	CHRND	mAIL	0,091	0,478				χ2 = 4.8	2	56	87	43	T	C
rs79877597	IL17A	Log_Lesion_load_BRS	0,094	0,478				F = 2.4	2/152	127	53	6	C	A
rs305124	UBE2K	mAIL	0,094	0,478				χ2 = 4.7	2	151	33	1	A	G
rs752092	CHSY1	Grey_matter_lesions	0,095	0,478				χ2 = 4.7	2	85	81	24	T	C
rs1318	PITPNC1	MMAS	0,095	0,478				χ2 = 4.7	2	117	60	5	A	G
rs10243024	MET	MMAS	0,095	0,478				χ2 = 4.7	2	119	54	9	G	A
rs4819554	IL17RA	remyel	0,096	0,478				χ2 = 4.7	2	145	38	4	A	G
rs5742909	CTLA4	Log_Lesion_load_BRS	0,096	0,478				F = 2.4	2/151	154	27	4	C	T
rs8192678	PPARGC1A	active	0,097	0,478				χ2 = 4.7	2	90	77	17	G	A
rs263153	LOC153910	Grey_matter_lesions	0,098	0,478				χ2 = 2.7	1	148	37	0	G	T
rs5742909	CTLA4	active	0,101	0,493				χ2 = 4.6	2	154	27	4	C	T
rs10243024	MET	mAIL	0,105	0,493				χ2 = 4.5	2	119	54	9	G	A
rs6994992	NRG1	Grey_matter_lesions	0,105	0,493				χ2 = 4.5	2	70	90	28	C	T
rs13067869	NLGN1	MMAS	0,107	0,493				χ2 = 4.5	2	147	30	2	T	G
rs6917747	IGF2R	Grey_matter_lesions	0,108	0,493				χ2 = 4.5	2	137	44	6	G	A
rs1761667	CD36	remyel	0,108	0,493				χ2 = 4.4	2	58	85	39	A	G
rs4747075	ADAMTS14	MMAS	0,110	0,493				χ2 = 4.4	2	86	58	30	G	A
rs1133763	CCL8	mAIL	0,111	0,493				χ2 = 4.4	2	124	58	3	A	C
rs6994992	NRG1	Log_Lesion_load_BRS	0,112	0,493				F = 2.2	2/153	70	90	28	C	T
rs11719646	ERC2	Log_Lesion_load_BRS	0,114	0,493				F = 2.2	2/149	61	99	23	A	G
rs3212227	IL12B	mAIL	0,115	0,493				χ2 = 4.3	2	125	44	7	A	C
rs2766051	DOCK1	remyel	0,115	0,493				χ2 = 4.3	2	145	34	4	G	A
rs1061170	CFH	mAIL	0,116	0,493				χ2 = 4.3	2	70	84	24	T	C
rs1597944	CHRND	active	0,116	0,493				χ2 = 4.3	2	56	87	43	T	C
rs9319189	SLITRK6	mAIL	0,117	0,493				χ2 = 4.3	2	92	76	13	G	A
rs1318	PITPNC1	Log_Lesion_load_BRS	0,118	0,493				F = 2.2	2/148	117	60	5	A	G
rs17157903	RELN	active	0,118	0,493				χ2 = 4.3	2	150	34	3	C	T
rs2074897	NDUFS7	remyel	0,118	0,493				χ2 = 4.3	2	54	94	33	G	A
rs2234978	FAS	Log_Lesion_load_BRS	0,119	0,493				F = 2.2	2/152	81	83	22	C	T
rs337718	CBLN2	mAIL	0,120	0,493				χ2 = 4.2	2	91	78	15	C	T
rs1927457	SVIL	mAIL	0,120	0,493				χ2 = 4.2	2	79	84	14	T	C
rs3865444	CD33	Grey_matter_lesions	0,121	0,493				χ2 = 4.2	2	86	75	28	G	T
rs6198	NR3C1	mAIL	0,121	0,493				χ2 = 4.2	2	118	54	9	T	C
rs8192678	PPARGC1A	remyel	0,121	0,493				χ2 = 4.2	2	90	77	17	G	A
rs41423247	NR3C1	remyel	0,122	0,493				χ2 = 4.2	2	88	65	31	G	C
rs2803418	PCSK5	mAIL	0,122	0,493				χ2 = 4.2	2	91	80	10	G	T
rs1883832	CD40	remyel	0,123	0,494				χ2 = 4.2	2	109	68	11	C	T
rs2234978	FAS	remyel	0,125	0,499				χ2 = 4.2	2	81	83	22	C	T
rs6941421	JARID2	mAIL	0,126	0,500				χ2 = 4.1	2	62	95	28	T	C
rs4819554	IL17RA	Log_Lesion_load_BRS	0,129	0,509				F = 2.1	2/152	145	38	4	A	G
rs10516537	DKK2	MMAS	0,133	0,521				χ2 = 4.0	2	132	45	7	C	A
rs8056098	CLEC16A	Log_Lesion_load_BRS	0,135	0,525				F = 2.0	2/150	75	89	20	G	A
rs752092	CHSY1	mAIL	0,136	0,528				χ2 = 4.0	2	85	81	24	T	C
rs11750073	CTNND2	Grey_matter_lesions	0,138	0,529				χ2 = 4.0	2	122	55	7	C	T
rs876493	PNMT	active	0,140	0,530				χ2 = 3.9	2	71	81	34	A	G
rs3212227	IL12B	Grey_matter_lesions	0,140	0,530				χ2 = 3.9	2	125	44	7	A	C
rs41423247	NR3C1	mAIL	0,140	0,530				χ2 = 3.9	2	88	65	31	G	C
rs4747075	ADAMTS14	remyel	0,141	0,531				χ2 = 3.9	2	86	58	30	G	A
rs876493	PNMT	Log_Lesion_load_BRS	0,143	0,535				F = 2.0	2/151	71	81	34	A	G
rs2037815	CASP8	Grey_matter_lesions	0,146	0,538				χ2 = 3.9	2	46	99	42	A	G
rs2069727	IFNG	active	0,146	0,538				χ2 = 3.8	2	45	92	43	A	G
rs5673	PTGER3	remyel	0,147	0,538				χ2 = 2.1	1	169	17	0	A	T
rs2766051	DOCK1	MMAS	0,148	0,538				χ2 = 3.8	2	145	34	4	G	A
rs4747075	ADAMTS14	mAIL	0,150	0,541				χ2 = 3.8	2	86	58	30	G	A
rs263153	LOC153910	MMAS	0,153	0,541				χ2 = 2.0	1	148	37	0	G	T
rs1137933	NOS2	Grey_matter_lesions	0,153	0,541				χ2 = 3.8	2	115	65	8	G	A
rs11765693	YWHAG	remyel	0,153	0,541				χ2 = 3.7	2	81	87	13	A	G
rs2107538	CCL5	active	0,154	0,541				χ2 = 3.7	2	119	59	5	C	T
rs2107538	CCL5	Grey_matter_lesions	0,154	0,541				χ2 = 3.7	2	119	59	5	C	T
rs2069727	IFNG	remyel	0,158	0,551				χ2 = 3.7	2	45	92	43	A	G
rs333	CCR5	MMAS	0,159	0,553				χ2 = 3.7	2	154	26	5	AGAATTTCCAGACA	-
rs79877597	IL17A	Grey_matter_lesions	0,160	0,553				χ2 = 3.7	2	127	53	6	C	A
rs3130253	MOG	MMAS	0,161	0,554				χ2 = 3.7	2	134	45	4	G	A
rs11666377	CPAMD8	MMAS	0,163	0,554				χ2 = 3.6	2	134	52	3	C	T
rs1064395	NCAN	mAIL	0,163	0,554				χ2 = 3.6	2	132	47	5	G	A
rs876493	PNMT	Grey_matter_lesions	0,164	0,555				χ2 = 3.6	2	71	81	34	A	G
rs4953911	MGAT5	remyel	0,167	0,559				χ2 = 3.6	2	103	74	8	A	T
rs11666377	CPAMD8	active	0,168	0,559				χ2 = 3.6	2	134	52	3	C	T
rs2803418	PCSK5	Grey_matter_lesions	0,169	0,559				χ2 = 3.6	2	91	80	10	G	T

rs17398267	LHCGR	Log_Lesion_load_BRS	0,169	0,559				F = 1.8	2/150	112	58	14	T	G
rs10505082	ZFPM2	active	0,170	0,559				χ2 = 3.5	2	123	62	1	G	A
rs2107538	CCL5	Log_Lesion_load_BRS	0,172	0,559				F = 1.8	2/150	119	59	5	C	T
rs2803418	PCSK5	remyel	0,172	0,559				χ2 = 3.5	2	91	80	10	G	T
rs10052957	NR3C1	Log_Lesion_load_BRS	0,175	0,566				F = 1.8	2/152	79	86	22	G	A
rs17398267	LHCGR	MMAS	0,179	0,577				χ2 = 3.4	2	112	58	14	T	G
rs6917747	IGF2R	mAIL	0,184	0,584				χ2 = 3.4	2	137	44	6	G	A
rs6190	NR3C1	Grey_matter_lesions	0,184	0,584				χ2 = 3.4	2	166	20	1	G	A
rs3130253	MOG	Log_Lesion_load_BRS	0,184	0,584				F = 1.7	2/150	134	45	4	G	A
rs1927457	SVIL	Log_Lesion_load_BRS	0,185	0,584				F = 1.7	2/145	79	84	14	T	C
rs6917747	IGF2R	remyel	0,186	0,584				χ2 = 3.4	2	137	44	6	G	A
rs156429	GPNMB	Grey_matter_lesions	0,190	0,595				χ2 = 3.3	2	62	91	32	T	C
rs2569190	CD14	Grey_matter_lesions	0,192	0,597				χ2 = 3.3	2	43	96	39	G	A
rs10178552	ASXL2	active	0,195	0,600				χ2 = 3.3	2	73	87	25	C	T
rs72928038	BACH2	Log_Lesion_load_BRS	0,195	0,600				F = 1.7	2/153	119	67	2	G	A
rs1318	PITPNC1	active	0,197	0,602				χ2 = 3.3	2	117	60	5	A	G
rs305124	UBE2K	remyel	0,200	0,608				χ2 = 3.2	2	151	33	1	A	G
rs4880213	GRIN1	active	0,202	0,613				χ2 = 3.2	2	63	99	24	C	T
rs10977017	PTPRD	Grey_matter_lesions	0,211	0,635				χ2 = 3.1	2	124	52	5	G	A
rs10052957	NR3C1	remyel	0,212	0,635				χ2 = 3.1	2	79	86	22	G	A
rs876493	PNMT	MMAS	0,213	0,635				χ2 = 3.1	2	71	81	34	A	G
rs11765693	YWHAG	Grey_matter_lesions	0,214	0,636				χ2 = 3.1	2	81	87	13	A	G
rs13067869	NLGN1	mAIL	0,220	0,647				χ2 = 3.0	2	147	30	2	T	G
rs423904	IL1RN	Grey_matter_lesions	0,221	0,647				χ2 = 3.0	2	91	80	8	C	T
rs17398267	LHCGR	mAIL	0,222	0,647				χ2 = 3.0	2	112	58	14	T	G
rs1318	PITPNC1	mAIL	0,222	0,647				χ2 = 3.0	2	117	60	5	A	G
rs337718	CBLN2	active	0,223	0,647				χ2 = 3.0	2	91	78	15	C	T
rs2803418	PCSK5	Log_Lesion_load_BRS	0,225	0,650				F = 1.5	2/149	91	80	10	G	T
rs1799987	CCR5	active	0,228	0,656				χ2 = 3.0	2	55	88	43	A	G
rs6190	NR3C1	remyel	0,233	0,664				χ2 = 2.9	2	166	20	1	G	A
rs12127450	AGBL4	remyel	0,233	0,664				χ2 = 2.9	2	133	47	3	T	C
rs7253363	BC039523	mAIL	0,238	0,673				χ2 = 1.4	1	180	7	0	G	T
rs17505688	NTNG1	mAIL	0,239	0,673				χ2 = 2.9	2	154	26	2	T	C
rs2234978	FAS	mAIL	0,240	0,674				χ2 = 2.9	2	81	83	22	C	T
rs2116078	KCNB2	Log_Lesion_load_BRS	0,244	0,678				F = 1.4	2/146	49	93	38	G	T
rs231775	CTLA4	active	0,244	0,678				χ2 = 2.8	2	73	95	21	A	G
rs41423247	NR3C1	Log_Lesion_load_BRS	0,246	0,678				F = 1.4	2/150	88	65	31	G	C
rs2116078	KCNB2	mAIL	0,246	0,678				χ2 = 2.8	2	49	93	38	G	T
rs1448239	GRIN2A	active	0,250	0,680				χ2 = 2.8	2	130	53	1	G	C
rs263153	LOC153910	Log_Lesion_load_BRS	0,250	0,680				F = 1.3	1/153	148	37	0	G	T
rs9319189	SLITRK6	active	0,251	0,680				χ2 = 2.8	2	92	76	13	G	A
rs1061170	CFH	Log_Lesion_load_BRS	0,252	0,680				F = 1.4	2/147	70	84	24	T	C
rs3865444	CD33	remyel	0,256	0,680				χ2 = 2.7	2	86	75	28	G	T
rs2796267	CD46	remyel	0,256	0,680				χ2 = 2.7	2	54	86	36	A	G
rs11957313	KCNIP1	remyel	0,257	0,680				χ2 = 2.7	2	132	49	6	G	A
rs7744583	ARID1B	mAIL	0,258	0,680				χ2 = 2.7	2	78	85	20	G	A
rs1061170	CFH	MMAS	0,259	0,680				χ2 = 2.7	2	70	84	24	T	C
rs337718	CBLN2	remyel	0,259	0,680				χ2 = 2.7	2	91	78	15	C	T
rs299175	NLRP11	mAIL	0,259	0,680				χ2 = 2.7	2	53	87	39	C	T
rs3212227	IL12B	active	0,260	0,680				χ2 = 2.7	2	125	44	7	A	C
rs1800682	FAS,ACTA2	MMAS	0,266	0,693				χ2 = 2.6	2	43	100	41	T	C
rs261902	BICD1	mAIL	0,268	0,693				χ2 = 2.6	2	114	57	8	C	T
rs6190	NR3C1	MMAS	0,269	0,693				χ2 = 2.6	2	166	20	1	G	A
rs1927457	SVIL	remyel	0,270	0,693				χ2 = 2.6	2	79	84	14	T	C
rs6941421	JARID2	MMAS	0,281	0,718				χ2 = 2.5	2	62	95	28	T	C
rs72928038	BACH2	mAIL	0,282	0,718				χ2 = 2.5	2	119	67	2	G	A
rs1799987	CCR5	mAIL	0,287	0,723				χ2 = 2.5	2	55	88	43	A	G
rs12127450	AGBL4	mAIL	0,288	0,723				χ2 = 2.5	2	133	47	3	T	C
rs1137933	NOS2	mAIL	0,289	0,723				χ2 = 2.5	2	115	65	8	G	A
rs2037815	CASP8	active	0,290	0,723				χ2 = 2.5	2	46	99	42	A	G
rs2074897	NDUFS7	mAIL	0,290	0,723				χ2 = 2.5	2	54	94	33	G	A
rs10977017	PTPRD	active	0,291	0,723				χ2 = 2.5	2	124	52	5	G	A
rs10505082	ZFPM2	remyel	0,294	0,729				χ2 = 2.4	2	123	62	1	G	A
rs7253363	BC039523	active	0,298	0,734				χ2 = 1.1	1	180	7	0	G	T
rs13387792	MYT1L	mAIL	0,299	0,734				χ2 = 1.1	1	164	23	0	G	A
rs3014866	S100A9	mAIL	0,300	0,734				χ2 = 2.4	2	54	95	39	T	C
rs6198	NR3C1	remyel	0,301	0,734				χ2 = 2.4	2	118	54	9	T	C
rs2236851	RUNX3	MMAS	0,304	0,734				χ2 = 2.4	2	126	53	7	C	T
rs404694	MAF	Log_Lesion_load_BRS	0,305	0,734				F = 1.2	2/151	92	79	14	A	C
rs9480865	FOXO3	remyel	0,306	0,734				χ2 = 2.4	2	142	39	5	T	C
rs12644284	TRIM2	MMAS	0,306	0,734				χ2 = 2.4	2	103	69	13	A	G
rs4953911	MGAT5	Grey_matter_lesions	0,307	0,734				χ2 = 2.4	2	103	74	8	A	T
rs156429	GPNMB	Log_Lesion_load_BRS	0,310	0,738				F = 1.2	2/151	62	91	32	T	C
rs10516537	DKK2	Log_Lesion_load_BRS	0,313	0,740				F = 1.2	2/150	132	45	7	C	A
rs17541777	PTGER3	Log_Lesion_load_BRS	0,315	0,740				F = 1.2	2/149	145	34	2	T	C
rs12644284	TRIM2	mAIL	0,317	0,740				χ2 = 2.3	2	103	69	13	A	G
rs3014866	S100A9	remyel	0,318	0,740				χ2 = 2.3	2	54	95	39	T	C
rs2853744	SPP1	Log_Lesion_load_BRS	0,319	0,740				F = 1.2	2/153	171	15	2	G	T
rs9892479	ASIC2	Log_Lesion_load_BRS	0,319	0,740				F = 1.2	2/153	165	22	1	G	T
rs6994992	NRG1	remyel	0,322	0,740				χ2 = 2.3	2	70	90	28	C	T
rs2853744	SPP1	Grey_matter_lesions	0,324	0,740				χ2 = 2.3	2	171	15	2	G	T
rs9319189	SLITRK6	Grey_matter_lesions	0,325	0,740				χ2 = 2.3	2	92	76	13	G	A
rs2766051	DOCK1	Log_Lesion_load_BRS	0,325	0,740				F = 1.1	2/149	145	34	4	G	A
rs41423247	NR3C1	MMAS	0,325	0,740				χ2 = 2.2	2	88	65	31	G	C
rs11652878	ITGAE	Log_Lesion_load_BRS	0,325	0,740				F = 1.1	2/151	160	25	1	A	G
rs1318	PITPNC1	remyel	0,328	0,740				χ2 = 2.2	2	117	60	5	A	G
rs2796267	CD46	MMAS	0,329	0,740				χ2 = 2.2	2	54	86	36	A	G
rs1448239	GRIN2A	mAIL	0,330	0,740				χ2 = 2.2	2	130	53	1	G	C
rs3014866	S100A9	Grey_matter_lesions	0,331	0,740				χ2 = 2.2	2	54	95	39	T	C
rs10977017	PTPRD	mAIL	0,335	0,740				χ2 = 2.2	2	124	52	5	G	A
rs305124	UBE2K	Log_Lesion_load_BRS	0,335	0,740				F = 1.1	2/150	151	33	1	A	G
rs9808753	IFNGR2	mAIL	0,336	0,740				χ2 = 2.2	2	134	48	6	A	G
rs423904	IL1RN	Log_Lesion_load_BRS	0,336	0,740				F = 1.1	2/146	91	80	8	C	T
rs1318	PITPNC1	Grey_matter_lesions	0,337	0,740				χ2 = 2.2	2	117	60	5	A	G
rs404694	MAF	MMAS	0,337	0,740				χ2 = 2.2	2	92	79	14	A	C

rs3130253	MOG	mAIL	0,344	0,752				χ2 = 2.1	2	134	45	4	G	A
rs1761667	CD36	Grey_matter_lesions	0,350	0,761				χ2 = 2.1	2	58	85	39	A	G
rs2037815	CASP8	mAIL	0,351	0,761				χ2 = 2.1	2	46	99	42	A	G
rs10078091	CDH10	remyel	0,352	0,761				χ2 = 2.1	2	95	76	14	G	A
rs17505688	NTNG1	MMAS	0,359	0,767				χ2 = 2.1	2	154	26	2	T	C
rs11765693	YWHAG	MMAS	0,360	0,767				χ2 = 2.0	2	81	87	13	A	G
rs404694	MAF	Grey_matter_lesions	0,361	0,767				χ2 = 2.0	2	92	79	14	A	C
rs9480865	FOXO3	active	0,366	0,767				χ2 = 2.0	2	142	39	5	T	C
rs2569190	CD14	mAIL	0,367	0,767				χ2 = 2.0	2	43	96	39	G	A
rs744166	STAT3	MMAS	0,368	0,767				χ2 = 2.0	2	63	90	30	T	C
rs2028455	SKA1	mAIL	0,369	0,767				χ2 = 2.0	2	82	84	19	C	T
rs4747075	ADAMTS14	Grey_matter_lesions	0,370	0,767				χ2 = 2.0	2	86	58	30	G	A
rs11666377	CPAMD8	remyel	0,370	0,767				χ2 = 2.0	2	134	52	3	C	T
rs11957313	KCNIP1	mAIL	0,370	0,767				χ2 = 2.0	2	132	49	6	G	A
rs1800682	FAS,ACTA2	active	0,371	0,767				χ2 = 2.0	2	43	100	41	T	C
rs744166	STAT3	mAIL	0,371	0,767				χ2 = 2.0	2	63	90	30	T	C
rs11957313	KCNIP1	MMAS	0,375	0,767				χ2 = 2.0	2	132	49	6	G	A
rs4747075	ADAMTS14	Log_Lesion_load_BRS	0,376	0,767				F = 1.0	2/142	86	58	30	G	A
rs4819554	IL17RA	MMAS	0,376	0,767				χ2 = 2.0	2	145	38	4	A	G
rs716595	MXI1	mAIL	0,378	0,767				χ2 = 0.8	1	144	41	1	G	A
rs263153	LOC153910	active	0,379	0,767				χ2 = 0.8	1	148	37	0	G	T
rs11957313	KCNIP1	Log_Lesion_load_BRS	0,381	0,767				F = 1.0	2/152	132	49	6	G	A
rs423904	IL1RN	MMAS	0,383	0,767				χ2 = 1.9	2	91	80	8	C	T
rs2069762	IL2	Log_Lesion_load_BRS	0,383	0,767				F = 1.0	2/151	110	65	11	T	G
rs2039485	NUBPL	Grey_matter_lesions	0,384	0,767				χ2 = 1.9	2	104	74	10	T	C
rs1137933	NOS2	MMAS	0,384	0,767				χ2 = 1.9	2	115	65	8	G	A
rs72928038	BACH2	Grey_matter_lesions	0,384	0,767				χ2 = 1.9	2	119	67	2	G	A
rs6941421	JARID2	active	0,386	0,767				χ2 = 1.9	2	62	95	28	T	C
rs6899560	FUT9	mAIL	0,386	0,767				χ2 = 0.8	1	170	18	0	A	G
rs79877597	IL17A	remyel	0,387	0,767				χ2 = 1.9	2	127	53	6	C	A
rs333	CCR5	active	0,391	0,772				χ2 = 1.9	2	154	26	5	AGAATTTCCAGACA	-
rs17505688	NTNG1	active	0,395	0,778				χ2 = 1.9	2	154	26	2	T	C
rs716595	MXI1	Log_Lesion_load_BRS	0,398	0,780				F = 0.7	1/152	144	41	1	G	A
rs3865444	CD33	mAIL	0,403	0,786				χ2 = 1.8	2	86	75	28	G	T
rs1800682	FAS,ACTA2	Grey_matter_lesions	0,403	0,786				χ2 = 1.8	2	43	100	41	T	C
rs2028455	SKA1	MMAS	0,405	0,786				χ2 = 1.8	2	82	84	19	C	T
rs6198	NR3C1	Log_Lesion_load_BRS	0,407	0,786				F = 0.9	2/149	118	54	9	T	C
rs11652878	ITGAE	MMAS	0,407	0,786				χ2 = 1.8	2	160	25	1	A	G
rs231775	CTLA4	mAIL	0,411	0,786				χ2 = 1.8	2	73	95	21	A	G
rs2766051	DOCK1	Grey_matter_lesions	0,412	0,786				χ2 = 1.8	2	145	34	4	G	A
rs4953911	MGAT5	active	0,412	0,786				χ2 = 1.8	2	103	74	8	A	T
rs13019537	LHCGR	Log_Lesion_load_BRS	0,413	0,786				F = 0.9	2/153	126	57	4	C	G
rs13067869	NLGN1	Log_Lesion_load_BRS	0,415	0,786				F = 0.9	2/147	147	30	2	T	G
rs9480865	FOXO3	mAIL	0,416	0,786				χ2 = 1.8	2	142	39	5	T	C
rs1386330	RAB38	Grey_matter_lesions	0,416	0,786				χ2 = 1.8	2	142	39	1	T	C
rs7134248	KDM2B	Log_Lesion_load_BRS	0,417	0,786				F = 0.9	2/151	54	93	39	C	T
rs11719646	ERC2	Grey_matter_lesions	0,423	0,788				χ2 = 1.7	2	61	99	23	A	G
rs1761667	CD36	MMAS	0,424	0,788				χ2 = 1.7	2	58	85	39	A	G
rs7134248	KDM2B	mAIL	0,427	0,788				χ2 = 1.7	2	54	93	39	C	T
rs12111597	NPSR1,NPSR1-AS1	remyel	0,434	0,788				χ2 = 1.7	2	99	73	10	G	A
rs3212227	IL12B	Log_Lesion_load_BRS	0,434	0,788				F = 0.8	2/144	125	44	7	A	C
rs1869410	SOX11	mAIL	0,435	0,788				χ2 = 1.7	2	88	71	19	T	C
rs299175	NLRP11	remyel	0,436	0,788				χ2 = 1.7	2	53	87	39	C	T
rs333	CCR5	Grey_matter_lesions	0,437	0,788				χ2 = 1.7	2	154	26	5	AGAATTTCCAGACA	-
rs11750073	CTNND2	remyel	0,438	0,788				χ2 = 1.7	2	122	55	7	C	T
rs2107538	CCL5	MMAS	0,438	0,788				χ2 = 1.6	2	119	59	5	C	T
rs12111597	NPSR1,NPSR1-AS1	MMAS	0,439	0,788				χ2 = 1.6	2	99	73	10	G	A
rs3814022	MGAT5	Log_Lesion_load_BRS	0,439	0,788				F = 0.8	2/150	114	64	6	C	G
rs299175	NLRP11	MMAS	0,440	0,788				χ2 = 1.6	2	53	87	39	C	T
rs2039485	NUBPL	Log_Lesion_load_BRS	0,440	0,788				F = 0.8	2/153	104	74	10	T	C
rs744166	STAT3	remyel	0,440	0,788				χ2 = 1.6	2	63	90	30	T	C
rs1869410	SOX11	remyel	0,441	0,788				χ2 = 1.6	2	88	71	19	T	C
rs6899560	FUT9	MMAS	0,443	0,788				χ2 = 0.6	1	170	18	0	A	G
rs231775	CTLA4	Log_Lesion_load_BRS	0,443	0,788				F = 0.8	2/154	73	95	21	A	G
rs6941421	JARID2	Log_Lesion_load_BRS	0,444	0,788				F = 0.8	2/152	62	95	28	T	C
rs3814022	MGAT5	remyel	0,444	0,788				χ2 = 1.6	2	114	64	6	C	G
rs10078091	CDH10	Grey_matter_lesions	0,447	0,789				χ2 = 1.6	2	95	76	14	G	A
rs6941421	JARID2	remyel	0,447	0,789				χ2 = 1.6	2	62	95	28	T	C
rs10505082	ZFPM2	Grey_matter_lesions	0,450	0,790				χ2 = 0.6	1	123	62	1	G	A
rs1761667	CD36	mAIL	0,450	0,790				χ2 = 1.6	2	58	85	39	A	G
rs1883832	CD40	mAIL	0,458	0,801				χ2 = 1.6	2	109	68	11	C	T
rs9319189	SLITRK6	Log_Lesion_load_BRS	0,460	0,802				F = 0.8	2/148	92	76	13	G	A
rs7914524	AK124226	mAIL	0,463	0,804				χ2 = 1.5	2	110	62	10	C	T
rs2236851	RUNX3	remyel	0,464	0,804				χ2 = 1.5	2	126	53	7	C	T
rs2853744	SPP1	mAIL	0,465	0,804				χ2 = 1.5	2	171	15	2	G	T
rs423904	IL1RN	mAIL	0,468	0,806				χ2 = 1.5	2	91	80	8	C	T
rs261902	BICD1	active	0,471	0,810				χ2 = 1.5	2	114	57	8	C	T
rs305124	UBE2K	Grey_matter_lesions	0,476	0,815				χ2 = 1.5	2	151	33	1	A	G
rs16925027	KDM4C	active	0,478	0,816				χ2 = 1.5	2	111	62	8	A	G
rs7253363	BC039523	remyel	0,479	0,816				χ2 = 0.5	1	180	7	0	G	T
rs3865444	CD33	active	0,481	0,816				χ2 = 1.5	2	86	75	28	G	T
rs41423247	NR3C1	active	0,482	0,816				χ2 = 1.5	2	88	65	31	G	C
rs868824	IMMP2L	mAIL	0,483	0,816				χ2 = 1.5	2	62	81	40	T	C
rs404694	MAF	remyel	0,489	0,824				χ2 = 1.4	2	92	79	14	A	C
rs12127450	AGBL4	Grey_matter_lesions	0,491	0,826				χ2 = 1.4	2	133	47	3	T	C
rs1799987	CCR5	Log_Lesion_load_BRS	0,494	0,826				F = 0.7	2/151	55	88	43	A	G
rs755622	MIF-AS1	mAIL	0,494	0,826				χ2 = 1.4	2	119	63	3	C	G
rs2074897	NDUFS7	Log_Lesion_load_BRS	0,496	0,828				F = 0.7	2/147	54	94	33	G	A
rs2028455	SKA1	Log_Lesion_load_BRS	0,500	0,831				F = 0.7	2/151	82	84	19	C	T
rs1800682	FAS,ACTA2	mAIL	0,508	0,837				χ2 = 1.4	2	43	100	41	T	C
rs5673	PTGER3	mAIL	0,509	0,837				χ2 = 0.4	1	169	17	0	A	T
rs2069762	IL2	active	0,509	0,837				χ2 = 1.4	2	110	65	11	T	G
rs2069727	IFNG	MMAS	0,509	0,837				χ2 = 1.3	2	45	92	43	A	G
rs8056098	CLEC16A	Grey_matter_lesions	0,512	0,837				χ2 = 1.3	2	75	89	20	G	A
rs4747075	ADAMTS14	active	0,513	0,837				χ2 = 1.3	2	86	58	30	G	A

rs13387792	MYT1L	remyel	0,515	0,837				χ2 = 0.4	1	164	23	0	G	A
rs4953911	MGAT5	MMAS	0,516	0,837				χ2 = 1.3	2	103	74	8	A	T
rs6899560	FUT9	active	0,517	0,837				χ2 = 0.4	1	170	18	0	A	G
rs2069762	IL2	remyel	0,517	0,837				χ2 = 1.3	2	110	65	11	T	G
rs1869410	SOX11	Log_Lesion_load_BRS	0,519	0,837				F = 0.7	2/145	88	71	19	T	C
rs7914524	AK124226	remyel	0,520	0,837				χ2 = 1.3	2	110	62	10	C	T
rs17505688	NTNG1	Log_Lesion_load_BRS	0,526	0,844				F = 0.6	2/151	154	26	2	T	C
rs11652878	ITGAE	remyel	0,527	0,844				χ2 = 1.3	2	160	25	1	A	G
rs16925027	KDM4C	MMAS	0,532	0,848				χ2 = 1.3	2	111	62	8	A	G
rs10178552	ASXL2	remyel	0,533	0,848				χ2 = 1.3	2	73	87	25	C	T
rs1761667	CD36	Log_Lesion_load_BRS	0,535	0,848				F = 0.6	2/148	58	85	39	A	G
rs13067869	NLGN1	remyel	0,537	0,848				χ2 = 1.2	2	147	30	2	T	G
rs12127450	AGBL4	Log_Lesion_load_BRS	0,538	0,848				F = 0.6	2/149	133	47	3	T	C
rs755622	MIF-AS1	Grey_matter_lesions	0,538	0,848				χ2 = 1.2	2	119	63	3	C	G
rs3814022	MGAT5	active	0,539	0,848				χ2 = 1.2	2	114	64	6	C	G
rs231775	CTLA4	Grey_matter_lesions	0,543	0,851				χ2 = 1.2	2	73	95	21	A	G
rs752092	CHSY1	Log_Lesion_load_BRS	0,545	0,851				F = 0.6	2/155	85	81	24	T	C
rs3116496	CD28	mAIL	0,545	0,851				χ2 = 1.2	2	134	45	4	T	C
rs11765693	YWHAG	active	0,549	0,855				χ2 = 1.2	2	81	87	13	A	G
rs11750073	CTNND2	MMAS	0,551	0,855				χ2 = 1.2	2	122	55	7	C	T
rs3116496	CD28	active	0,552	0,855				χ2 = 1.2	2	134	45	4	T	C
rs41423247	NR3C1	Grey_matter_lesions	0,553	0,855				χ2 = 1.2	2	88	65	31	G	C
rs9892479	ASIC2	active	0,556	0,856				χ2 = 1.2	2	165	22	1	G	T
rs2234978	FAS	Grey_matter_lesions	0,560	0,856				χ2 = 1.2	2	81	83	22	C	T
rs2236851	RUNX3	Log_Lesion_load_BRS	0,561	0,856				F = 0.6	2/151	126	53	7	C	T
rs5673	PTGER3	Log_Lesion_load_BRS	0,561	0,856				F = 0.3	1/152	169	17	0	A	T
rs3014866	S100A9	active	0,562	0,856				χ2 = 1.2	2	54	95	39	T	C
rs2399849	CAMK1D	Log_Lesion_load_BRS	0,564	0,856				F = 0.6	2/148	134	46	2	G	A
rs10505082	ZFPM2	MMAS	0,564	0,856				χ2 = 1.1	2	123	62	1	G	A
rs1883832	CD40	Log_Lesion_load_BRS	0,567	0,857				F = 0.6	2/153	109	68	11	C	T
rs1800682	FAS,ACTA2	remyel	0,567	0,857				χ2 = 1.1	2	43	100	41	T	C
rs11666377	CPAMD8	Log_Lesion_load_BRS	0,571	0,861				F = 0.6	2/154	134	52	3	C	T
rs12644284	TRIM2	Grey_matter_lesions	0,572	0,861				χ2 = 1.1	2	103	69	13	A	G
rs2074897	NDUFS7	MMAS	0,578	0,861				χ2 = 1.1	2	54	94	33	G	A
rs752092	CHSY1	active	0,579	0,861				χ2 = 1.1	2	85	81	24	T	C
rs156429	GPNMB	mAIL	0,580	0,861				χ2 = 1.1	2	62	91	32	T	C
rs716595	MXI1	MMAS	0,581	0,861				χ2 = 0.3	1	144	41	1	G	A
rs12127450	AGBL4	active	0,582	0,861				χ2 = 1.1	2	133	47	3	T	C
rs2107538	CCL5	mAIL	0,583	0,861				χ2 = 1.1	2	119	59	5	C	T
rs17505688	NTNG1	Grey_matter_lesions	0,583	0,861				χ2 = 1.1	2	154	26	2	T	C
rs13387792	MYT1L	MMAS	0,584	0,861				χ2 = 0.3	1	164	23	0	G	A
rs12111597	NPSR1,NPSR1-AS1	active	0,590	0,865				χ2 = 1.1	2	99	73	10	G	A
rs263153	LOC153910	mAIL	0,590	0,865				χ2 = 0.3	1	148	37	0	G	T
rs1927457	SVIL	Grey_matter_lesions	0,592	0,865				χ2 = 1.0	2	79	84	14	T	C
rs6190	NR3C1	Log_Lesion_load_BRS	0,595	0,865				F = 0.5	2/152	166	20	1	G	A
rs9892479	ASIC2	Grey_matter_lesions	0,597	0,865				χ2 = 1.0	2	165	22	1	G	T
rs11750073	CTNND2	active	0,598	0,865				χ2 = 1.0	2	122	55	7	C	T
rs6899560	FUT9	remyel	0,598	0,865				χ2 = 0.3	1	170	18	0	A	G
rs7253363	BC039523	Log_Lesion_load_BRS	0,601	0,865				F = 0.3	1/153	180	7	0	G	T
rs156429	GPNMB	MMAS	0,608	0,865				χ2 = 1.0	2	62	91	32	T	C
rs7134248	KDM2B	active	0,609	0,865				χ2 = 1.0	2	54	93	39	C	T
rs6899560	FUT9	Log_Lesion_load_BRS	0,610	0,865				F = 0.3	1/154	170	18	0	A	G
rs404694	MAF	mAIL	0,611	0,865				χ2 = 1.0	2	92	79	14	A	C
rs156429	GPNMB	remyel	0,612	0,865				χ2 = 1.0	2	62	91	32	T	C
rs1137933	NOS2	active	0,614	0,865				χ2 = 1.0	2	115	65	8	G	A
rs10516537	DKK2	remyel	0,615	0,865				χ2 = 1.0	2	132	45	7	C	A
rs5673	PTGER3	active	0,617	0,865				χ2 = 0.3	1	169	17	0	A	T
rs755622	MIF-AS1	remyel	0,617	0,865				χ2 = 1.0	2	119	63	3	C	G
rs17541777	PTGER3	mAIL	0,618	0,865				χ2 = 1.0	2	145	34	2	T	C
rs17541777	PTGER3	remyel	0,618	0,865				χ2 = 1.0	2	145	34	2	T	C
rs17398267	LHCGR	remyel	0,620	0,865				χ2 = 1.0	2	112	58	14	T	G
rs4880213	GRIN1	Log_Lesion_load_BRS	0,620	0,865				F = 0.5	2/151	63	99	24	C	T
rs7744583	ARID1B	active	0,621	0,865				χ2 = 1.0	2	78	85	20	G	A
rs8056098	CLEC16A	MMAS	0,622	0,865				χ2 = 1.0	2	75	89	20	G	A
rs752092	CHSY1	remyel	0,622	0,865				χ2 = 0.9	2	85	81	24	T	C
rs744166	STAT3	Grey_matter_lesions	0,623	0,865				χ2 = 0.9	2	63	90	30	T	C
rs5673	PTGER3	MMAS	0,626	0,865				χ2 = 0.2	1	169	17	0	A	T
rs6198	NR3C1	active	0,626	0,865				χ2 = 0.9	2	118	54	9	T	C
rs261902	BICD1	remyel	0,626	0,865				χ2 = 0.9	2	114	57	8	C	T
rs6941421	JARID2	Grey_matter_lesions	0,635	0,876				χ2 = 0.9	2	62	95	28	T	C
rs1799987	CCR5	MMAS	0,637	0,876				χ2 = 0.9	2	55	88	43	A	G
rs2399849	CAMK1D	Grey_matter_lesions	0,639	0,876				χ2 = 0.9	2	134	46	2	G	A
rs1064395	NCAN	Log_Lesion_load_BRS	0,641	0,876				F = 0.4	2/149	132	47	5	G	A
rs1386330	RAB38	mAIL	0,642	0,876				χ2 = 0.9	2	142	39	1	T	C
rs2039485	NUBPL	MMAS	0,643	0,876				χ2 = 0.9	2	104	74	10	T	C
rs11666377	CPAMD8	Grey_matter_lesions	0,646	0,876				χ2 = 0.9	2	134	52	3	C	T
rs13067869	NLGN1	Grey_matter_lesions	0,647	0,876				χ2 = 0.9	2	147	30	2	T	G
rs9319189	SLITRK6	remyel	0,647	0,876				χ2 = 0.9	2	92	76	13	G	A
rs752092	CHSY1	MMAS	0,649	0,876				χ2 = 0.9	2	85	81	24	T	C
rs9808753	IFNGR2	Grey_matter_lesions	0,651	0,878				χ2 = 0.9	2	134	48	6	A	G
rs3116496	CD28	MMAS	0,652	0,878				χ2 = 0.9	2	134	45	4	T	C
rs8056098	CLEC16A	active	0,657	0,882				χ2 = 0.8	2	75	89	20	G	A
rs1799987	CCR5	Grey_matter_lesions	0,665	0,888				χ2 = 0.8	2	55	88	43	A	G
rs9808753	IFNGR2	active	0,666	0,888				χ2 = 0.8	2	134	48	6	A	G
rs1597944	CHRND	Log_Lesion_load_BRS	0,668	0,888				F = 0.4	2/152	56	87	43	T	C
rs868824	IMMP2L	MMAS	0,671	0,888				χ2 = 0.8	2	62	81	40	T	C
rs10052957	NR3C1	Grey_matter_lesions	0,671	0,888				χ2 = 0.8	2	79	86	22	G	A
rs1448239	GRIN2A	Grey_matter_lesions	0,672	0,888				χ2 = 0.8	2	130	53	1	G	C
rs3814022	MGAT5	Grey_matter_lesions	0,672	0,888				χ2 = 0.8	2	114	64	6	C	G
rs10178552	ASXL2	MMAS	0,673	0,888				χ2 = 0.8	2	73	87	25	C	T
rs2766051	DOCK1	active	0,678	0,891				χ2 = 0.8	2	145	34	4	G	A
rs5742909	CTLA4	MMAS	0,682	0,891				χ2 = 0.8	2	154	27	4	C	T
rs6198	NR3C1	Grey_matter_lesions	0,684	0,891				χ2 = 0.8	2	118	54	9	T	C
rs5673	PTGER3	Grey_matter_lesions	0,684	0,891				χ2 = 0.2	1	169	17	0	A	T
rs716595	MXI1	Grey_matter_lesions	0,685	0,891				χ2 = 0.2	1	144	41	1	G	A

rs337718	CBLN2	Log_Lesion_load_BRS	0,685	0,891				F = 0.4	2/150	91	78	15	C	T
rs12111597	NPSR1,NPSR1-AS1	Log_Lesion_load_BRS	0,686	0,891				F = 0.4	2/148	99	73	10	G	A
rs2569190	CD14	Log_Lesion_load_BRS	0,688	0,892				F = 0.4	2/148	43	96	39	G	A
rs3212227	IL12B	remyel	0,689	0,892				χ2 = 0.7	2	125	44	7	A	C
rs9480865	FOXO3	Log_Lesion_load_BRS	0,694	0,892				F = 0.4	2/151	142	39	5	T	C
rs8192678	PPARGC1A	Log_Lesion_load_BRS	0,695	0,892				F = 0.4	2/150	90	77	17	G	A
rs7134248	KDM2B	MMAS	0,695	0,892				χ2 = 0.7	2	54	93	39	C	T
rs1064395	NCAN	MMAS	0,698	0,892				χ2 = 0.7	2	132	47	5	G	A
rs7211577	COX10	active	0,698	0,892				χ2 = 0.7	2	57	96	34	G	A
rs2236851	RUNX3	Grey_matter_lesions	0,700	0,892				χ2 = 0.7	2	126	53	7	C	T
rs716595	MXI1	active	0,701	0,892				χ2 = 0.1	1	144	41	1	G	A
rs4819554	IL17RA	mAIL	0,701	0,892				χ2 = 0.7	2	145	38	4	A	G
rs17157903	RELN	mAIL	0,705	0,894				χ2 = 0.7	2	150	34	3	C	T
rs7134248	KDM2B	Grey_matter_lesions	0,706	0,894				χ2 = 0.7	2	54	93	39	C	T
rs9892479	ASIC2	MMAS	0,708	0,894				χ2 = 0.7	2	165	22	1	G	T
rs2796267	CD46	Grey_matter_lesions	0,708	0,894				χ2 = 0.7	2	54	86	36	A	G
rs1927457	SVIL	active	0,712	0,895				χ2 = 0.7	2	79	84	14	T	C
rs10178552	ASXL2	Grey_matter_lesions	0,712	0,895				χ2 = 0.7	2	73	87	25	C	T
rs1061170	CFH	remyel	0,714	0,895				χ2 = 0.7	2	70	84	24	T	C
rs2853744	SPP1	MMAS	0,722	0,902				χ2 = 0.7	2	171	15	2	G	T
rs10078091	CDH10	Log_Lesion_load_BRS	0,723	0,902				F = 0.3	2/151	95	76	14	G	A
rs3130253	MOG	Grey_matter_lesions	0,727	0,906				χ2 = 0.6	2	134	45	4	G	A
rs1061170	CFH	Grey_matter_lesions	0,730	0,907				χ2 = 0.6	2	70	84	24	T	C
rs10516537	DKK2	mAIL	0,733	0,907				χ2 = 0.6	2	132	45	7	C	A
rs4953911	MGAT5	mAIL	0,733	0,907				χ2 = 0.6	2	103	74	8	A	T
rs2236851	RUNX3	mAIL	0,735	0,907				χ2 = 0.6	2	126	53	7	C	T
rs13387792	MYT1L	active	0,736	0,907				χ2 = 0.1	1	164	23	0	G	A
rs6190	NR3C1	active	0,737	0,907				χ2 = 0.6	2	166	20	1	G	A
rs261902	BICD1	Log_Lesion_load_BRS	0,746	0,916				F = 0.3	2/147	114	57	8	C	T
rs2028455	SKA1	active	0,747	0,916				χ2 = 0.6	2	82	84	19	C	T
rs7914524	AK124226	Log_Lesion_load_BRS	0,749	0,917				F = 0.3	2/149	110	62	10	C	T
rs2039485	NUBPL	mAIL	0,751	0,917				χ2 = 0.6	2	104	74	10	T	C
rs3014866	S100A9	MMAS	0,755	0,920				χ2 = 0.6	2	54	95	39	T	C
rs11750073	CTNND2	mAIL	0,756	0,920				χ2 = 0.6	2	122	55	7	C	T
rs2069762	IL2	Grey_matter_lesions	0,758	0,920				χ2 = 0.6	2	110	65	11	T	G
rs333	CCR5	Log_Lesion_load_BRS	0,763	0,925				F = 0.3	2/151	154	26	5	AGAATTTCCAGACA	-
rs11652878	ITGAE	active	0,765	0,926				χ2 = 0.5	2	160	25	1	A	G
rs2028455	SKA1	Grey_matter_lesions	0,768	0,927				χ2 = 0.5	2	82	84	19	C	T
rs2569190	CD14	active	0,770	0,927				χ2 = 0.5	2	43	96	39	G	A
rs10052957	NR3C1	active	0,771	0,927				χ2 = 0.5	2	79	86	22	G	A
rs11652878	ITGAE	Grey_matter_lesions	0,778	0,932				χ2 = 0.5	2	160	25	1	A	G
rs1869410	SOX11	Grey_matter_lesions	0,779	0,932				χ2 = 0.5	2	88	71	19	T	C
rs2028455	SKA1	remyel	0,779	0,932				χ2 = 0.5	2	82	84	19	C	T
rs10078091	CDH10	MMAS	0,783	0,934				χ2 = 0.5	2	95	76	14	G	A
rs231775	CTLA4	MMAS	0,785	0,934				χ2 = 0.5	2	73	95	21	A	G
rs9808753	IFNGR2	Log_Lesion_load_BRS	0,787	0,935				F = 0.2	2/153	134	48	6	A	G
rs1061170	CFH	active	0,793	0,936				χ2 = 0.5	2	70	84	24	T	C
rs299175	NLRP11	Log_Lesion_load_BRS	0,793	0,936				F = 0.2	2/148	53	87	39	C	T
rs11719646	ERC2	MMAS	0,793	0,936				χ2 = 0.5	2	61	99	23	A	G
rs13387792	MYT1L	Grey_matter_lesions	0,794	0,936				χ2 = 0.1	1	164	23	0	G	A
rs17541777	PTGER3	MMAS	0,802	0,943				χ2 = 0.4	2	145	34	2	T	C
rs79877597	IL17A	mAIL	0,805	0,945				χ2 = 0.4	2	127	53	6	C	A
rs12127450	AGBL4	MMAS	0,806	0,945				χ2 = 0.4	2	133	47	3	T	C
rs17541777	PTGER3	active	0,808	0,945				χ2 = 0.4	2	145	34	2	T	C
rs17541777	PTGER3	Grey_matter_lesions	0,811	0,945				χ2 = 0.4	2	145	34	2	T	C
rs10052957	NR3C1	MMAS	0,813	0,945				χ2 = 0.4	2	79	86	22	G	A
rs2796267	CD46	Log_Lesion_load_BRS	0,817	0,945				F = 0.2	2/145	54	86	36	A	G
rs868824	IMMP2L	remyel	0,818	0,945				χ2 = 0.4	2	62	81	40	T	C
rs7914524	AK124226	Grey_matter_lesions	0,818	0,945				χ2 = 0.4	2	110	62	10	C	T
rs755622	MIF-AS1	MMAS	0,821	0,945				χ2 = 0.4	2	119	63	3	C	G
rs868824	IMMP2L	Grey_matter_lesions	0,822	0,945				χ2 = 0.4	2	62	81	40	T	C
rs17157903	RELN	Grey_matter_lesions	0,825	0,945				χ2 = 0.4	2	150	34	3	C	T
rs156429	GPNMB	active	0,826	0,945				χ2 = 0.4	2	62	91	32	T	C
rs1597944	CHRND	MMAS	0,826	0,945				χ2 = 0.4	2	56	87	43	T	C
rs8192678	PPARGC1A	Grey_matter_lesions	0,831	0,945				χ2 = 0.4	2	90	77	17	G	A
rs13019537	LHCGR	Grey_matter_lesions	0,831	0,945				χ2 = 0.4	2	126	57	4	C	G
rs17157903	RELN	Log_Lesion_load_BRS	0,831	0,945				F = 0.2	2/152	150	34	3	C	T
rs299175	NLRP11	Grey_matter_lesions	0,831	0,945				χ2 = 0.4	2	53	87	39	C	T
rs9319189	SLITRK6	MMAS	0,832	0,945				χ2 = 0.4	2	92	76	13	G	A
rs333	CCR5	remyel	0,834	0,945				χ2 = 0.4	2	154	26	5	AGAATTTCCAGACA	-
rs4880213	GRIN1	mAIL	0,835	0,945				χ2 = 0.4	2	63	99	24	C	T
rs3814022	MGAT5	MMAS	0,839	0,945				χ2 = 0.4	2	114	64	6	C	G
rs7211577	COX10	remyel	0,840	0,945				χ2 = 0.3	2	57	96	34	G	A
rs333	CCR5	mAIL	0,842	0,945				χ2 = 0.3	2	154	26	5	AGAATTTCCAGACA	-
rs2037815	CASP8	MMAS	0,843	0,945				χ2 = 0.3	2	46	99	42	A	G
rs305124	UBE2K	MMAS	0,844	0,945				χ2 = 0.3	2	151	33	1	A	G
rs6190	NR3C1	mAIL	0,846	0,945				χ2 = 0.3	2	166	20	1	G	A
rs1597944	CHRND	Grey_matter_lesions	0,848	0,945				χ2 = 0.3	2	56	87	43	T	C
rs16925027	KDM4C	mAIL	0,852	0,945				χ2 = 0.3	2	111	62	8	A	G
rs11750073	CTNND2	Log_Lesion_load_BRS	0,852	0,945				F = 0.2	2/150	122	55	7	C	T
rs10243024	MET	Log_Lesion_load_BRS	0,852	0,945				F = 0.2	2/149	119	54	9	G	A
rs2037815	CASP8	Log_Lesion_load_BRS	0,853	0,945				F = 0.2	2/152	46	99	42	A	G
rs2569190	CD14	MMAS	0,854	0,945				χ2 = 0.3	2	43	96	39	G	A
rs2069762	IL2	MMAS	0,855	0,945				χ2 = 0.3	2	110	65	11	T	G
rs2037815	CASP8	remyel	0,857	0,945				χ2 = 0.3	2	46	99	42	A	G
rs1800682	FAS,ACTA2	Log_Lesion_load_BRS	0,857	0,945				F = 0.2	2/151	43	100	41	T	C
rs1448239	GRIN2A	MMAS	0,860	0,946				χ2 = 0.3	2	130	53	1	G	C
rs1137933	NOS2	Log_Lesion_load_BRS	0,862	0,947				F = 0.1	2/153	115	65	8	G	A
rs79877597	IL17A	active	0,868	0,950				χ2 = 0.3	2	127	53	6	C	A
rs11957313	KCNIP1	Grey_matter_lesions	0,870	0,950				χ2 = 0.3	2	132	49	6	G	A
rs2069727	IFNG	Grey_matter_lesions	0,870	0,950				χ2 = 0.3	2	45	92	43	A	G
rs2069727	IFNG	mAIL	0,871	0,950				χ2 = 0.3	2	45	92	43	A	G
rs10977017	PTPRD	Log_Lesion_load_BRS	0,875	0,953				F = 0.1	2/149	124	52	5	G	A
rs3814022	MGAT5	mAIL	0,878	0,954				χ2 = 0.3	2	114	64	6	C	G
rs2039485	NUBPL	remyel	0,880	0,954				χ2 = 0.3	2	104	74	10	T	C

rs2116078	KCNB2	Grey_matter_lesions	0,882	0,955				χ2 = 0.3	2	49	93	38	G	T
rs3212227	IL12B	MMAS	0,883	0,955				χ2 = 0.2	2	125	44	7	A	C
rs744166	STAT3	Log_Lesion_load_BRS	0,887	0,957				F = 0.1	2/149	63	90	30	T	C
rs10178552	ASXL2	Log_Lesion_load_BRS	0,893	0,961				F = 0.1	2/150	73	87	25	C	T
rs10516537	DKK2	active	0,893	0,961				χ2 = 0.2	2	132	45	7	C	A
rs6198	NR3C1	MMAS	0,896	0,961				χ2 = 0.2	2	118	54	9	T	C
rs3014866	S100A9	Log_Lesion_load_BRS	0,898	0,961				F = 0.1	2/153	54	95	39	T	C
rs13019537	LHCGR	mAIL	0,898	0,961				χ2 = 0.2	2	126	57	4	C	G
rs7211577	COX10	Log_Lesion_load_BRS	0,900	0,961				F = 0.1	2/153	57	96	34	G	A
rs1137933	NOS2	remyel	0,902	0,962				χ2 = 0.2	2	115	65	8	G	A
rs3865444	CD33	MMAS	0,907	0,962				χ2 = 0.2	2	86	75	28	G	T
rs7744583	ARID1B	MMAS	0,908	0,962				χ2 = 0.2	2	78	85	20	G	A
rs12111597	NPSR1,NPSR1-AS1	Grey_matter_lesions	0,909	0,962				χ2 = 0.2	2	99	73	10	G	A
rs337718	CBLN2	MMAS	0,909	0,962				χ2 = 0.2	2	91	78	15	C	T
rs1883832	CD40	MMAS	0,911	0,963				χ2 = 0.2	2	109	68	11	C	T
rs12644284	TRIM2	Log_Lesion_load_BRS	0,915	0,963				F = 0.1	2/151	103	69	13	A	G
rs13019537	LHCGR	remyel	0,915	0,963				χ2 = 0.2	2	126	57	4	C	G
rs423904	IL1RN	remyel	0,916	0,963				χ2 = 0.2	2	91	80	8	C	T
rs1883832	CD40	Grey_matter_lesions	0,918	0,963				χ2 = 0.2	2	109	68	11	C	T
rs10178552	ASXL2	mAIL	0,925	0,970				χ2 = 0.2	2	73	87	25	C	T
rs17398267	LHCGR	Grey_matter_lesions	0,930	0,973				χ2 = 0.1	2	112	58	14	T	G
rs7744583	ARID1B	Grey_matter_lesions	0,933	0,975				χ2 = 0.1	2	78	85	20	G	A
rs9480865	FOXO3	MMAS	0,936	0,976				χ2 = 0.1	2	142	39	5	T	C
rs10505082	ZFPM2	Log_Lesion_load_BRS	0,941	0,980				F = 0.1	2/151	123	62	1	G	A
rs7253363	BC039523	MMAS	0,946	0,980				χ2 = 0.0	1	180	7	0	G	T
rs17157903	RELN	MMAS	0,946	0,980				χ2 = 0.1	2	150	34	3	C	T
rs2069762	IL2	mAIL	0,949	0,980				χ2 = 0.1	2	110	65	11	T	G
rs13019537	LHCGR	MMAS	0,950	0,980				χ2 = 0.1	2	126	57	4	C	G
rs9808753	IFNGR2	remyel	0,950	0,980				χ2 = 0.1	2	134	48	6	A	G
rs876493	PNMT	remyel	0,952	0,981				χ2 = 0.1	2	71	81	34	A	G
rs16925027	KDM4C	Log_Lesion_load_BRS	0,954	0,981				F = 0.0	2/149	111	62	8	A	G
rs7134248	KDM2B	remyel	0,958	0,983				χ2 = 0.1	2	54	93	39	C	T
rs7744583	ARID1B	Log_Lesion_load_BRS	0,960	0,983				F = 0.0	2/150	78	85	20	G	A
rs16925027	KDM4C	Grey_matter_lesions	0,961	0,983				χ2 = 0.1	2	111	62	8	A	G
rs9808753	IFNGR2	MMAS	0,968	0,989				χ2 = 0.1	2	134	48	6	A	G
rs8192678	PPARGC1A	MMAS	0,972	0,991				χ2 = 0.1	2	90	77	17	G	A
rs1133763	CCL8	active	0,975	0,991				χ2 = 0.1	2	124	58	3	A	C
rs1386330	RAB38	remyel	0,976	0,991				χ2 = 0.0	2	142	39	1	T	C
rs1064395	NCAN	active	0,977	0,991				χ2 = 0.0	2	132	47	5	G	A
rs10516537	DKK2	Grey_matter_lesions	0,979	0,991				χ2 = 0.0	2	132	45	7	C	A
rs1448239	GRIN2A	Log_Lesion_load_BRS	0,980	0,991				F = 0.0	2/150	130	53	1	G	C
rs337718	CBLN2	Grey_matter_lesions	0,989	0,998				χ2 = 0.0	2	91	78	15	C	T
rs1799987	CCR5	remyel	0,992	0,998				χ2 = 0.0	2	55	88	43	A	G
rs261902	BICD1	MMAS	0,992	0,998				χ2 = 0.0	2	114	57	8	C	T
rs1133763	CCL8	Log_Lesion_load_BRS	0,997	0,999				F = 0.0	2/151	124	58	3	A	C
rs6899560	FUT9	Grey_matter_lesions	0,997	0,999				χ2 = 0.0	1	170	18	0	A	G
rs72928038	BACH2	MMAS	0,997	0,999				χ2 = 0.0	2	119	67	2	G	A
rs755622	MIF-AS1	Log_Lesion_load_BRS	1,000	1,000				F = 0.0	2/150	119	63	3	C	G
