# Supplementary material for: Post‐mortem multiple sclerosis lesion pathology is influenced by single nucleotide polymorphisms
Source: Brain Pathol. 2019 Jul 23;30(1):106–19. doi: 10.1111/bpa.12760 (PMC6916567; doi:10.1111/bpa.12760)
Supplement: Supplementary file 9 — Figure S1. rs1064395 in males and females (PDF). [file BPA-30-106-s012.docx]

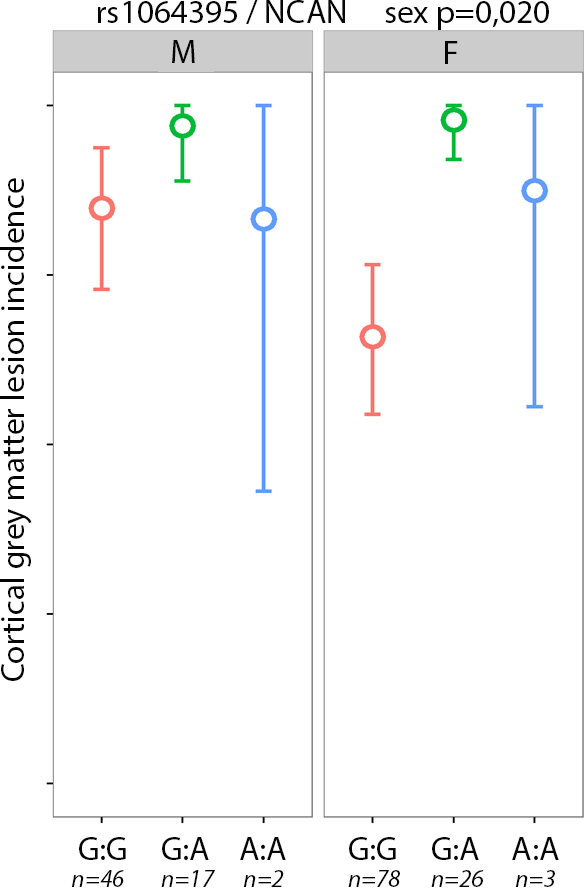


**Supplementary figure 1.** rs1064395 in males and females

Both sex and genotype of rs1064395 have an effect on incidence of cortical grey matter lesions. Males and carriers of the A allele for rs1064395 show a higher incidence of cortical grey matter lesions.
