# Supplementary material for: Post‐mortem multiple sclerosis lesion pathology is influenced by single nucleotide polymorphisms
Source: Brain Pathol. 2019 Jul 23;30(1):106–19. doi: 10.1111/bpa.12760 (PMC6916567; doi:10.1111/bpa.12760)

Supplementary figure 2. Single tissue eQTLs of significant SNPs and their SNPs in strong LD in brain tissues and EBV transformed cells (lymphocytes and fibroblasts)
a: rs2234978/FAS single tissue eQTLs. b: rs11957313/KCNIP1 single tissue eQTL c: rs1064395/NCAN single tissue eQTLs d: rs3130253/MOG single tissue eQTL

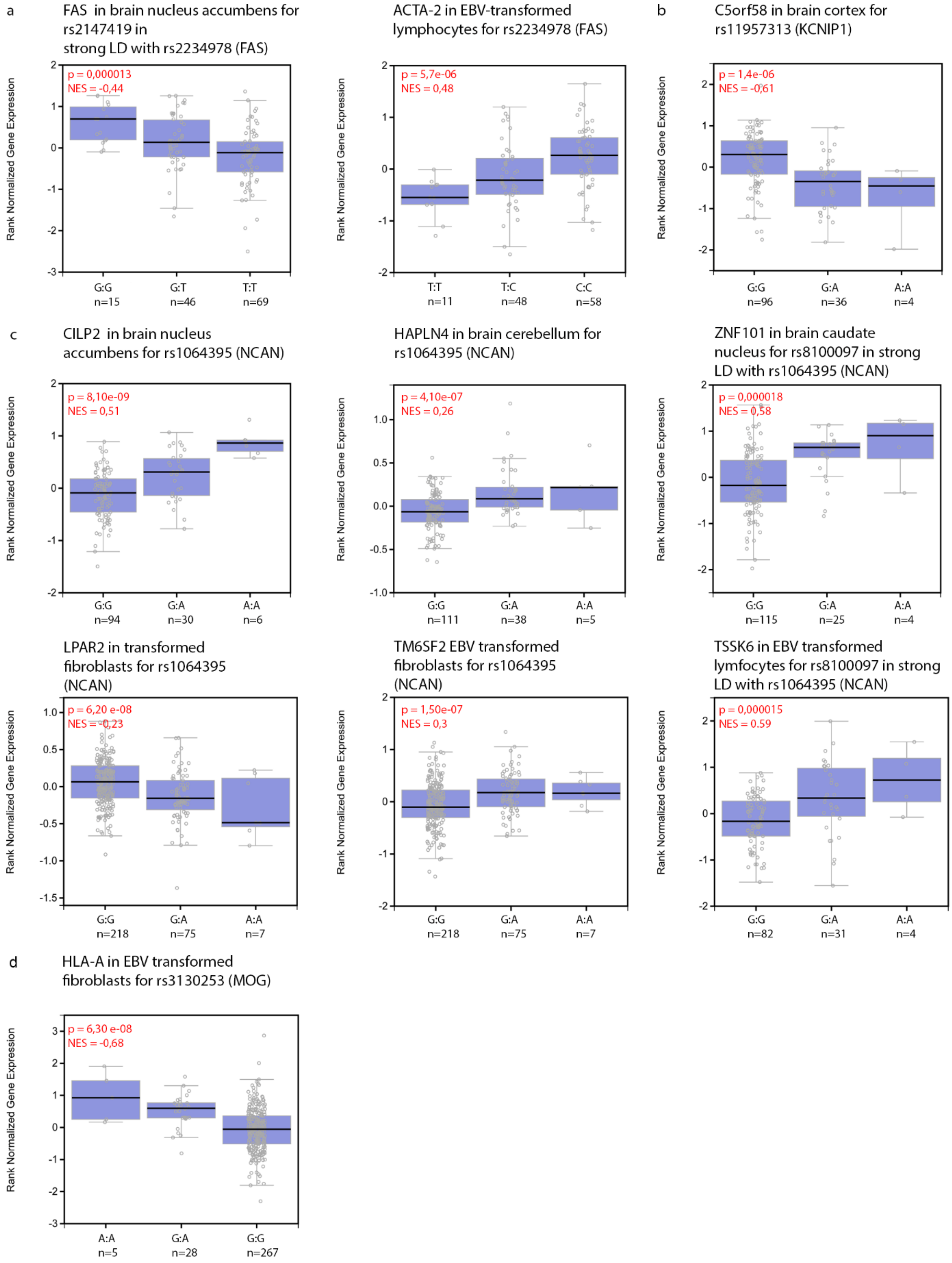

Supplement: Supplementary file 10 — Figure S2. Single tissue eQTLs of significant SNPs and their SNPs in strong LD in brain tissues and EBV transformed cells (lymphocytes and fibroblasts) (PDF). [file BPA-30-106-s013.pdf]
