# Supplementary material for: Post‐mortem multiple sclerosis lesion pathology is influenced by single nucleotide polymorphisms
Source: Brain Pathol. 2019 Jul 23;30(1):106–19. doi: 10.1111/bpa.12760 (PMC6916567; doi:10.1111/bpa.12760)
Supplement: Supplementary file 11 — Figure S3. rs2234978 and relative gene expression for FAS in subgroups (PDF). [file BPA-30-106-s011.docx]

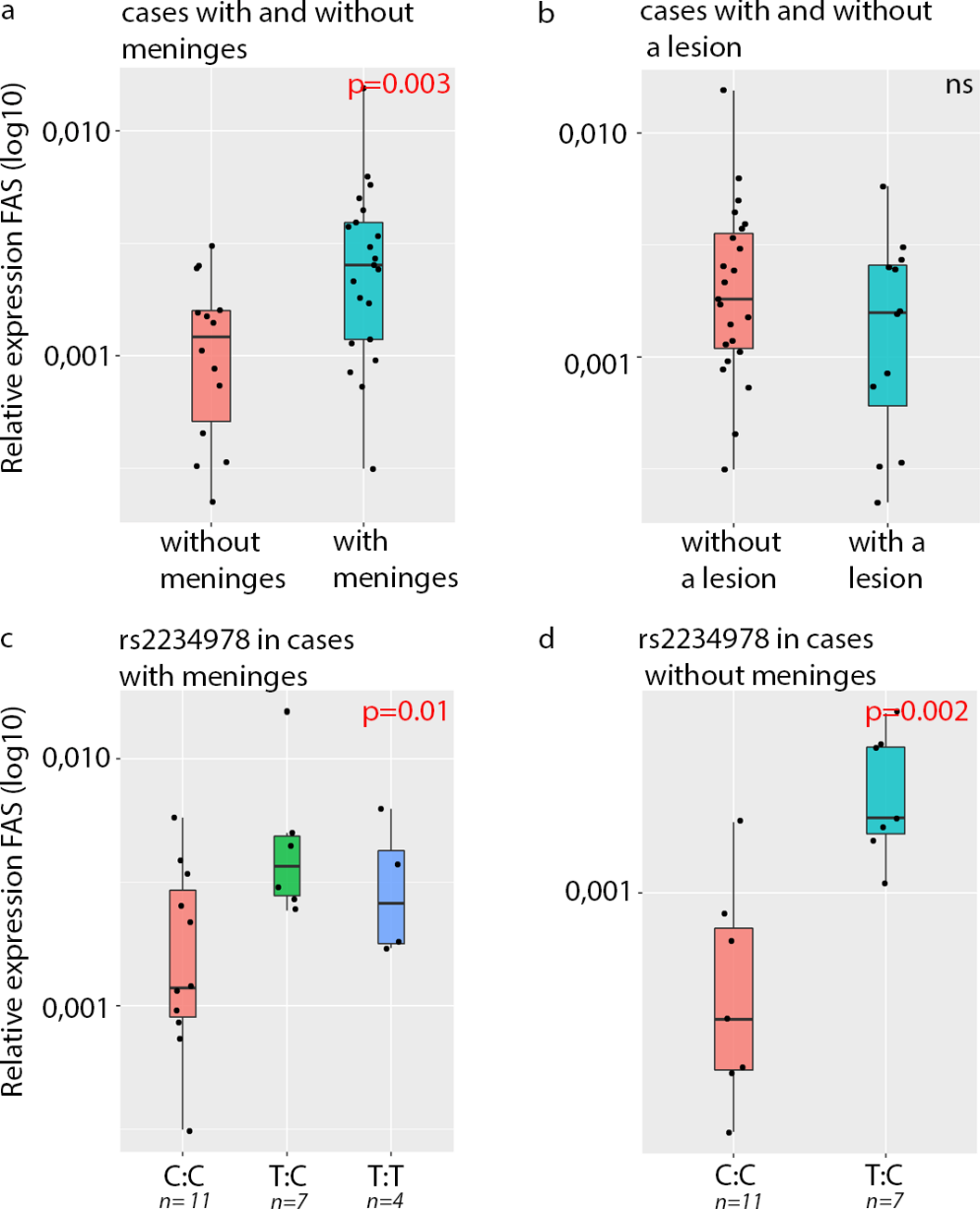


**Supplementary figure 3.** rs2234978 and relative gene expression for FAS in subgroups a: FAS expression is significantly higher in cases that had meninges in the section (n=22) compared to cases without meninges (n=18). b: there is no difference in FAS expression in cases without (n=25) and with (n=15) an MS lesion in the section. c: rs2234978 T allele is associated with increased FAS expression in cases with meninges in the section. d: rs2234978 T allele is associated with increased FAS expression in cases without meninges in the section.
