# Supplementary material for: Post‐mortem multiple sclerosis lesion pathology is influenced by single nucleotide polymorphisms
Source: Brain Pathol. 2019 Jul 23;30(1):106–19. doi: 10.1111/bpa.12760 (PMC6916567; doi:10.1111/bpa.12760)

Supplementary figure 4. Relative gene expression levels of eQTL genes that were not significantly related to the MS pathology-associated SNPs
a. Relative expression levels of genes that the SNPs are located in that were not significantly correlated with the genotype. b. Relative expression levels of the eQTL gene for rs11957313. c. Relative expression levels of the eQTL genes for rs1064395. d. Relative expression of the eQTL gene for rs3130253. E. Relative expression of the eQTL gene for rs2234978.

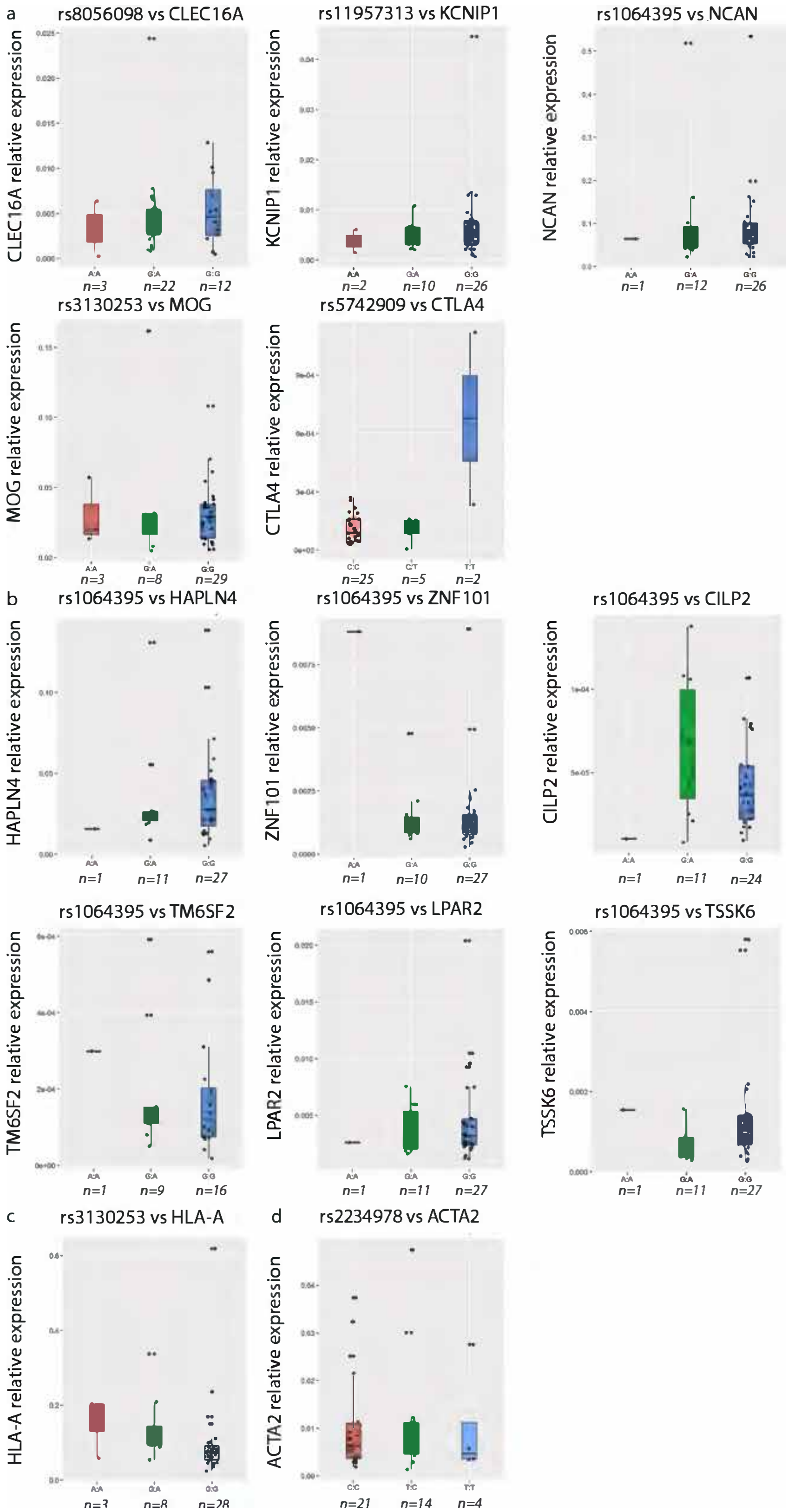

Supplement: Supplementary file 12 — Figure S4. Relative gene expression levels of eQTL genes that were not significantly related to the MS pathology‐associated SNPs (PDF). [file BPA-30-106-s010.pdf]
