# Supplementary material for: Post‐mortem multiple sclerosis lesion pathology is influenced by single nucleotide polymorphisms
Source: Brain Pathol. 2019 Jul 23;30(1):106–19. doi: 10.1111/bpa.12760 (PMC6916567; doi:10.1111/bpa.12760)
Supplement: Supplementary file 13 — Figure S5. Flow‐cytometric analysis of lymphocytes derived from peripheral blood and brain (PDF). [file BPA-30-106-s009.docx]

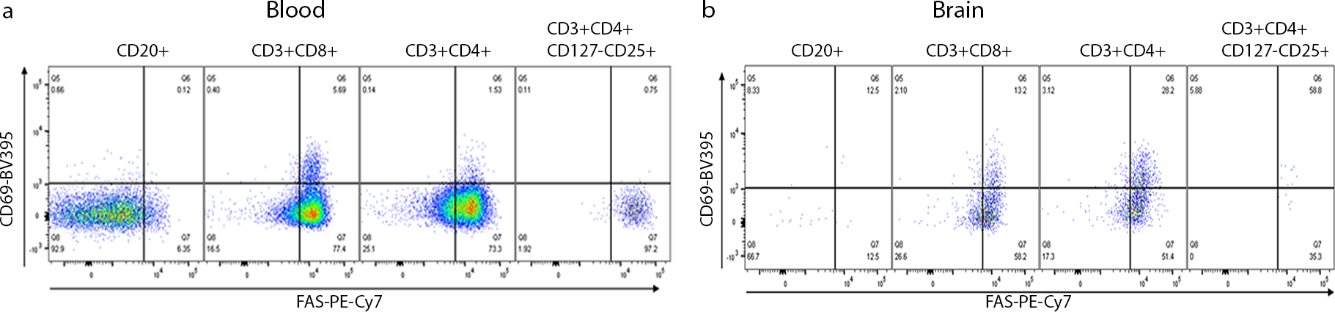


**Supplementary figure 5**. Flow-cytometric analysis of lymphocytes derived from peripheral blood and brain

a. Gating strategy for flow-cytometric analysis of blood samples. b. Gating strategy for flow-cytometric analysis of brain samples. Only rare events could be recorded in the population enriched for Treg cells (CD3+CD4+CD127-CD25+; n=7/4,604, n=2/425 and n=17/1,957 of CD4+ T cells, respectively), as well as B cells (CD20+; n=24, n=42 and n=501).
